# Selective Brain-Penetrant TTBK1 Inhibitors Modulate TDP-43 Pathology and Rescue Cognitive Deficits in a Mouse Model of TDP-43 Proteinopathy

**DOI:** 10.1021/acs.jmedchem.6c01882

**Published:** 2026-07-28

**Authors:** Cecilia Sanchez-Santos, Alberto Jimenez-Amor, Loreto Martinez-Gonzalez, Vanesa Nozal, Raquel Martin-Morales, Elnaz Aledavood, Daniel Bausela, Kayla Merrigan, Karen Diaz-Palacios, Rezart Zhubi, Stefan Knapp, Francesc R. Garcia-Gonzalo, Carmen Rodriguez-Cueto, Carmen Gil, Eva de Lago, Ana Martinez

**Affiliations:** † 54446 Centro de Investigaciones Biologicas “Margarita Salas”—CSIC, Ramiro de Maeztu 9, 28040 Madrid, Spain; ‡ Centro de Investigación Biomédica en Red en Enfermedades Neurodegenerativas (CIBERNED), Instituto de Salud Carlos III, Avda. Monforte de Lemos 3-5, 28029 Madrid, Spain; § Instituto Universitario de Investigación en Neuroquímica, Departamento de Bioquímica y Biología Molecular, Facultad de Medicina, Universidad Complutense, 28040 Madrid, Spain; ∥ Instituto Ramón y Cajal de Investigación Sanitaria (IRYCIS), 28034 Madrid, Spain; ⊥ 70694 Instituto de Investigaciones Biomédicas “Sols-Morreale”—UAM/CSIC, Arturo Duperier 4, 28029 Madrid, Spain; # Centro de Investigación Biomédica en Red de Enfermedades Raras (CIBERER), Instituto de Salud Carlos III, Avda. Monforte de Lemos 3-5, 28029 Madrid, Spain; ∇ Departamento de Bioquímica, Facultad de Medicina, Universidad Autónoma de Madrid (UAM), 28049 Madrid, Spain; ○ Institute of Pharmaceutical Chemistry, 9173 Goethe University, Max-von-Laue-Str. 9, 60438 Frankfurt am Main, Germany; ◆ Structural Genomics Consortium (SGC), Buchmann Institute for Molecular Life Sciences, Max-von-Laue-Str. 15, 60438 Frankfurt am Main, Germany

## Abstract

Transactive response DNA-binding protein of 43 kDa (TDP-43) is a pathological hallmark of neurodegenerative disorders, including amyotrophic lateral sclerosis (ALS) and frontotemporal dementia (FTD). Modulation of TDP-43 pathology represents a promising disease-modifying strategy. Tau tubulin kinase 1 (TTBK1) has emerged as a relevant therapeutic target; however, selectivity over the TTBK2 isoform is required to avoid ciliogenesis-related liabilities. Here, we report the discovery of selective, brain-penetrant TTBK1 inhibitors through a structure-guided medicinal chemistry program. Lead compounds exhibit potent and selective TTBK1 inhibition, no impact on ciliogenesis, and central nervous system exposure. We found that these inhibitors reduce TDP-43 phosphorylation levels in neuroblastoma cells and FTD patient-derived models. The optimized lead compound demonstrated a brain-to-plasma ratio of 3:1, a maximum tolerated dose, and a wide therapeutic window. *In vivo*, administration restored cognitive deficits, conferred neuroprotection in the frontal cortex, and reduced microglial activation in an FTD-TDP mouse model, supporting its therapeutic potential.

## Graphical abstract

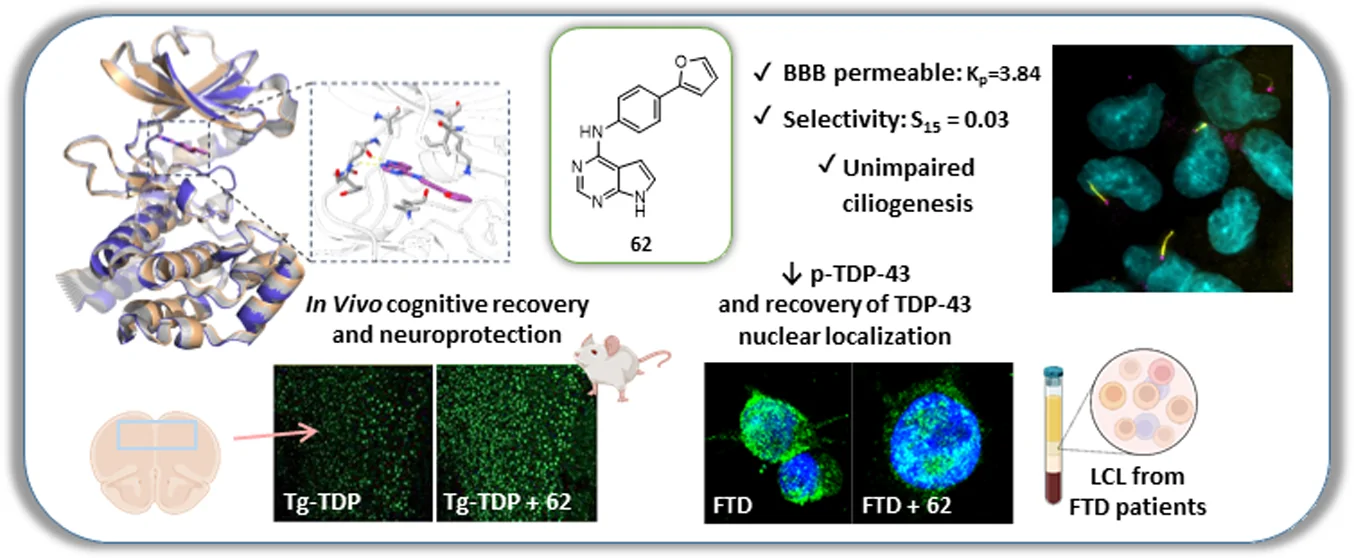

## Introduction

The aggregation of the transactive response DNA-binding protein of 43 kDa (TDP-43) defines a spectrum of neurodegenerative diseases collectively known as TDP-43 proteinopathies.^1^ This group prominently includes amyotrophic lateral sclerosis (ALS), frontotemporal dementia (FTD-TDP),^2^ and the more recently characterized limbic-predominant age-related TDP-43 encephalopathy (LATE),^3^ in which TDP-43 cytoplasmatic inclusions are primary pathological hallmarks. Also, mutations in the *TARDBP* gene, encoding TDP-43, are genetically linked as risk factors underlying ALS-FTD spectrum development.^4^ TDP-43 is a highly conserved, ubiquitously expressed RNA-binding protein essential for RNA metabolism, including transcription, splicing, transport, and stability via stress granule assembly.^5^ The TDP-43 structure comprises two RNA-recognition motifs (RRMs), a nuclear localization signal (NLS), and a glycine-rich, intrinsically disordered C-terminal domain involved in protein–protein interactions, which harbors the majority of disease-associated mutations and phosphorylation sites.^6^ Under physiological conditions, TDP-43 is primarily located in the nucleus, with tightly regulated expression via an autoregulatory feedback loop.^7^ However, under pathological conditions, TDP-43 undergoes nuclear depletion and subsequent cytoplasmic sequestration, where it undergoes several post-translational modifications, including fragmentation, acetylation, ubiquitination and hyperphosphorylation. These modifications reduce its solubility and promote the formation of pathological inclusions. Thus, the disruption of TDP-43 homeostasis may drive neurodegeneration through a combination of aggregate-mediated gain-of-function and nuclear loss-of-function toxicities.^8^

TDP-43 phosphorylation is driven by several kinases, most notably cell division cycle kinase 7 (CDC7),^9^ casein kinase 1 (CK1),^10^ and tau tubulin kinase 1 (TTBK1),^11^ which target C-terminal serine epitopes linked to pathological phosphorylation for instance at Ser409/410.^12^ However, the overall extent to which each of these kinases inhibition reduces protein phosphorylation remains unclear. Among these, TTBK1 has emerged as a promising therapeutic target for neurodegenerative diseases due to its neuron-specific expression^13^ and its role in the pathogenesis conditions of TDP-43 and tau^14^ This stands in contrast to its ubiquitously expressed isoform, tau tubulin kinase 2 (TTBK2), which is involved in several cell processes, including mitosis,^15^ neurotransmission,^16,17^ and ciliogenesis.^18,19^ Notably, mutations in the *TTBK2* gene cause spinocerebellar ataxia type 11 (SCA11), a rare inherited neurological disorder.^20^ In consequence, homozygous TTBK2 knockout mice carrying a loss-of-function mutation that caused a premature stop codon at residue 450 are lethal to embryos by day 10, exhibiting severe defects in brain development and poorly defined brain segmentation, whereas the heterozygous mutant TTBK2^fmly/+^ mice have a regular lifespan and normal brain development.^21^ In sharp contrast, transgenic mice expressing human full-length TTBK1 show different neurological dysfunctions such as tau phosphorylation, accumulation of neurofilaments, astro- and microgliosis, and other events linked with the additional potential role of TTBK1 in the regulation of CDK5.^22^ Moreover, the double-transgenic mice expressing TTBK1 and frontotemporal dementia with parkinsonism-17–linked P301L (JNPL3) tau mutant (TTBK1/JNPL3) have and increased accumulation of oligomeric tau protein in the central nervous system and enhanced loss of motor neurons in the ventral horn of the lumbar spinal cord.^23^ These results highlight that TTBK2 activity is essential for normal brain function, while TTBK1 is primarily related to disease conditions. It is therefore not surprising that recent studies linked nonisoform selective inhibition of TTBK with adverse effects derived from ciliogenesis dysfunction, underscoring the necessity for selective TTBK1 inhibitors.^24^

TTBK1 and TTBK2 are serine/threonine and tyrosine kinases belonging to the CK1 superfamily. Structurally, both kinases share a highly conserved N-terminal kinase domain with 88% identity and 96% similarity. In contrast, their noncatalytic regions differ significantly, as reflected by a reduced (35%) sequence identity and 63% similarity of their full-length sequences, which explains their distinct functions and tissue distributions (Figure 1).^25^ However, the intrinsically disordered nature of both TTBK1 and TTBK2 extra-catalytic regions explains why only the kinase domain structures have been resolved to date, several of which have been cocrystallized with ATP or ATP-competitive inhibitors.

**Figure 1:**
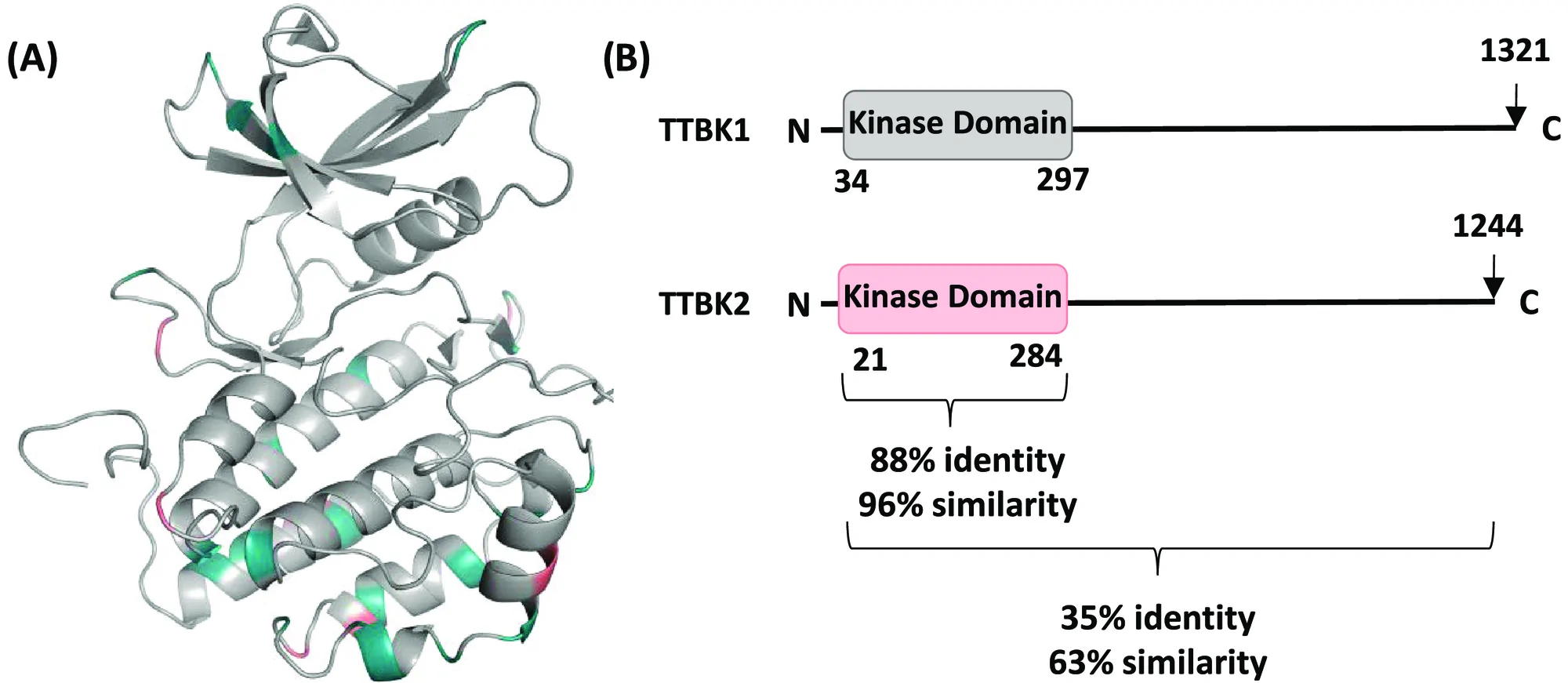
(A) Crystal structure of the human TTBK1 kinase domain (PDB ID: 4BTJ).^26^ Residues are colored according to sequence conservation relative to human TTBK2: conserved residues are shown in gray, similar residues in blue, and divergent residues in red. (B) Schematic representation of full-length TTBK1 and TTBK2 proteins.

To date, all reported and crystallized small molecules targeting TTBK1 protein are type I inhibitors, binding at the active site in an ATP-competitive manner. These small molecules interact with the hinge region, while some also establish additional contacts with other residues within the active site (Figure 2). The first TTBK inhibitors that have been described were developed by AstraZeneca and demonstrated equipotent, low-micromolar affinity for both TTBK1 and TTBK2 (**AZ-1**: TTBK1 IC_50_ = 4.4 μM, TTBK2 IC_50_ = 6.8 μM; **AZ-2**: TTBK1 IC_50_ = 2.6 μM, TTBK2 IC_50_ = 3.2 μM).^26^ A year later, Bristol-Myers Squibb reported **2KC**, a pyrrolotriazine derivative with significantly enhanced potency for TTBK1, however also this inhibitor lacked isoform-selectivity (TTBK1 IC_50_ = 120 nM, TTBK2 IC_50_ = 170 nM).^27^ A significant turning point occurred with the development of the first TTBK1 inhibitors that demonstrated *in vivo* efficacy such as azaindole **TTBK1-IN-1**,^28^ and our previously developed pyrrolopyrimidine **TTBK1-IN-2**.^29^ Both compounds exhibited suitable central nervous system (CNS) penetration and effectively reduced tau and TDP-43 phosphorylation, respectively, at disease-relevant sites in different mouse models. These findings demonstrated the therapeutic potential of TTBK1 inhibition for treating proteinopathy-driven neurodegenerative diseases. Notably, while **TTBK1-IN-1** inhibits TTBK1 and TTBK2 equipotently (TTBK1 IC_50_ = 2.7 nM, TTBK2 IC_50_ = 1.6 nM), **TTBK1-IN-2** offers a more favorable selectivity profile for TTBK1 (TTBK1 IC_50_ = 240 nM, TTBK2 IC_50_ = 4.22 μM), proving the feasibility of achieving selectivity for one of the TTBK isoforms. The work in this study therefore focused on the development of novel TTBK1 inhibitors derived from the **TTBK1-IN-2** scaffold, with the aim to improve inhibitor potency and selectivity, developing a preclinical lead for the treatment of TDP-43-related proteinopathies without the adverse effects associated with TTBK2 inhibition.

**Figure 2:**
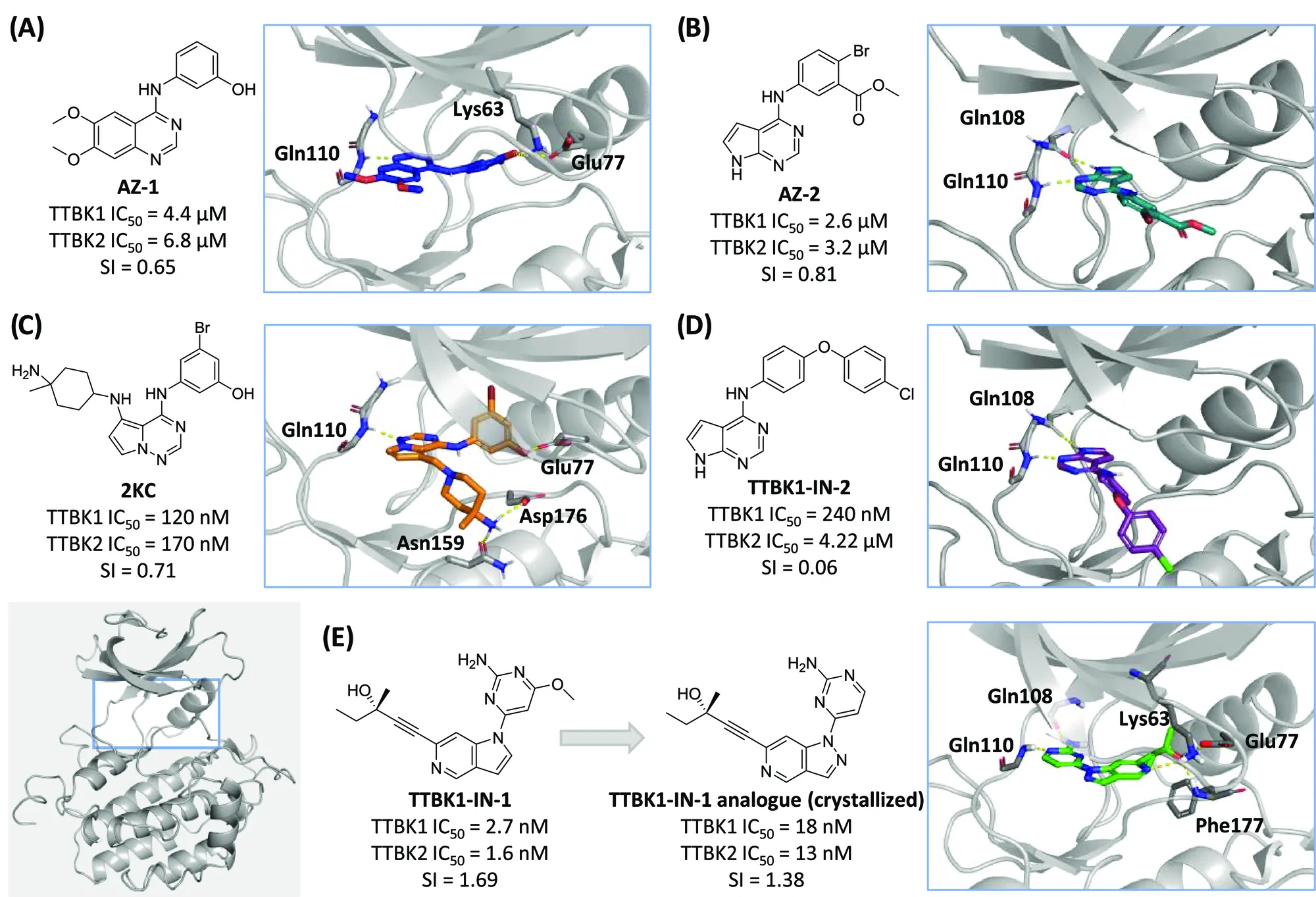
Crystal structures of the TTBK1 kinase domain in complex with reported inhibitors. The panels show the binding modes and inhibitory activities (IC_50_) for TTBK isoforms, along with the selectivity index (SI): (A) **AZ-1** (PDB ID: 4BTK),^26^ (B) **AZ-2** (PDB ID: 4BTM),^26^ (C) **2KC** (PDB ID: 4NFN),^27^ (D) **TTBK1-IN-2** (PDB ID: 7QHW),^29^ (E) binding mode of the **TTBK1-IN-1** series (PDB ID: 7JXY).^28^

## Results and Discussion

### Design and Synthesis of TTBK1 Inhibitors

In our pursuit of developing isoform-selective TTBK1 inhibitors, we embarked on this project by expanding upon our previously reported chemical series, which includes **TTBK1-IN-2**. Based on the superior affinity exhibited by the pyrrolo­[2,3-*d*]­pyrimidine scaffold for TTBK1 compared with other purine analogues described in previous work,^29^ we decided to retain this [6 + 5] bicyclic core while exploring modifications on the pendant aromatic moiety.

Through this approach, we identified compound **1**, a new pyrrolopyrimidine derivative featuring a biphenyl system in place of the ether-linked phenyl groups of **TTBK1-IN-2** series. Specifically, derivative **1** bears a 4-methoxy group on the distal phenyl ring and was synthesized via nucleophilic aromatic substitution (S_N_Ar) using the commercial reagents 4′-methoxy-[1,1′-biphenyl]-4-amine and 4-chloro-7*H*-pyrrolo­[2,3-*d*]­pyrimidine (Scheme 1 and Table 1). Despite the moderate TTBK1 potency of **1** (IC_50_ = 13.8 ± 0.6 μM), this molecule displayed selectivity over TTBK2 (4.2% of TTBK2 inhibition at 10 μM) with a selectivity index (SI) of 0.14 (SI = IC_50_ TTBK1/IC_50_ TTBK2. Consequently, further optimization was directed toward the distal aromatic ring of the biphenyl system to enhance inhibitory potency, while the core structural arrangement was maintained. To this end, a systematic series of analogues was designed introducing different substituents on the distal phenyl ring, including halogens, hydroxyl, alkoxy, acetoxy, amino, and nitro groups. Additionally, the distal phenyl ring was replaced with various heterocycles, such as furan, thiophene, pyrrole, pyrazole, and indazole moieties. While most of the derivatives maintained the *para*-disposition of the biphenyl system, the *meta* and *ortho* orientations were also briefly explored (Figure 3).

**Figure 3:**
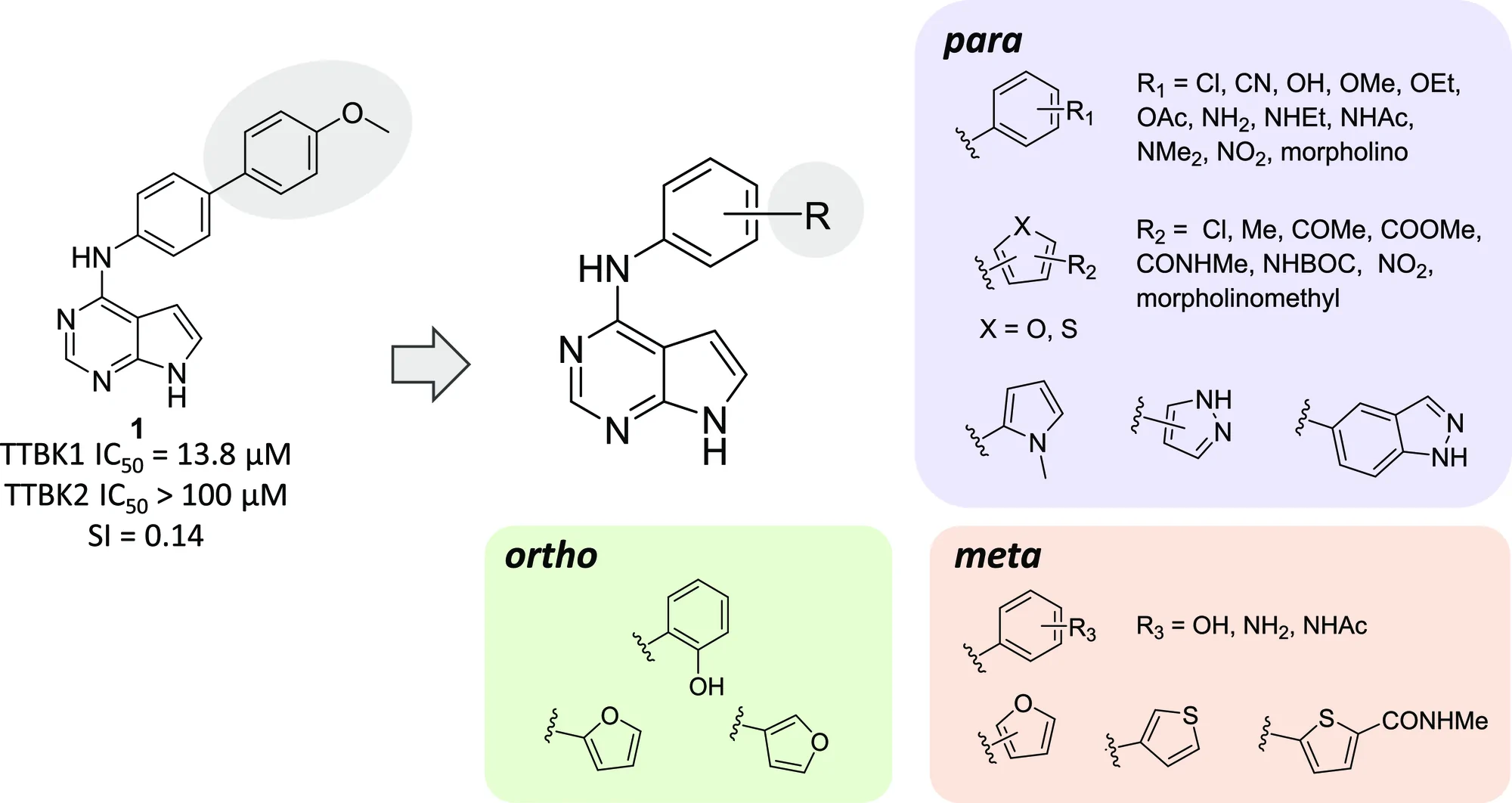
Chemical structure of hit **1** and structural modifications explored in this study focused on the biphenyl moiety. Derivatives were categorized based on the substitution pattern of the distal aromatic ring R: *ortho* (green), *meta* (orange), and *para* (purple) positions.

**Scheme 1:**
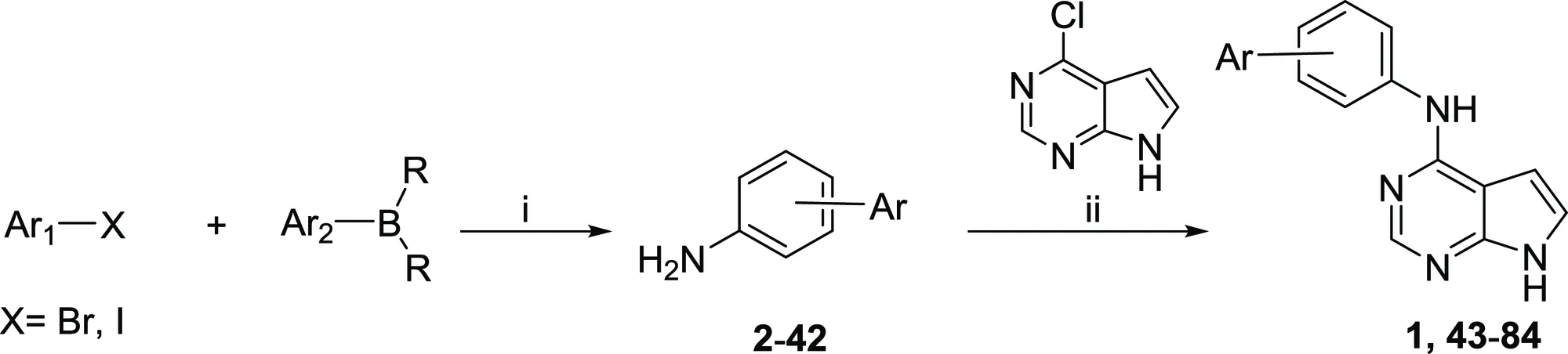
Synthesis of Intermediates **2**–**42** and Derivatives **1** and **43**–**84**: (i) K_3_PO_4_ or Na_2_CO_3_, Pd­(PPh_3_)_4_, Toluene/H_2_O/EtOH, MW, 120 °C, 20 min; (ii) InCl_3_, MeCN or THF, MW, 120 °C

**Table 1:**
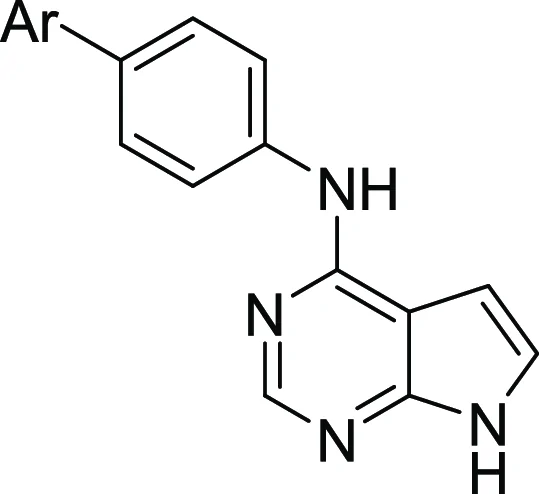

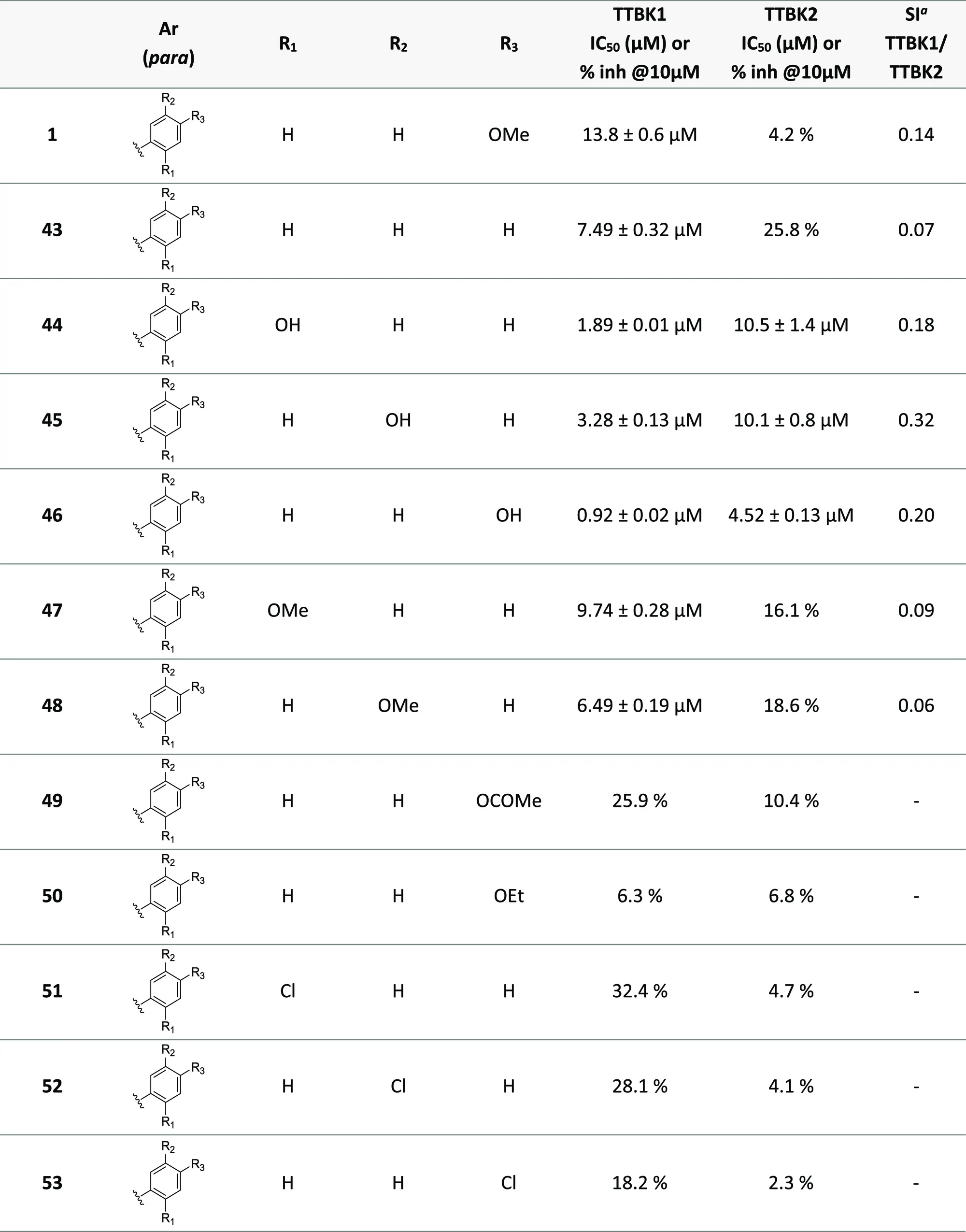

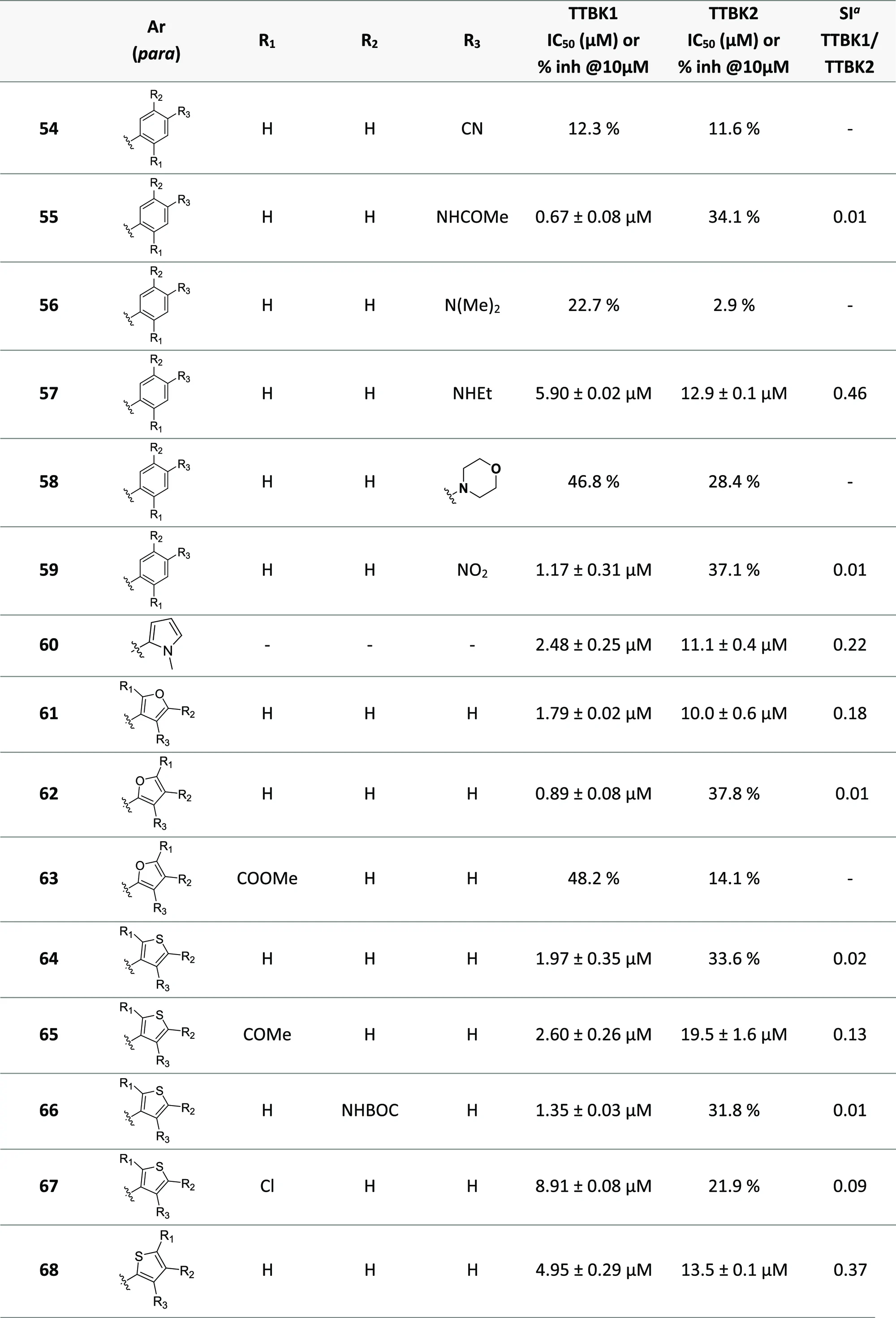

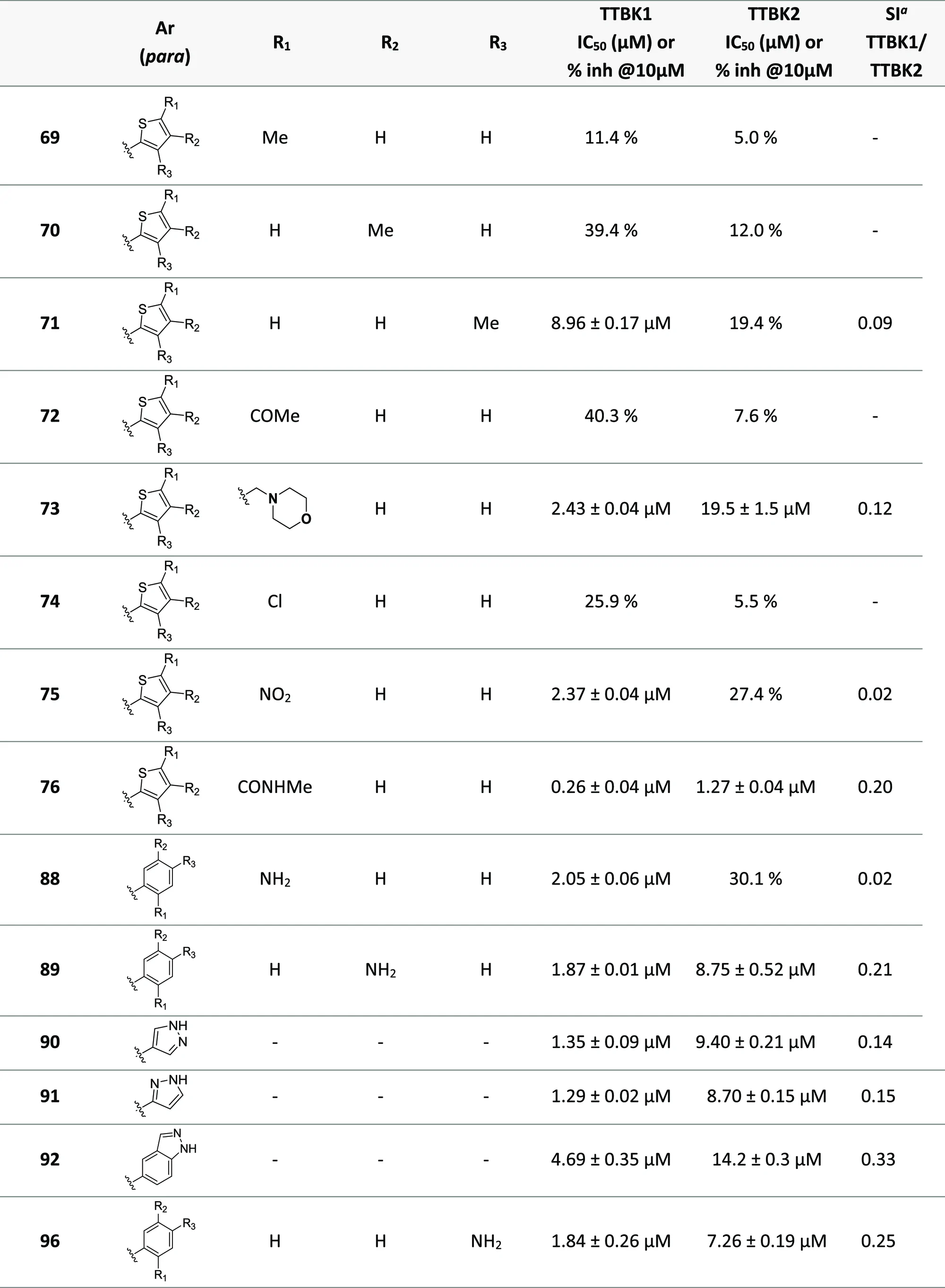
Chemical Structure, and TTBK Inhibition Data for *para*-Orientated Derivatives **1**, **43**–**76**, **88-92**, and **96**

a When the % of inhibition is below 50%, an IC_50_ value of 100 μM is considered.

The general synthetic route comprised a two-step sequence, as illustrated in Scheme 1. Initially, the aniline-derived biphenyl intermediates **2**–**42** were synthesized via Suzuki cross-coupling between the appropriate aromatic halides and their corresponding pinacol boronate or boronic acid derivative. The selection of these starting precursors detailed in the experimental section was dictated by commercially available reagents, ensuring that, in all cases, one moiety incorporated the amino group required for the subsequent S_N_Ar. These Suzuki couplings were catalyzed by Pd­(PPh_3_)_4_ in the presence of K_3_PO_4_ or Na_2_CO_3_ as the base, typically affording the desired intermediates with moderate yields. Subsequently, the target compounds **43**–**84** (Scheme 1 and Tables 1–3) were obtained via S_N_Ar between the respective biphenyl intermediate and 4-chloro-7*H*-pyrrolo­[2,3-*d*]­pyrimidine. To facilitate the nucleophilic aromatic substitution, it was assisted by microwave irradiation, and indium trichloride (InCl_3_) was employed as a Lewis acid to activate the π-deficient heterocycles. However, the reactions generally suffered from modest yields due to the reduced nucleophilicity of the amino group, a consequence of electronic delocalization across the biphenyl arrangement. This effect was aggravated when the distal ring was substituted with electron-withdrawing groups, which further deactivated the aniline moiety toward nucleophilic attack.

In two specific scenarios, the order of the synthetic steps was inverted to ensure functional group compatibility. In particular, this alternative route was employed when the distal aromatic ring bore a nucleophilic substituent that may interfere with the S_N_Ar, or when the biphenyl intermediate proved unstable during the synthesis. Consequently, the S_N_Ar reaction was performed in first place between the corresponding haloaniline derivatives and 4-chloro-7*H*-pyrrolo­[2,3-*d*]­pyrimidine to afford compounds **85**–**87** (Scheme 2) with good yields. Subsequently, a Suzuki cross-coupling reaction with these intermediates and their appropriate pinacol boronate or boronic acid derivatives yielded compounds **88**-**95** (Scheme 2 and Tables 1–2). Reduction of the nitro group in compound **59** using SnCl_2_ was performed exclusively to obtain the symmetric compound **96**, thereby preventing dimerization through the two amino groups (Scheme 3 and Table 1).

**Scheme 2:**
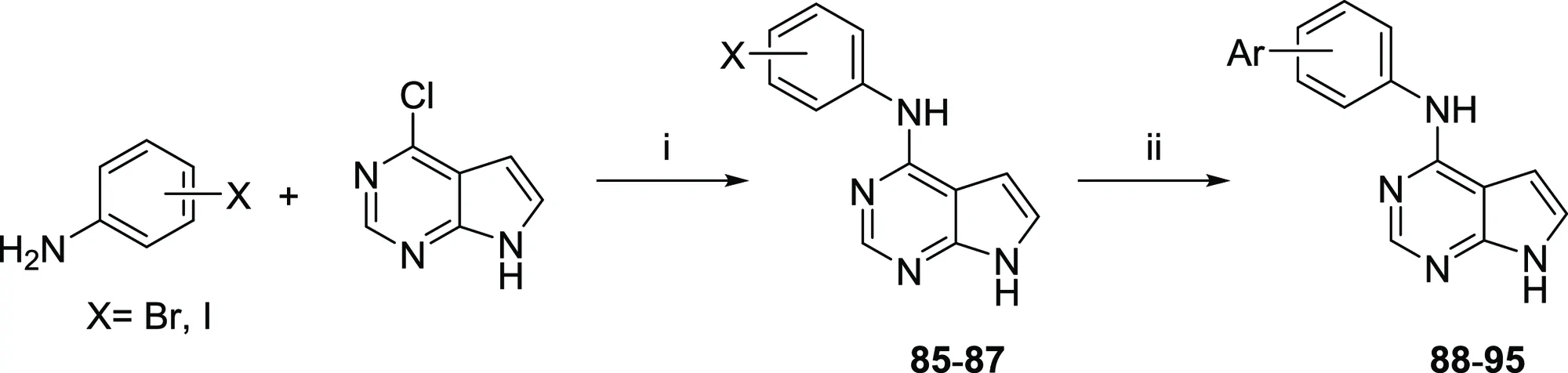
Synthesis of Intermediates **85**–**87** and Derivatives **88**–**95:** (i) InCl_3_, MeCN or THF, MW, 120 °C; (ii) Pinacol Boronate or Boronic Acid Derivative, K_3_PO_4_ or Na_2_CO_3_, Pd­(PPh_3_)_4_, Dioxane/H_2_O/EtOH, MW, 120 °C

**Scheme 3:**
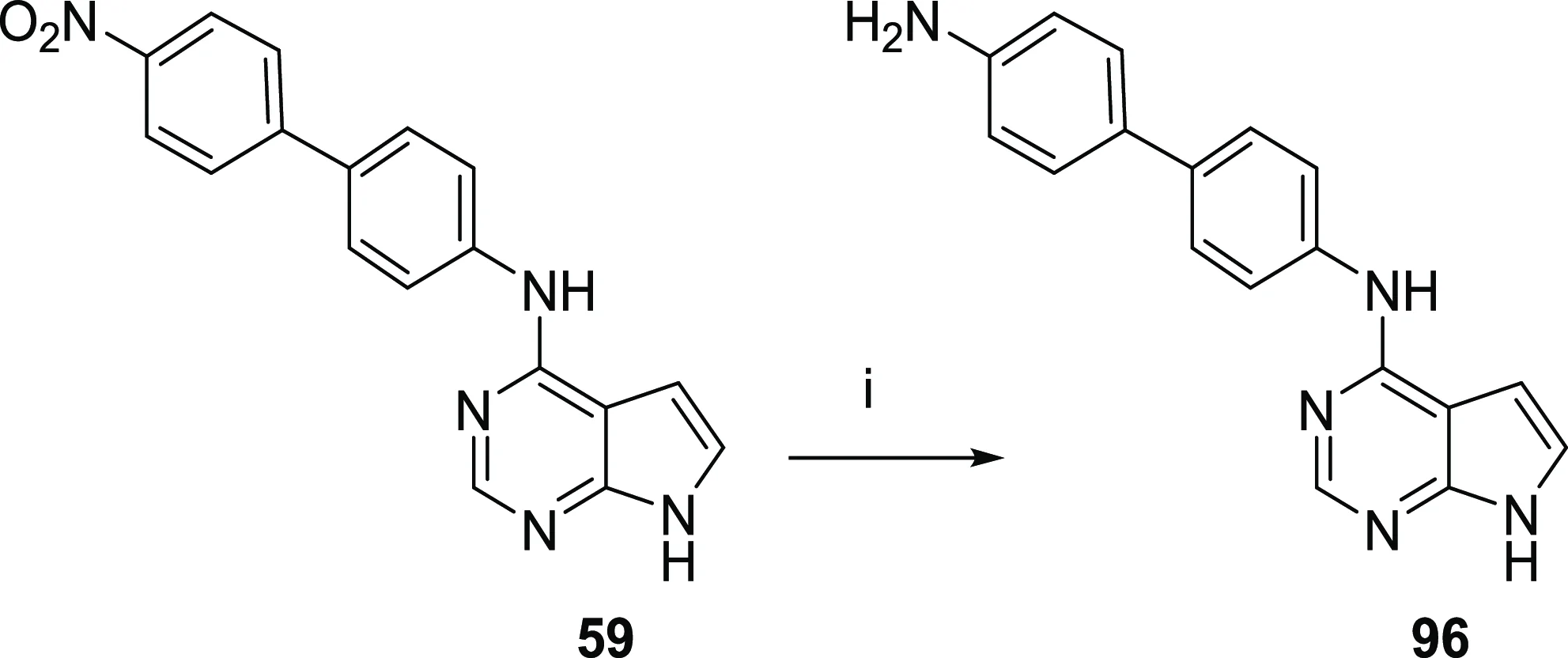
Synthesis of Derivative **96**: (i) SnCl_2_·2H_2_O, HCl 37%, Ethanol, MW, 120 °C

**Table 2:**
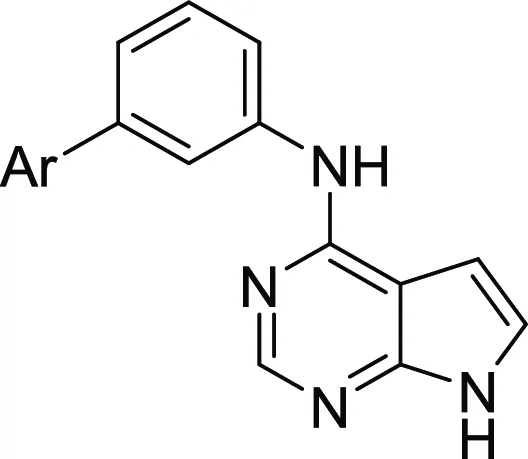

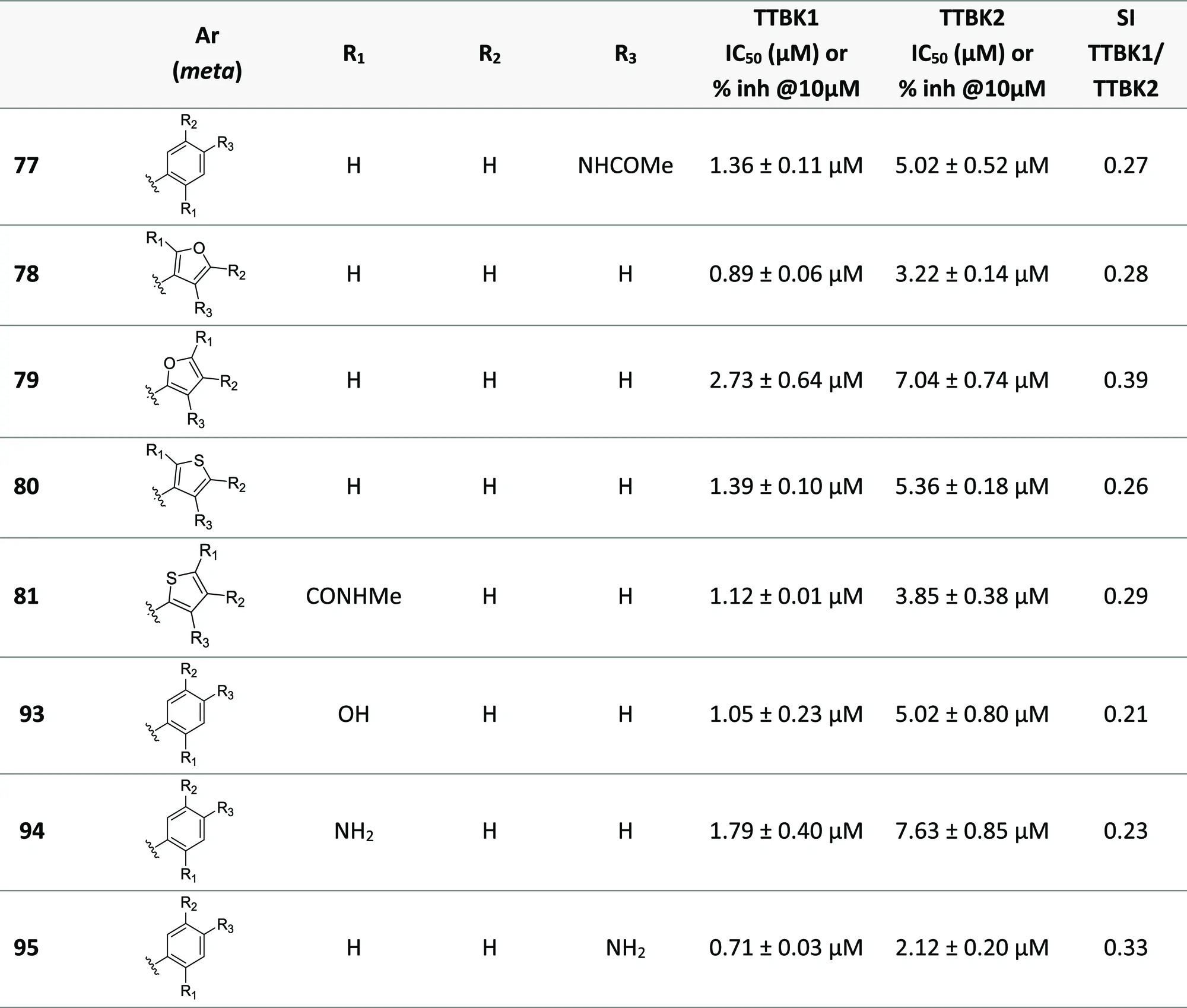
Chemical Structure, and TTBK Inhibition Data for *meta*-Orientated Derivatives **77**–**81** and **93**–**95**

The inhibitory activities of the synthesized compounds were determined through Kinase-Glo luminescent assay using the purified kinase domain of TTBK1 or TTBK2, a specific phosphorylation substrate and ATP at 1 μM, as described in more detail in the Experimental Section. First, the compounds were evaluated at 10 μM, and when the inhibition of the enzyme were above 50%, a dose–response curve was performed to determine the IC_50_ value. The commercial **TTBK1-IN-1** inhibitor was used as reference compound (Figure S1).

The SAR exploration was initiated by evaluating the influence of various substituents on the distal aromatic ring on our largest explored series of *para*-orientated biphenyl derivatives. As summarized in Table 1, derivatives featuring a hydroxyl group at any position (**44**, **45**, and **46**) exhibited superior potency (IC_50_ ∼ 1–3 μM) compared with the methoxy (**1**, **47**, and **48**) and ethoxy (**50**) analogues with IC_50_ above 6 μM. The introduction of a hydrogen-bond acceptor (HBA) in *para*-position, such as a nitrile moiety (**54**), resulted in a complete loss of inhibitory activity for both TTBK1 and TTBK2. This highlights the crucial role of hydrogen-bond donor (HBD) groups for TTBK1 inhibition.

In the study of alternative HBD substituents, the replacement of the hydroxyl group with an amino group slightly altered TTBK1 inhibitory activity (**88**, **89**, and **96**), yielding IC_50_ values in the low micromolar range while maintaining excellent TTBK2 selectivity, particularly for the *ortho*-amino-substituted analogue **88** (SI = 0.02). Consistent with the hydroxyl derivatives, *N*-alkylation of the amino group resulted in a significantly decreased potency: the *N*,*N*-dimethyl (**56**), *N*-ethyl (**57**), and morpholino (**58**) derivatives were markedly less active or inactive.

Notably, the introduction of an acetamido substituent significantly improved the potency (**55**, TTBK1 IC_50_ = 0.67 μM) while ensuring outstanding selectivity (SI < 0.01). This enhancement was strictly dependent on the amino-carbonyl pair, as the corresponding acetate (**49**) and *N*-ethylamino (**57**) analogues were substantially less potent. These findings highlight the key role of the amide linkage, where a carbonyl could be contributing additional hydrogen-bond interactions or a more favorable orientation of the NH donor within the TTBK1 binding pocket.

The impact of replacing the distal phenyl ring with various heterocycles was also explored. The inhibitory potency in TTBK1 resulted in the following ascending order: phenyl (**43**, IC_50_ = 7.49 μM), indazole (**92**, IC_50_ = 4.69 μM), *N*-methylpyrrole (**60**, IC_50_ = 2.48 μM), thiophene derivatives (**64**, IC_50_ = 1.97 μM; **68**, IC_50_ = 4.95 μM), furan derivatives (**61**, IC_50_ = 1.79 μM; **62**, IC_50_ = 0.89 μM), and pyrazole derivatives (**90**, IC_50_ = 1.35 μM; **91**, IC_50_ = 1.29 μM). Overall, the pi-excessive five-membered heterocycles thiophene, furan, and pyrazole enhanced TTBK1 inhibitory potency and isoform selectivity (with SI reaching values under 0.01) compared with the parent biphenyl scaffold, likely due to optimized electrostatic interactions and a more favorable orientation in the binding cavity.

Subsequent substitution in the thiophene and furan rings followed a SAR pattern consistent with the phenyl series. Consequently, the introduction of an amino-carbonyl moiety resulted in the most potent analogue: the *N*-methyl-carboxamide derivative **76** (TTBK1 IC_50_ = 260 nM; SI = 0.20). In contrast, the incorporation of chlorine atoms, methyl or acetyl groups at any position led to a valuable reduction in the inhibitory activity against both TTBK1 and TTBK2 (**65**, **67**, **69**-**72**, and **74**). This detrimental effect was also observed in the phenyl series, with derivatives **51**-**53**, suggesting that bulky or electron-withdrawing substituents without HBD capacity do not benefit the interaction of the ligand with the protein.

The influence of highly electron-withdrawing groups was further investigated through the synthesis of nitro-substituted analogues **59** and **75**, which exhibited very good isoform selectivity (SI = 0.01 and 0.02, respectively), but their moderate TTBK1 IC_50_ and poor aqueous solubility made them less interesting candidates for further development.

To assess the impact of the distal ring’s connectivity on the biphenyl scaffold, a series of *meta*-substituted biphenyl compounds were synthesized derived from the most potent and selective *para*-analogues (Table 2 and Table S1). While these *meta*-orientated biphenyls retained good TTBK1 inhibition, a significant reduction of selectivity against TTBK2 was observed in all cases, with SI reaching 0.20–0.40 values (Table S1). Given that achieving high TTBK-isoform selectivity was the primary objective of this project, further exploration of the *meta*-orientated biphenyls was discontinued.

Finally, the *ortho*-connectivity was briefly evaluated with compounds **82**–**84** (Table 3); however, these derivatives were found to be inactive, likely due to steric hindrances that prevent the necessary coplanarity or optimal binding orientation of the molecule within the biding site.

**Table 3:**
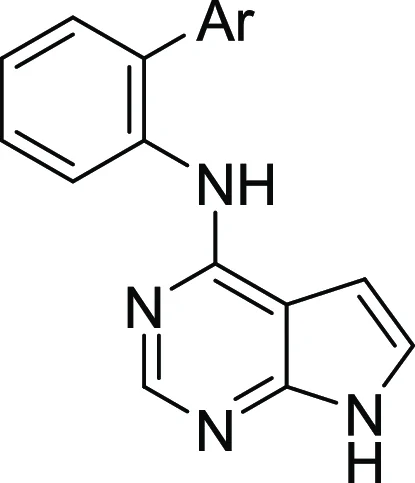

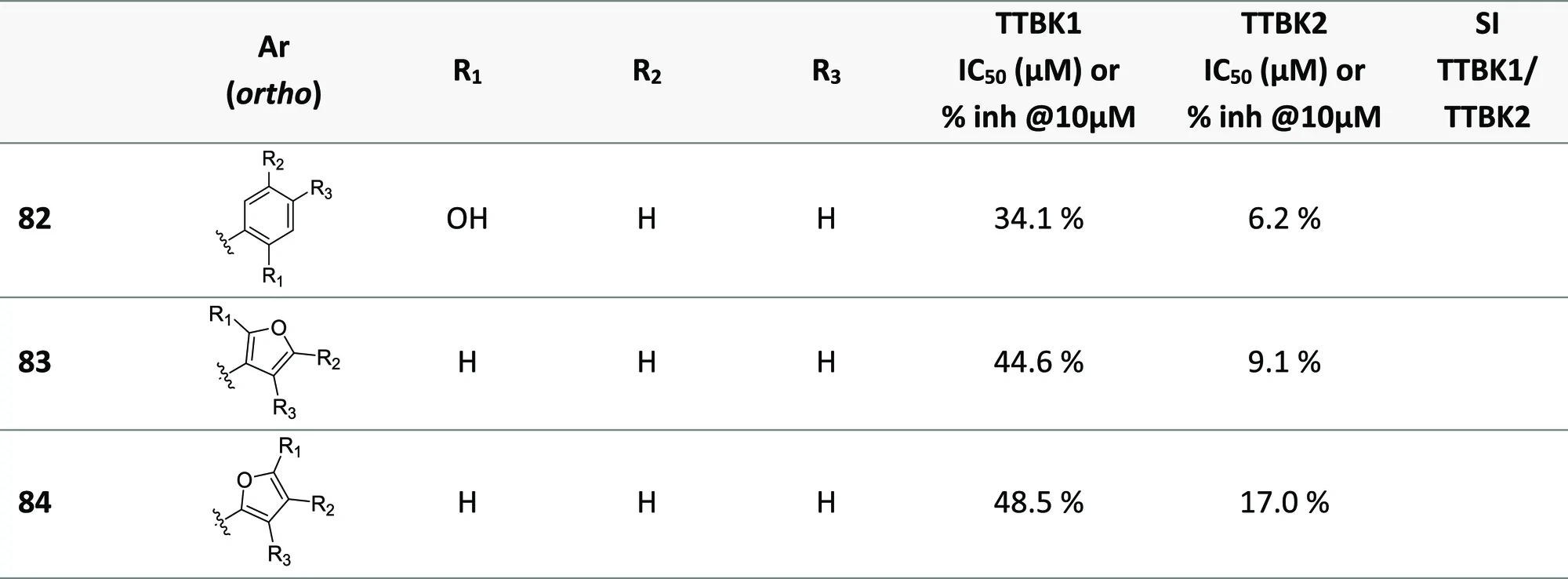
Chemical Structure and TTBK Inhibition Data for *ortho*-Orientated Derivatives **82**–**84**

### Blood-Brain Barrier Permeability

The ability of the most active and isoform-selective compounds (TTBK1 IC_50_ ≤ 3 μM and SI ≤ 0.25) to passively cross the blood-brain barrier (BBB) was evaluated early in this project, as this is a critical property required for these molecules to reach their target in the CNS. For this purpose, the parallel artificial membrane permeability assay (PAMPA) was employed using a porcine brain lipid membrane that emulates BBB properties and a PBS/EtOH (7:3) buffer system. Ten commercial drugs of known CNS permeability were included as internal standards as detailed in the Experimental Section. Worth mentioning, compounds **59** and **75** have low aqueous solubility, which hampered their evaluation under these assay conditions. Based on the results represented in Figure 4 and Table S2, among the most active and isoform-selective compounds evaluated, only compounds **46** and **90** exhibited a negative CNS penetration (CNS-), probably due to the high polarity of their hydroxyl group or pyrazole ring, respectively. Furthermore, the elevated polarity of compounds **55**, **65**, **76**, **89**, **91**, **93**, and **94** penalized their effective permeability values (*P*_e_), falling within the uncertainty area (CNS+/−) between the established permeable and nonpermeable thresholds. Compounds **44**, **60**, **61**, **62**, **64**, **66**, **73**, **88**, and **96** showed a favorable BBB permeability (CNS+) and were therefore selected to be further studied.

**Figure 4:**
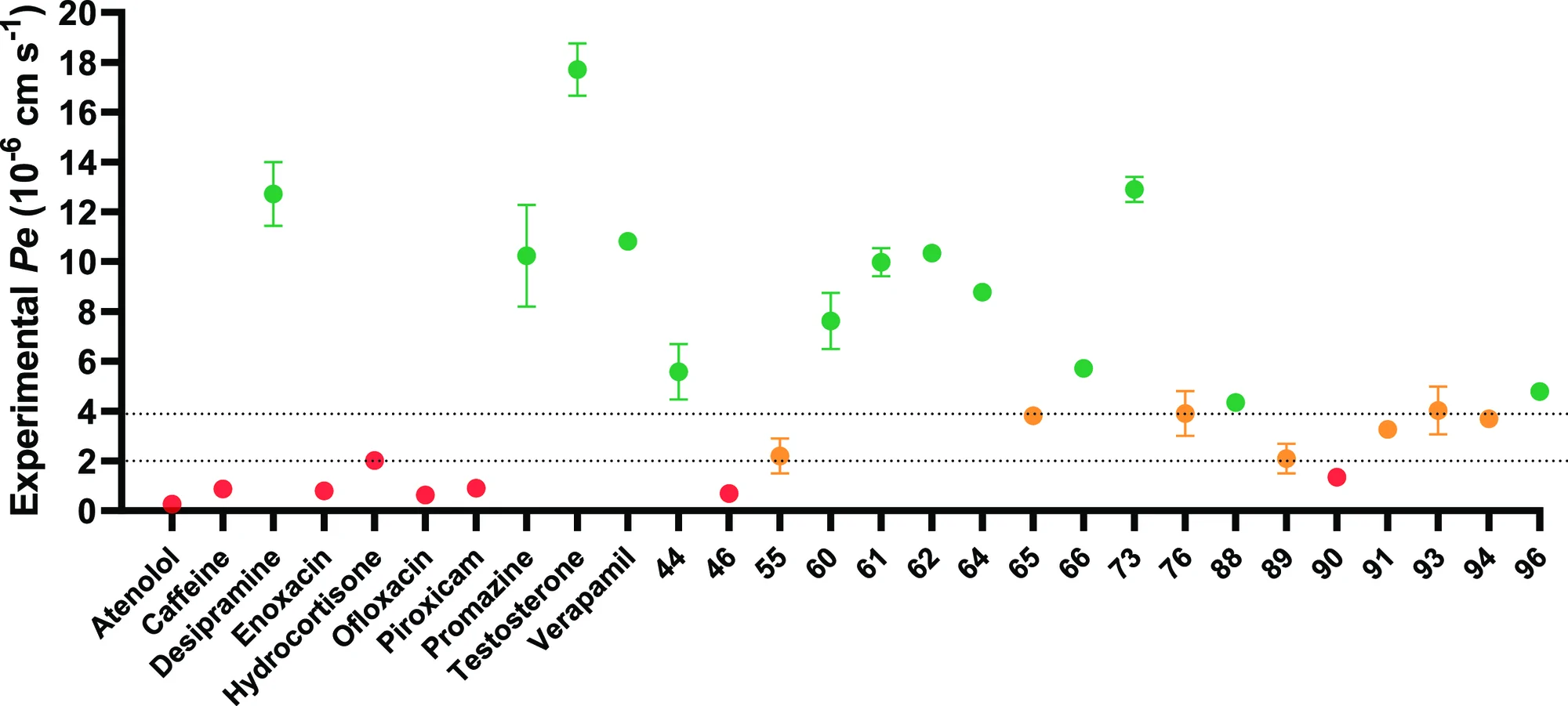
BBB permeability predictions for control drugs and TTBK1 inhibitors predicted using PAMPA. The dashed lines indicate the permeability thresholds separating the CNS+ (permeable, green), CNS +/– (intermediate/uncertain, orange), and CNS- (nonpermeable, red) predictions. Data represent the mean ± SD (*n* = 2) independent experiments.

### Evaluation of TTBK1 inhibitors in TDP-43 Cellular Models

Given that TTBK1 is a key kinase implicated in the pathological phosphorylation of TDP-43 at the epitopes Ser409/410,^9^ we evaluated the ability of nine selected candidates (**44**, **60**, **61**, **62**, **64**, **66**, **73**, **88**, and **96**)–those previously identified as isoform-selective and CNS-permeable in enzymatic and PAMPA assays–to modulate TDP-43 phosphorylation levels in the SH-SY5Y human neuroblastoma cell line. To this end, we employed a mammalian cell-based model in which the glutathione-depleting agent ethacrynic acid (EA) induced oxidative stress and TDP-43 phosphorylation, ultimately reducing cell viability.^30^ Accordingly, compounds were considered neuroprotective when pretreatment significantly preserved cell viability under EA insult. An initial MTT-based viability study was performed at three different concentrations for each candidate (Figure S2), and we eventually selected 1 μM as the safe working concentration for subsequent assays. Thus, cells were pretreated with compounds or the neuroprotective control Tideglusib, a well-established inhibitor of GSK3β with proven effectiveness in ALS models.^31,32^ After 1 h, cells were challenged with EA at 80 μM for 24 h, reducing cell viability to approximately 50% relative to untreated control. The results show that compounds **61**, **62**, and **64** conferred remarkable neuroprotection to levels comparable to those for the positive control, as shown in Figure 5A. In order to confirm that the neuroprotection observed is linked to a decrease in TDP-43 phosphorylation levels (pTDP-43), subsequent Western Blot analysis was performed. The signal intensities of both pTDP-43 and TDP-43 were first normalized to GAPDH, as the internal loading control for each sample. The relative phosphorylation ratio was then calculated as pTDP43/TDP-43 for treatment condition and subsequently normalized to the insult control (EA). In addition, total TDP-43 protein levels were evaluated following treatment with EA or the tested compounds. No statistically significant differences in the TDP-43/GAPDH ratio were observed under in any of the experimental conditions (Figure S3).

**Figure 5:**
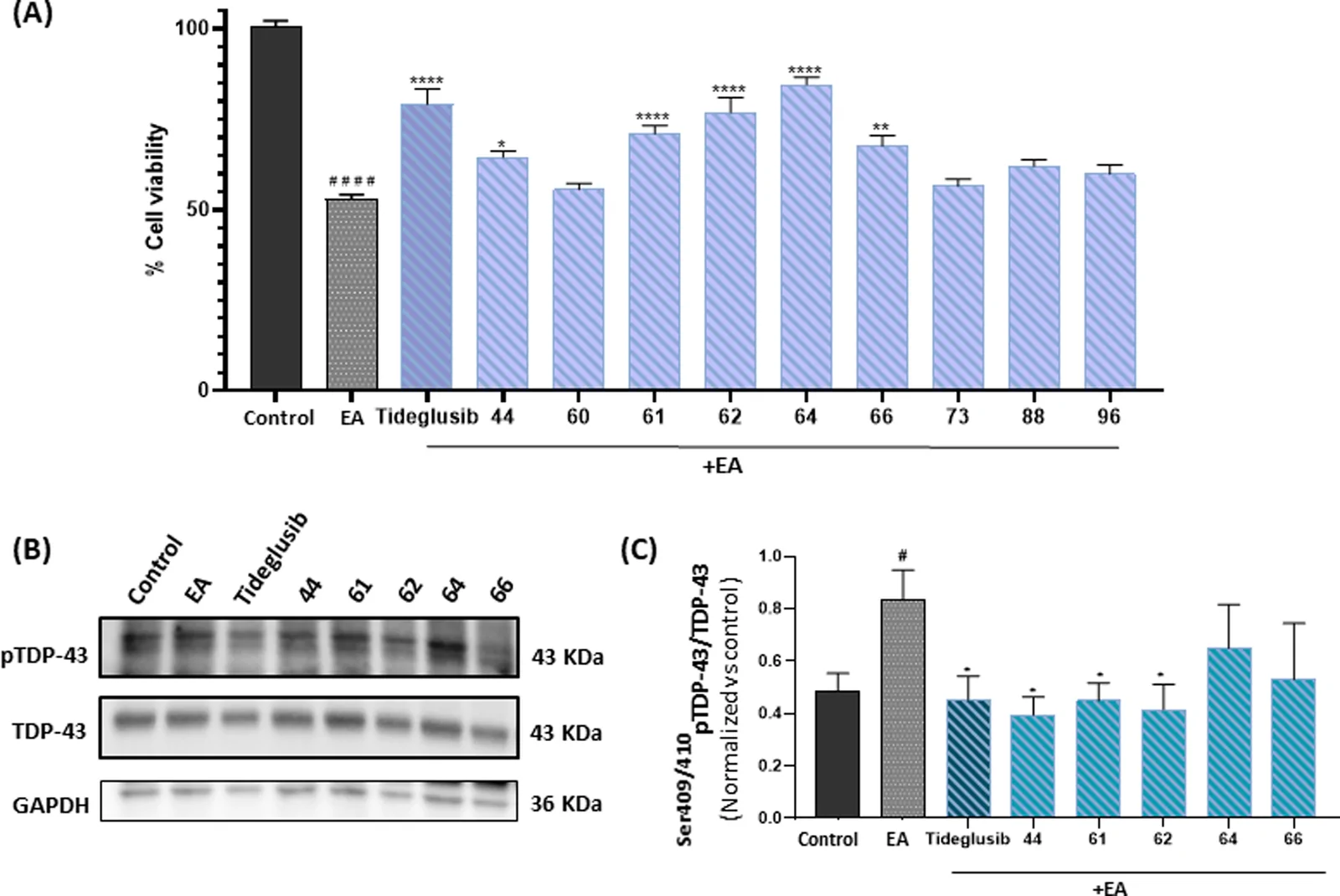
Effect of TTBK1 inhibitors on cell viability and TDP-43 phosphorylation in the ethacrynic acid (EA)-induced toxicity model in SH-SY5Y. Cells were exposed to EA (80 μM) for 24 h in the presence or absence of TTBK1 inhibitors (1 μM). (A) Cell viability was measured with the MTT assay. The GSK3β inhibitor Tideglusib (5 μM) was used as neuroprotective control. Data represent the mean ± SEM (*n* = 4). (B) Representative immunoblot of TDP-43 phosphorylation (Ser409/410) and total TDP-43 after treatment with candidates **44**, **61**, **62**, **64**, and **66** (1 μM), or Tideglusib (5 μM), 1 h prior exposure to EA. GAPDH was employed as a loading control. (C) Graph represents the quantification of pTDP-43/total TDP-43, expressed as mean ± SEM of *n* = 4 biological independent immunoblotting experiments. Statistical significance was performed using One-way ANOVA followed by the Fisher’s LSD test for multiple comparisons. (*p < 0.05, **p < 0.01, ****p < 0.0001 significantly different from SH-SY5Y EA-treated cells (−), ^#^ *p* < 0.05 ^####^ *p* < 0.0001 significantly different from SH-SY5Y positive control (+)).

The results displayed in Figure 5B,C revealed that compounds **44**, **61**, and **62** significantly decreased TDP-43 phosphorylation at Ser409/410 comparable to control cell levels and GSK3β inhibitor Tideglusib, which indicates that the neuroprotective effect exhibited in the EA cellular model is associated with pTDP-43 modulation. By contrast, candidates **64** and **66**, although conferring SH-SY5Y cell viability preservation, only indicated a slight, but not statistically significant, trend toward preventing TDP-43 from pathological phosphorylation.

Furthermore, the reduction in pTDP-43 levels, also observed and quantified by immunohistochemistry (Figure S3), was accompanied by a restoration of TDP-43 subcellular localization as immunofluorescence analysis revealed that, under EA insult, compounds **44** and **62** significantly restored nuclear TDP-43 localization compared with control conditions (Figure 6). These findings provide strong evidence supporting the therapeutic potential of these candidates to preserve TDP-43 homeostasis.

**Figure 6:**
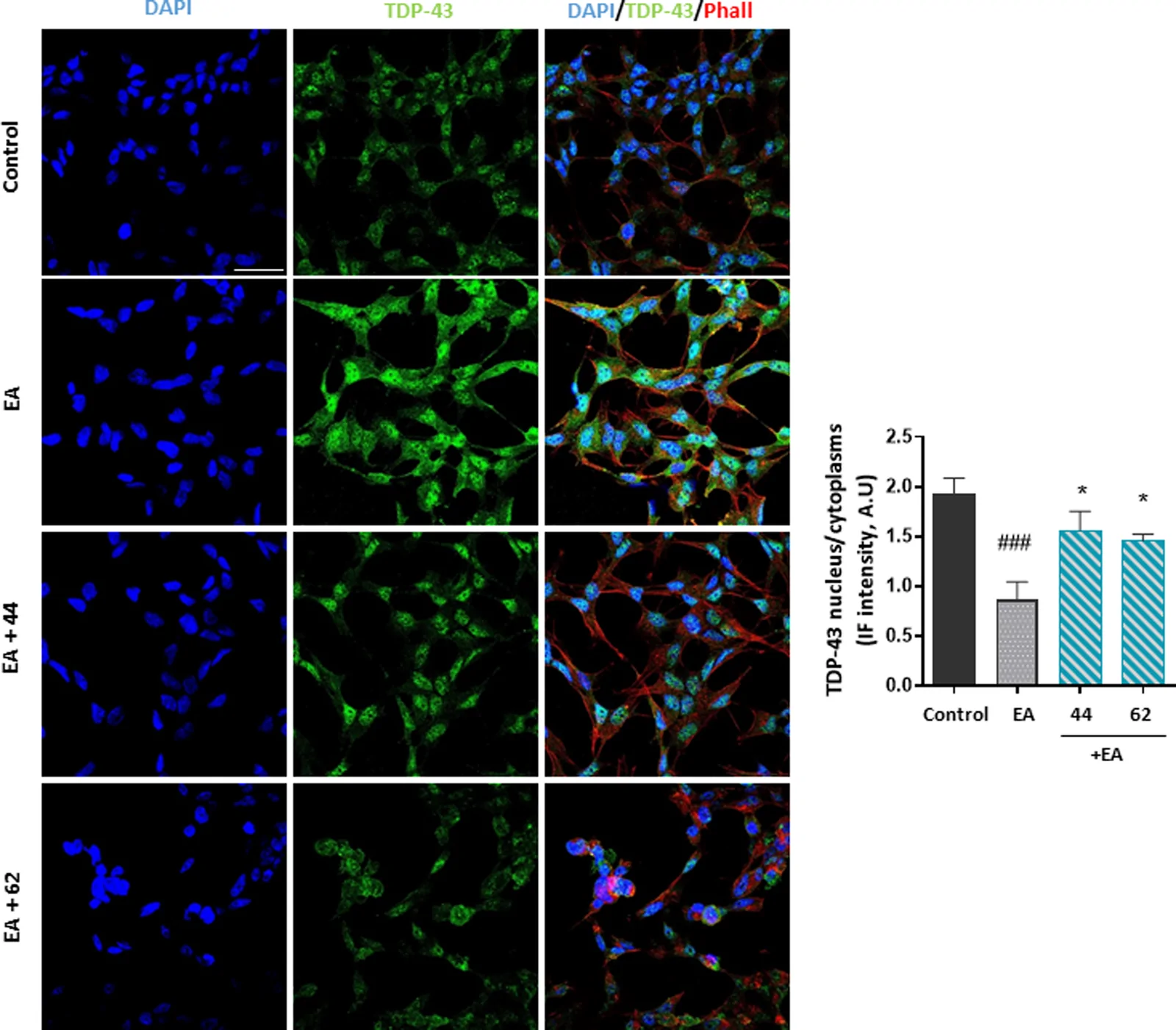
Effect of TTBK1 inhibitors on TDP-43 subcellular localization in SH-SY5Y cells under EA insult. Cells were pretreated with compound **44** and **62** (1 μM) for 1 h, then cells were exposed to EA (80 μM) for 24 h. Representative immunofluorescence images of cells stained with TDP-43 antibody (green), DAPI for nucleus (blue), and phalloidin for actin filament (red). TDP-43 localization is represented as TDP-43 nucleo-cytoplasm ratio (A.U, arbitrary units). Measurements were performed in at least 4 fields of view in three independent experiments using ImageJ software. Scale bar (shown in the first panel): 25 μm; applies to all images.. Statistical analysis was performed using One-way ANOVA followed by Bonferroni’s post-test. (*p < 0.05, significantly different from SH-SY5Y EA-treated cells (−), ^###^ *p* < 0.001 significantly different from SH-SY5Y positive control).

In light of the results obtained with the most promising compounds **44** and **62** regarding TTBK1 isoform-selective inhibition, BBB permeability and reduction of TDP-43 phosphorylation in cell culture models, we decided to proceed with compound **62** for further evaluation, as it exhibited the lowest TTBK1 IC_50_ value and isoform selectivity index (**44**: TTBK1 IC_50_ = 1.89 μM; SI = 0.18; **62**: TTBK1 IC_50_ = 0.89 μM; SI = 0.01; Table 1).

### X-ray Binding Mode Elucidation and Computational Studies

To investigate the relationship between our candidate **62** binding mode to TTBK1 and TTBK2 and the observed inhibitory activity (Table 1), the compound was cocrystallized with both TTBK kinase domains. The TTBK2 complex was determined at high resolution (1.5 Å), with excellent electron density for the ligand. In contrast, the TTBK1 complex was determined at medium resolution (2.7 Å) and was accordingly much less well resolved overall, with clear electron density observed only for the hinge-binding pyrrolopyrimidine moiety of the inhibitor. This difference is consistent with the crystal morphologies: the TTBK2 complex yielded thicker and more symmetrical crystals, whereas the TTBK1 complex formed large, thin plates (Figure S3). The crystallographic data confirmed that compound **62** binds to TTBK1 and TTBK2 at the ATP-binding site (PDB IDs: 31HO and 31HP, respectively). The furan moiety pointed toward the solvent region and was not resolved in the TTBK1 complex (Figure S3). As the structures provided no obvious rationale for the selectivity of **62** for TTBK1 over the highly conserved TTBK2, we performed molecular dynamics (MD) simulations to gain insights into the protein dynamics upon ligand binding. To this end, ∼700 ns MD simulations were performed starting from the corresponding crystal structures and subsequent analysis by essential dynamics (ED).

In the TTBK1 complex, compound **62** progressively shifted from its initial X-ray binding pose toward the P-loop, adopting a more deeply accommodated binding mode during the simulation. This repositioning stabilized this region in a more rigid conformation as shown in Figure 7A. In contrast, in the TTBK2 complex, compound **62** remained largely close to its initial X-ray pose throughout the simulation. The ligand stayed relatively solvent-exposed and primarily preserved its hinge interaction, without undergoing a productive rearrangement toward the P-loop. Consequently, no clear stabilization of the P-loop was observed, and this region retained higher flexibility (Figure 7B). These results suggest that hinge binding alone did not necessarily translate into a stable inhibitory binding mode and indicate that the activity of compound **62** is associated not only with its ability to bind TTBK kinases, as observed in the X-ray structures, but also with its capacity to induce and maintain P-loop stabilization over time. In fact, these findings are consistent with those obtained with our previously reported **TTBK1-IN-2** inhibitor.^29^

**Figure 7:**
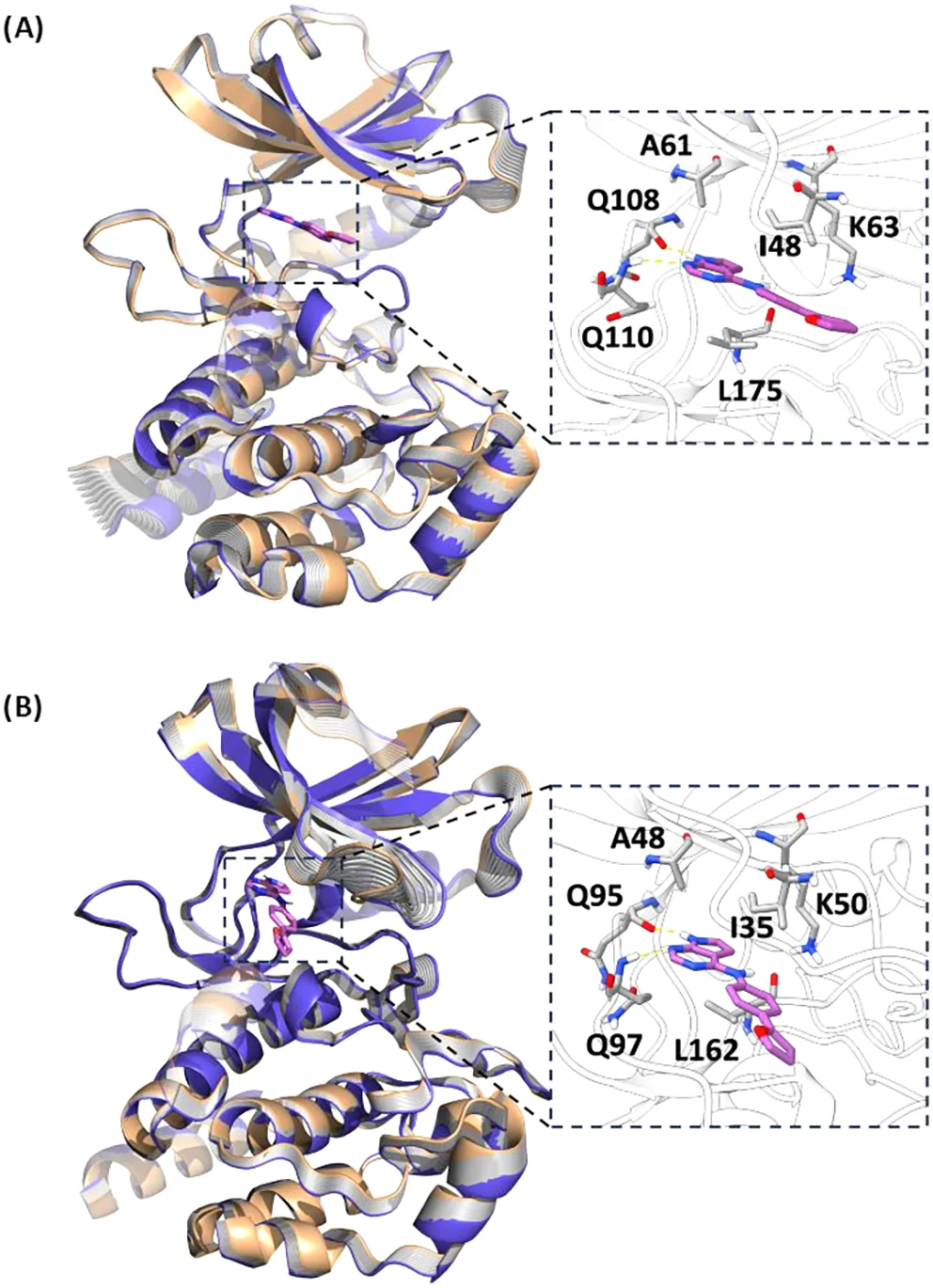
Essential dynamics (ED) analysis of the 700 ns MD simulations of the (A) TTBK1–compound **62** and (B) TTBK2-compound **62** complexes. The X-ray structures of TTBK1 and TTBK2 in complex with compound **62** (PDB IDs: 31HO and 31HP, respectively) were used as starting points for molecular modeling studies The close-up views show the ligand-binding pockets, highlighting key residues located within 5 Å of the ligand, which is represented in magenta. The initial and final conformational states visualized in the ED analysis are shown in purple and light brown, respectively, while the intermediate conformational ensembles are shown in gray. The yellow dashed lines represent hydrogen-bond interactions.

The sequence differences between TTBK1 and TTBK2 that underlie the distinct conformational responses observed during the simulations are likely not limited to residues directly involved in ligand binding and may also include residues across the kinase domain involved in long-range conformational coupling. Nevertheless, conservative substitutions near the binding site and P-loop, such as K38_TTBK1_ to R25_TTBK2_ and E50_TTBK1_ to D37_TTBK2_, could further modulate the local dynamics of this region. Although these substitutions preserve the overall charge properties of the residues, differences in side-chain length, geometry, and hydrogen-bonding or salt-bridge potential may influence local flexibility and interactions. Taken together, these broader sequence variations and local residue substitutions may modulate the dynamic environment of the binding region, thereby influencing the stability of compound **62** during the simulation. Therefore, ligand-induced P-loop stabilization suggested a possible structural and dynamic explanation for the observed differences in activity and selectivity.

### Impact of TTBK Inhibition on Ciliogenesis

Since TTBK2 plays an essential role in ciliogenesis, the involvement of TTBK1 in this cellular process has been interrogated.^24^ Consequently, one of the main objectives of this project was to study whether TTBK1 selective inhibition does not induce detrimental cellular effects associated with cilia formation. To approach this question, we used hTERT-RPE1 cells, a widely used model for cilia studies.^33–35^ First, we confirmed the endogenous expression of TTBK1 in hTERT-RPE1 cells (Figure S4) by measuring TTBK1 kinase activity using the Kinase-Glo luminescent assay and a specific phosphorylation substrate, following the protocol established for our previous enzymatic assays and detailed in the Experimental Section. Once we confirmed TTBK1 expression and activity, we treated hTERT-RPE1 cells with either our TTBK1 isoform-selective inhibitor **62** or the commercial pan-TTBK inhibitor **TTBK1-IN-1** (Figure 2). The concentrations fixed for these assays were established based on their respective TTBK1 inhibition potency, therefore selecting 4 μM for **62** and 0.4 μM for **TTBK1-IN-1**.

**TTBK1-IN-1** strongly repressed cilia formation with an estimated IC_50_ of 80 nM (Figure 8), as expected due to its potent TTBK2 inhibition (TTBK2 IC_50_ = 1.6 nM). In contrast, **62** did not interfere with cilia formation (Figure 8). These results indicate that TTBK1 is largely dispensable for this cellular process and that its selective inhibition by compound **62** did not elicit ciliogenesis-related toxicity, underscoring TTBK1 modulation as a safe strategy to address TDP-43 pathology.

**Figure 8:**
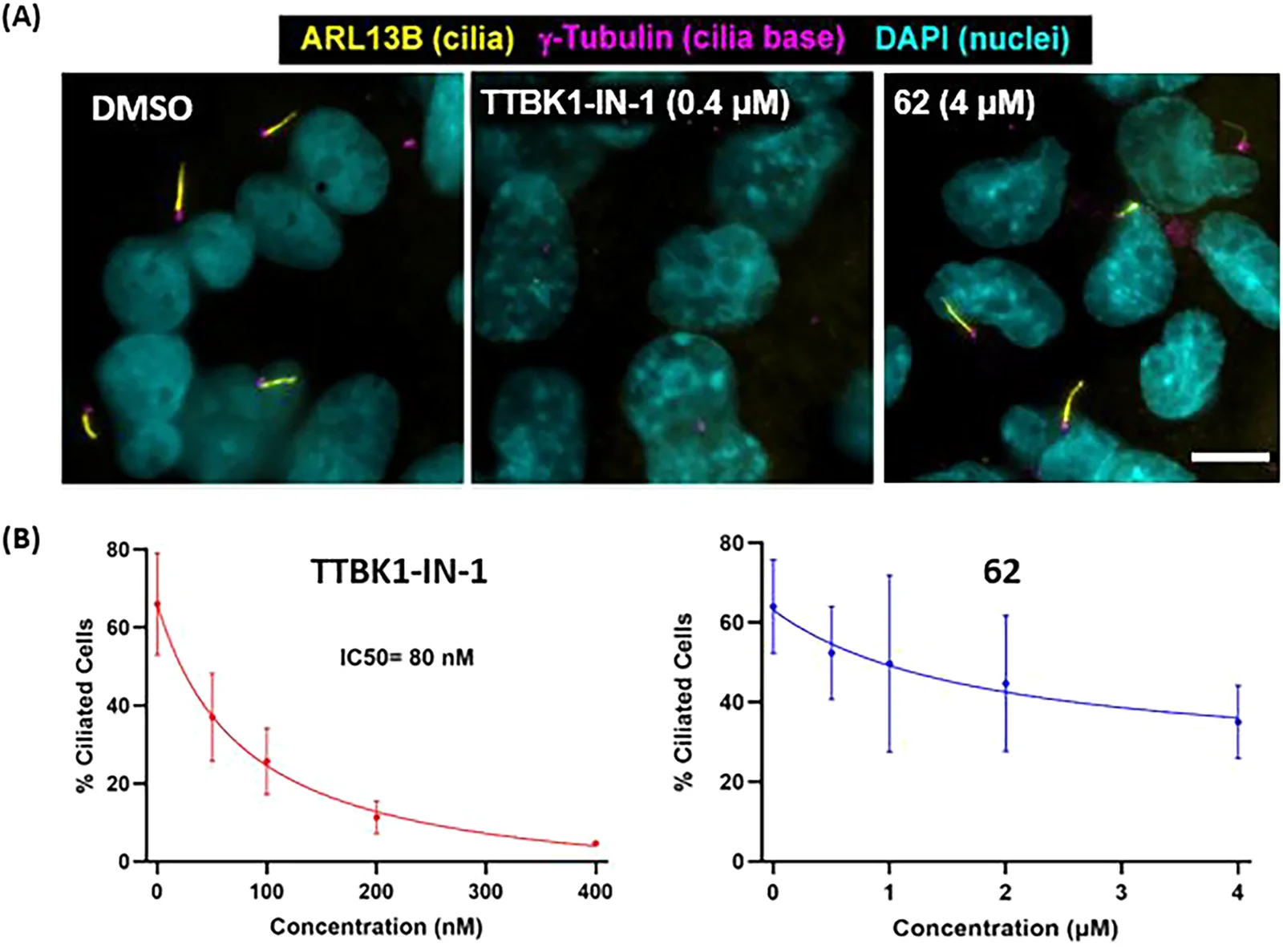
Effect of TTBK1 inhibitors on hTERT-RPE1 cilia formation. (A) hTERT-RPE1 cells were serum-starved to induce ciliogenesis in the presence of DMSO (left) as a vehicle control, **TTBK1-IN-1** (middle, 0.4 μM), or **62** (right, 4 μM). After 24 h, cells were fixed and stained for ciliary markers ARL13B, γ-tubulin, and DAPI the indicated antibodies (Table S4). Scale bar, 10 μm. (B) Percentage of ciliated cells was quantified as a function of concentration for each inhibitor. Data is shown as mean ± SEM of *n* = 3 independent experiments.

### Kinase Profiling

We have shown that compound **62** selectively inhibits TTBK1 in both enzymatic and ciliogenesis assays without perturbing TTBK2-mediated physiological functions. However, the human kinome encodes more than 500 protein kinase, and, given that compound **62** acts as an ATP-competitive inhibitor targeting a highly conserved binding pocket, a broader assessment of its selectivity was required. To this end, **62** was screened against a panel of 140 diverse kinases at a single concentration of 10 μM at the MRC Protein Phosphorylation and Ubiquitylation Unit (University of Dundee). The results, depicted in Figure 9 and Table S5, showed that compound **62** exhibited an excellent S_15_ score of 0.03, which was defined as the ratio of off-target kinases with less than 15% of remaining activity relative to the total number of kinases evaluated. In the case of **62**, only four kinase off-targets showed less than 15% residual activity: AurB, CLK2, MLK1, and MLK3. Of note, the use of full-length (FL) constructs of both TTBK1 and TTBK2 in this panel provided a robust validation of the inhibitory potency of **62** and isoform selectivity against the FL enzymes (TTBK1-FL IC_50_ = 0.72 μM; TTBK2-FL % inhibition at 10 μM = 37%).

**Figure 9:**
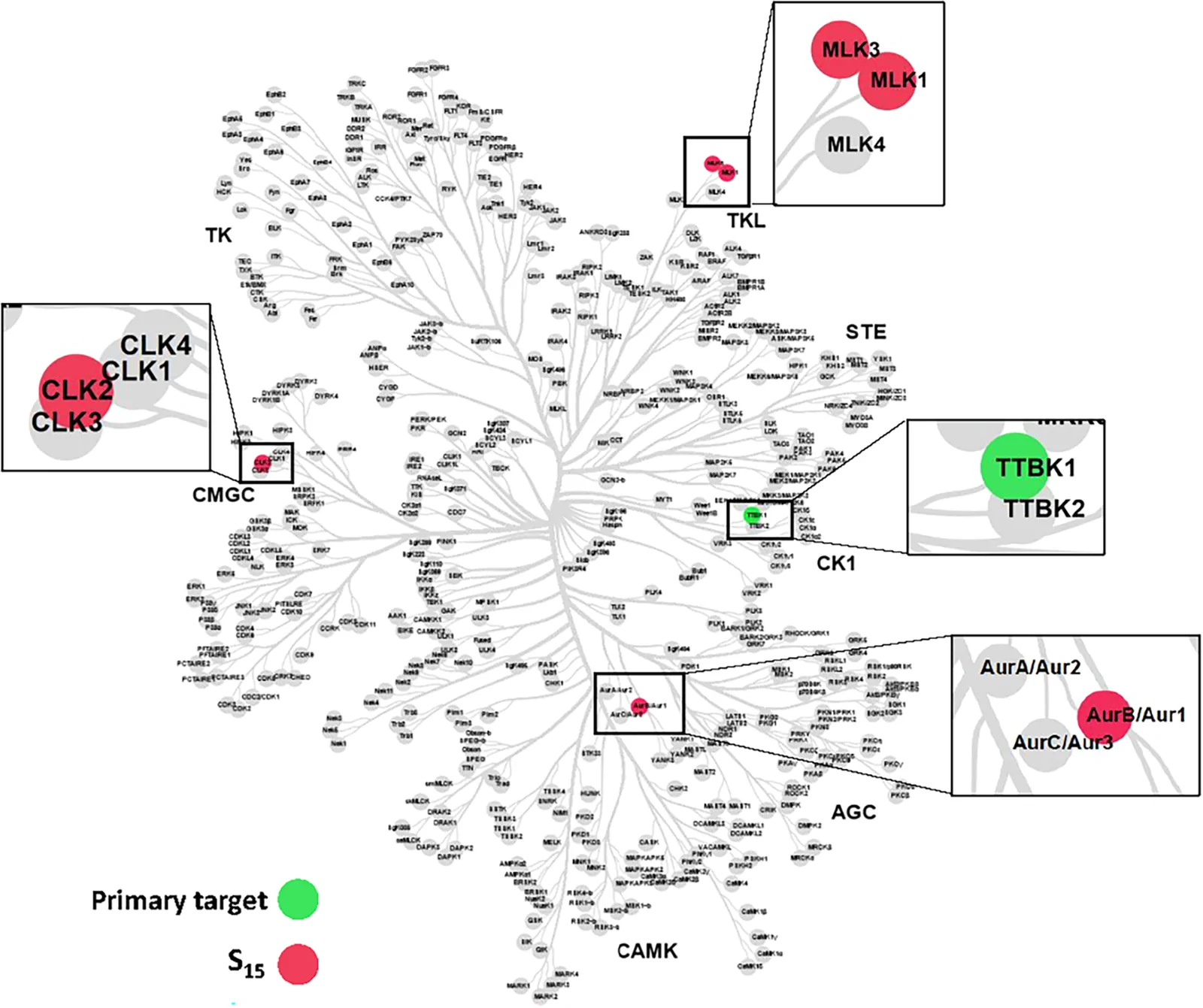
Selectivity profile of compound **62** against a panel of 140 protein kinases at a single concentration of 10 μM. Kinases with a residual activity 15% (S_15_) are shown as red nodes, and the primary target TTBK1 is shown as a green node. The complete screening results are given in Table S5. The figure was generated with CORAL.^36^

### ADME-Tox and *In Vivo* Pharmacokinetic Profiling of TTBK1 Inhibitor 62

In the context of a medicinal chemistry program, the transition from *in vitro* potency to *in vivo* efficacy requires a safe ADME-Tox profile for the compound under study. While pharmacokinetic (PK) studies characterize the systemic journey of the molecule and evaluate the candidate target-engagement; cardiotoxicity, genotoxicity, and maximum tolerated dose (MTD) evaluations can help to identify potential off-targets and DNA-damaging risks, and to establish a safety ceiling early in the pipeline.

Regarding the cardiovascular safety profile of **62**, it was assessed by evaluating its inhibitory activity against three key cardiac ion channels: Nav1.5, Cav1.2, and the human ether-a-go-go-related gene (hERG) potassium channel. These channels are critical for the initiation and propagation of the cardiac action potential, and their unintended modulation is one of the main causes of drug-induced QT interval prolongation and cardiotoxicity. Cell-based functional assays in HEK293 revealed that compound **62** exhibited no significant inhibition of hERG, Nav1.5, or Cav1.2 at the tested concentrations (Table S6). These results indicate a low risk of cardiac arrhythmia or contractile dysfunction upon treatment with **62**.

Subsequent mutagenicity tests were carried out to ensure the genomic integrity of the organism following exposure to **62**. The mutagenic potential of **62** was thereby evaluated using the Ames test at four concentrations of the compound: 50, 25, 12.5, and 6.25 μM. We found no mutagenic activity at any tested dose in *S. typhimurium* TA98 or *S. typhimurium* TA100 in the absence or presence of the S9 metabolic activation system, with revertant counts comparable to solvent controls (Tables S7–S10). It can therefore be concluded that interactions that could cause DNA-damage during treatment with **62** are unlikely.

In order to determine an appropriate administration dose for further *in vivo* efficacy studies, we evaluated the plasma pharmacokinetics and brain distribution of the selected candidate **62** in BALB/c mice. Compound **62** was administered at a single dose of 5 mg·kg^–1^ intraperitoneally (i.p.) and 10 mg·kg^–1^ orally (p.o.), exhibiting a rapid absorption with a *T*_max_ between 0.25 and 0.5 h (Table 4). These results demonstrate exceptional CNS penetration in line with previous PAMPA results (Figure 4). Despite a short systemic half-life, **62** showed preferential brain partitioning, with brain-to-plasma ratios (*K*_p_) reaching 3.84 via i.p. route. This high *K*_p_ value makes **62** an interesting lead for *in vivo* FTD-TDP-43 transgenic mouse studies, ensuring that therapeutic concentration is maintained in the CNS even with low systemic doses.

**Table 4:** *In Vivo* Pharmacokinetic Profile of Compound **62** in Plasma and Brain Following a Single Intraperitoneal (i.p.) and Oral (p.o.) Administration in Male BALB/c Mice^a^

Dose (mg/kg)	Route	Matrix	*T*_max_ (h)	*C*_max_ (ng/mL)	AUC_last_ (h·ng/mL)	*T*_1/2_ (h)	Brain *K*_p_ (C_max_)	Brain *K*_p_ (AUC_last_)
5	i.p.	Plasma	0.50	1121.53	1125.01	0.28		
10	p.o.		0.25	613.09	356.86	0.42		
5	i.p.	Brain^b^	0.50	4133.38	4323.48		3.69	3.84
10	p.o.		0.25	1092.15	984.41		1.78	2.76

a i.p. dose of 5 mg·kg^–1^ and p.o. dose of 10 mg·kg^–1^.

b Density of brain tissue was considered as 1 which is equivalent to plasma density.

In accordance with Food and Drug Administration (FDA) guidelines for the safety testing of drug metabolites, the metabolic profile of **62** was evaluated across multiple species to identify the most proper models for further preclinical toxicological studies. The results of the *in vitro* incubations of **62** with liver microsomes from humans (HLM), rats (RLM), minipigs (MPLM), and dogs (DLM) in the presence of NADPH led to the identification of six metabolites (Table S11). The comparative analysis revealed that the human metabolic profile was fully recapitulated through the combination of rat and minipig models. Consequently, these two animal species were selected for subsequent toxicological development, ensuring comprehensive coverage of human-relevant metabolic pathways.

Starting with the rat species, and with the intention of establishing the safety profile and dose-exposure relationship of TTBK1 inhibitor **62**, a 7-day repeat-dose MTD study was conducted in Sprague–Dawley rats following oral gavage administration (10, 50, and 100 mg/kg/day), using three rats per group and sex. All animals treated with inhibitor **62** survived to study termination with no observable clinical abnormalities: body weight gain, feed intake, and organ histology were comparable to control groups. Moreover, toxicokinetic analysis revealed a dose-proportional increase in systemic exposure across all dose levels in both sexes, with no evidence of compound accumulation. Consequently, the MTD for candidate **62** was determined to be at least 100 mg/kg/day, confirming a robust safety margin and a favorable toxicokinetic profile for further development.

### *In Vivo* Efficacy of TTBK1 Selective Inhibition in a TDP-43 Mouse Model

In light of the promising results shown by the selective TTBK1 inhibitor **62** in cellular models and its demonstrated safety profile, we sought to evaluate its *in vivo* efficacy in the FTD-TDP-43 transgenic mouse model developed by Tsai et al.^37^ This model recapitulated key features of FTD pathology through brain-specific overexpression of human TDP-43 driven by the CaMKIIα promoter, which ensures targeted expression in the cortex and hippocampus.

The experimental design of the study is depicted in Figure 10A consisted of a chronic daily administration of compound **62** over 45 days, initiated at presymptomatic stage with two points for cognition evaluation by the novel object recognition test (NOR), followed by biochemical and immunohistochemical analyses of brain tissue. Compound **62** significantly ameliorated cognitive deficits, as evidenced by an improved discrimination index in the NOR test at both day 15 (PND60) and day 45 (PND90) after treatment initiation (Figure 10B), supporting the efficacy of this TTBK1 inhibitor in improving cognitive function.

**Figure 10:**
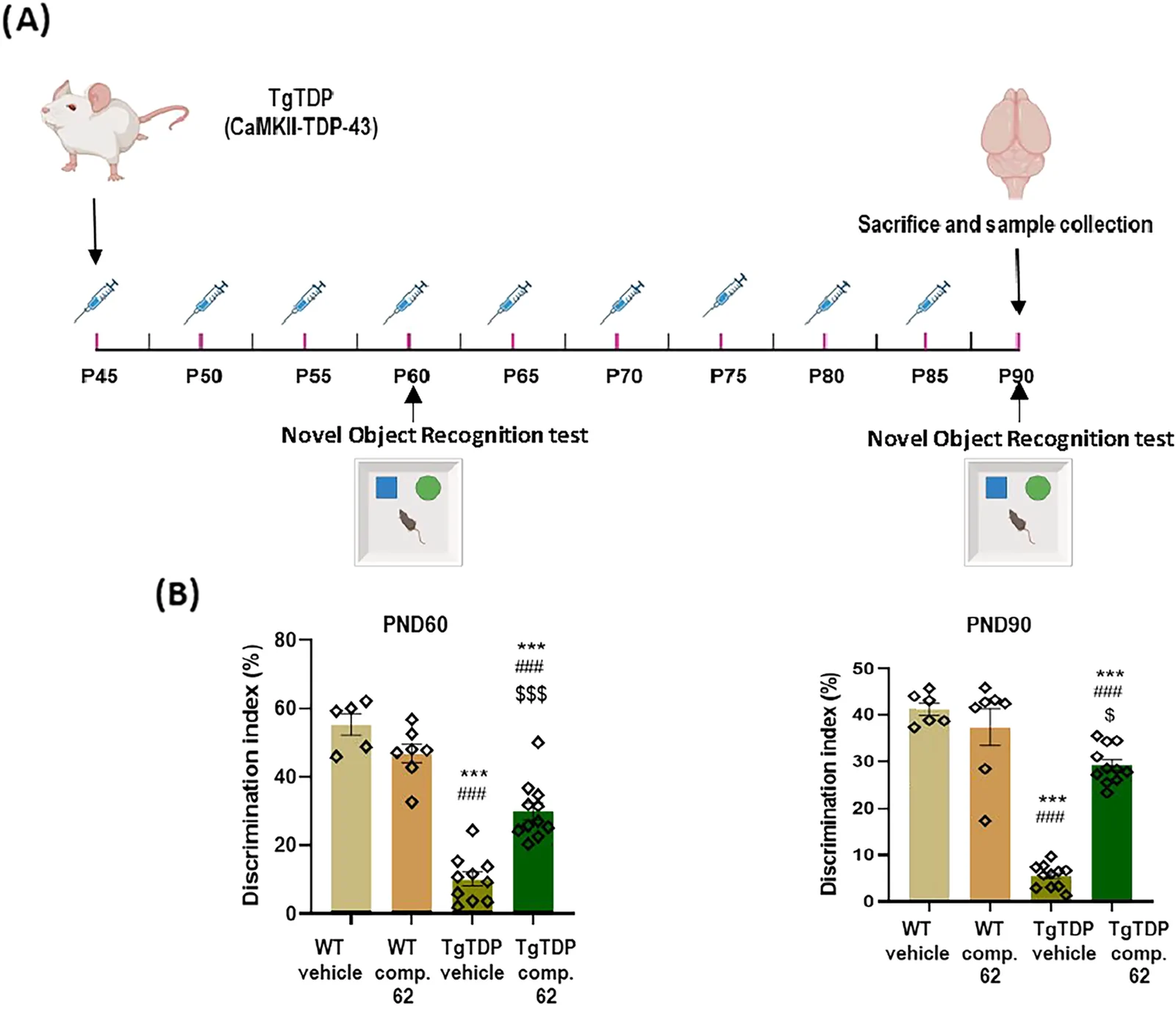
(A) Design of the *in vivo* study conducted with candidate **62** in the FTD CaMKIIα-TDP-43 mouse model; (B) impact of compound **62** on memory function measured by the NOR test in CaMKII-TDP43 mice. The discrimination index between new and old objects was recorded at PND60 and PND90. Statistical analysis was two-way ANOVA followed by Tukey’s multiple comparisons test. ***p < 0.01 vs WT-vehicle; ^###^ *p* < 0.01 vs WT **62**; ^$^ *p* < 0.05, ^$$$^ *p* < 0.01 vs CaMKII-TDP43 veh.

Furthermore, histological analysis revealed a neuroprotective effect of compound **62**, preserving neuronal density in the prefrontal cortex, as evidenced by Ctip2-positive staining, a marker of deep-layer cortical projection neurons Figure 11. This neuroprotection was associated with a marked attenuation of astroglial and microglial reactivity, as indicated by reduced S100-β and Iba1 immunoreactivity, respectively, together with a reduction in pathological TDP-43 phosphorylation (p-TDP-43) in the same brain region, assessed using a monoclonal anti-pTDP-43 (Ser409/410) antibody (Figures 11 and S5)). Overall, these findings demonstrate that the selective TTBK1 inhibitor **62** exerts a potent neuroprotective effect *in vivo*, highlighting its therapeutic potential as a pharmacological treatment for human TDP-43 proteinopathies, including disorders within the FTD-ALS spectrum.

**Figure 11:**
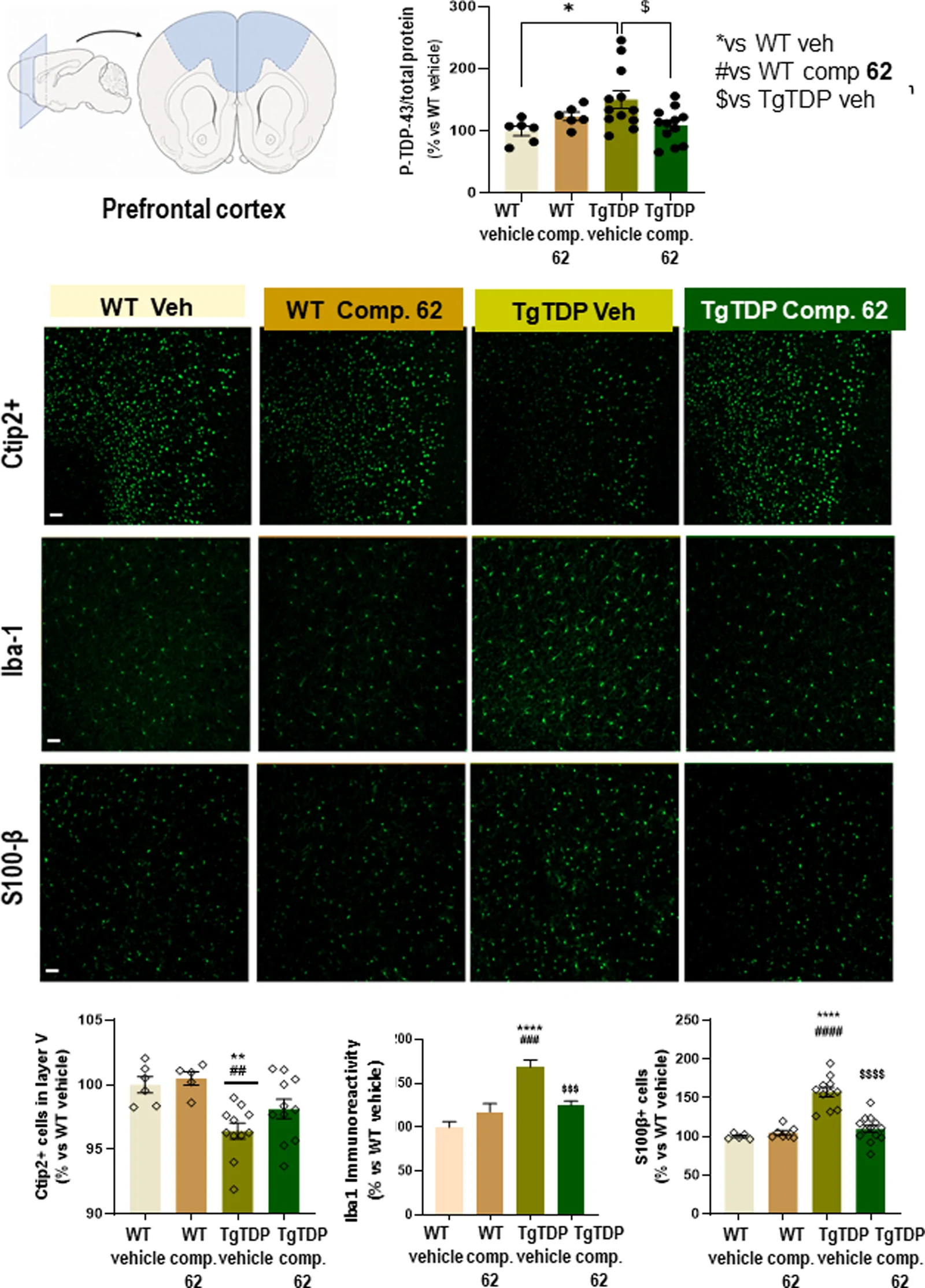
Neuroprotective effects and reduction of TDP-43 phosphorylation by TTBK1 inhibitor **62** in the CaMKII-TDP43 mouse model. (A) Quantification of phospho-TDP-43/total protein by Western Blot. (B) Representative immunofluorescence images (scale bar = 100 μm) of Ctip2, a nuclear transcription factor expressed in layer V cortical neurons of the prefrontal cortex, Iba 1 a specific microglial activation marker and S100β an astrocytic marker. (C) Quantification of Ctip2. (D) Quantification of Iba1. (E) Quantification of S100β. Data are presented as mean values ± SEM. Statistical analysis was two-way ANOVA followed by Tukey’s multiple comparisons test. *p < 0.05, **p < 0.01, ****p < 0.0001 vs WT-vehicle; ^##^ *p* < 0.01, ^###^ *p* < 0.001, ^####^ *p* < 0.0001 vs WT **62**; ^$^ *p* < 0.05, ^$$$^ *p* < 0.001, ^$$$$^ *p* < 0.0001 vs CaMKII-TDP43 veh.

### Evaluation of Lead Compound 62 in a Patient-Derived Cellular Model

Given the promising neuroprotective profile of our lead compound, we next sought to validate these findings in a highly relevant translational model of patient-derived cells. To this end, we utilized lymphoblastoid cell lines (LCLs) derived from FTD patients carrying a loss-of-function mutation in the progranulin (*GRN*) gene. Mutations in the *GRN* gene represent one of the most common causes of frontotemporal lobar degeneration with TDP-43 inclusions (FTLD-TDP), making these patient-derived cells a robust model that effectively recapitulates the endogenous TDP-43 proteinopathy, as we previously published.^38,39^

Following the established protocol, two patient-derived cell lines and two control lymphoblast cell line were treated for 24 h with compound **62** or the reference control **TTBK1-IN-2**. Western Blot analysis revealed that, under basal conditions, FTD lymphoblasts exhibited a marked increase in pathological TDP-43 phosphorylation compared to healthy controls (Figure 12). Notably, treatment with both compound **62** and **TTBK1-IN-2** effectively mitigated this pathological state by reducing phosphorylation levels in both full-length TDP-43 (43 kDa) and the characteristic cytotoxic C-terminal fragments (25 and 35 kDa). Statistical significance was reached specifically in the 25 kDa C-terminal fragment for both compounds, and in the 35 kDa fragment only for candidate **62** (Figure 12). Therefore, this inhibitory effect was more pronounced for **62** than for the reference **TTBK1-IN-2**, further supporting the higher potency of our novel lead candidate in attenuating the pathogenic and truncated variants of TDP-43 in this human translational model.

**Figure 12:**
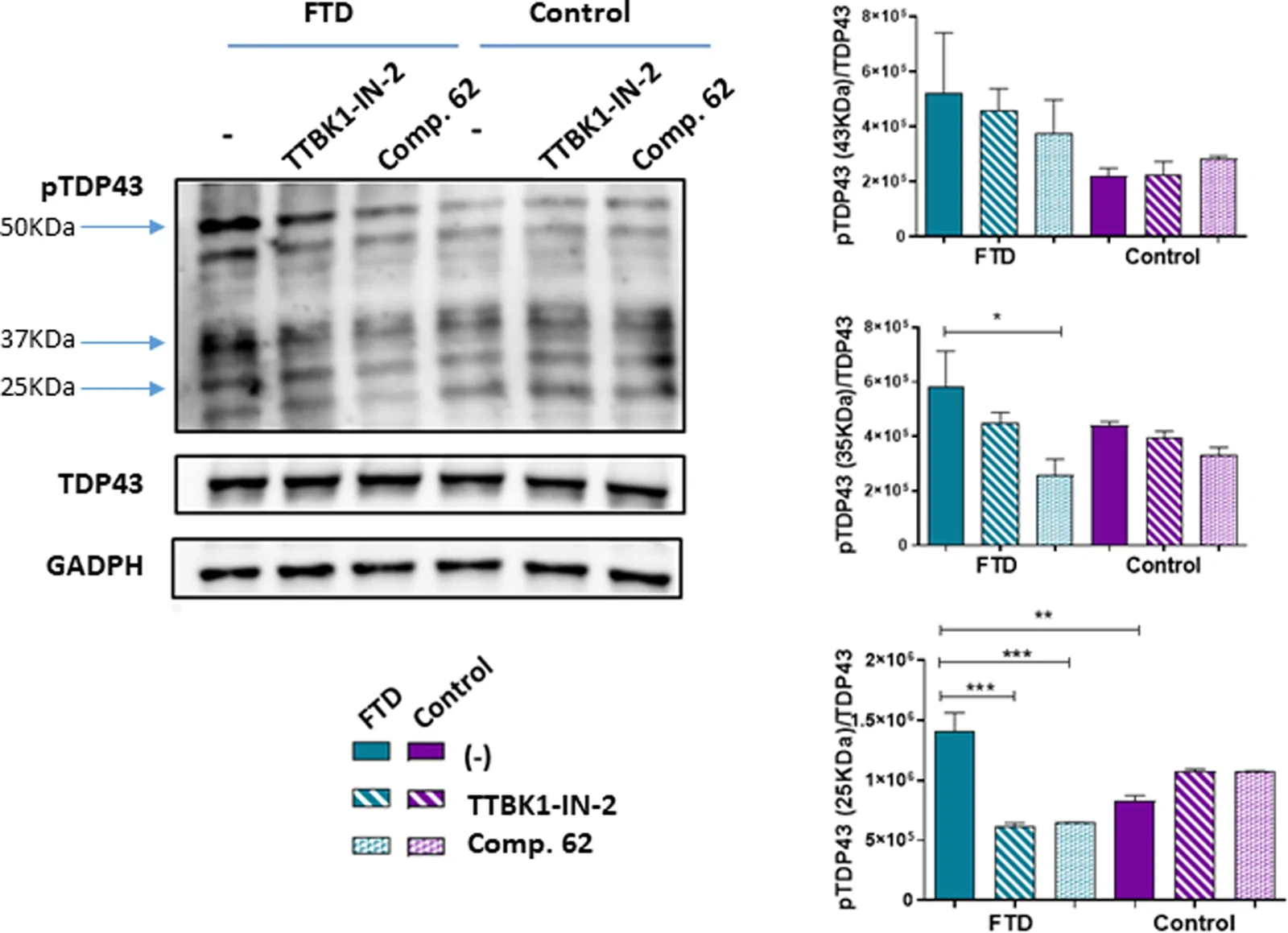
Effect of TTBK1 inhibitors on TDP-43 phosphorylation status in lymphoblasts from FTD patients carrying a progranulin (*GRN*) mutation and a healthy subjects. Lymphoblasts were treated with compound **62** (5 μM) or the reference compound **TTBK1-IN-2** (5 μM) for 24 h. Representative immunoblots and quantification of phosphorylated TDP-43 at Ser409/410 (full-length, 35 kDa, and 25 kDa fragments) are shown. Data are expressed as mean ± SEM of *n* = 3 independent experiments. Statistical analysis was performed using one-way ANOVA followed by Bonferroni’s posthoc test (*p < 0.05, **p < 0.01, ***p < 0.001).

To further characterize the therapeutic impact of compound **62**, we assessed the subcellular localization of TDP-43 by immunofluorescence microscopy. In line with the observed phosphorylation patterns, untreated FTD lymphoblasts displayed a significant mislocalization of TDP-43 from the nucleus to the cytoplasm (Figure 13). However, treatment with our selected inhibitors led to a notable restoration of nuclear TDP-43 localization, reaching statistical significance in lymphoblasts treated with compound **62** (Figure 13). These results corroborate our previous findings in neuronal cell lines and reinforce the potential of these TTBK1 inhibitors to rescue TDP-43 homeostasis in a human patient-derived context.

**Figure 13:**
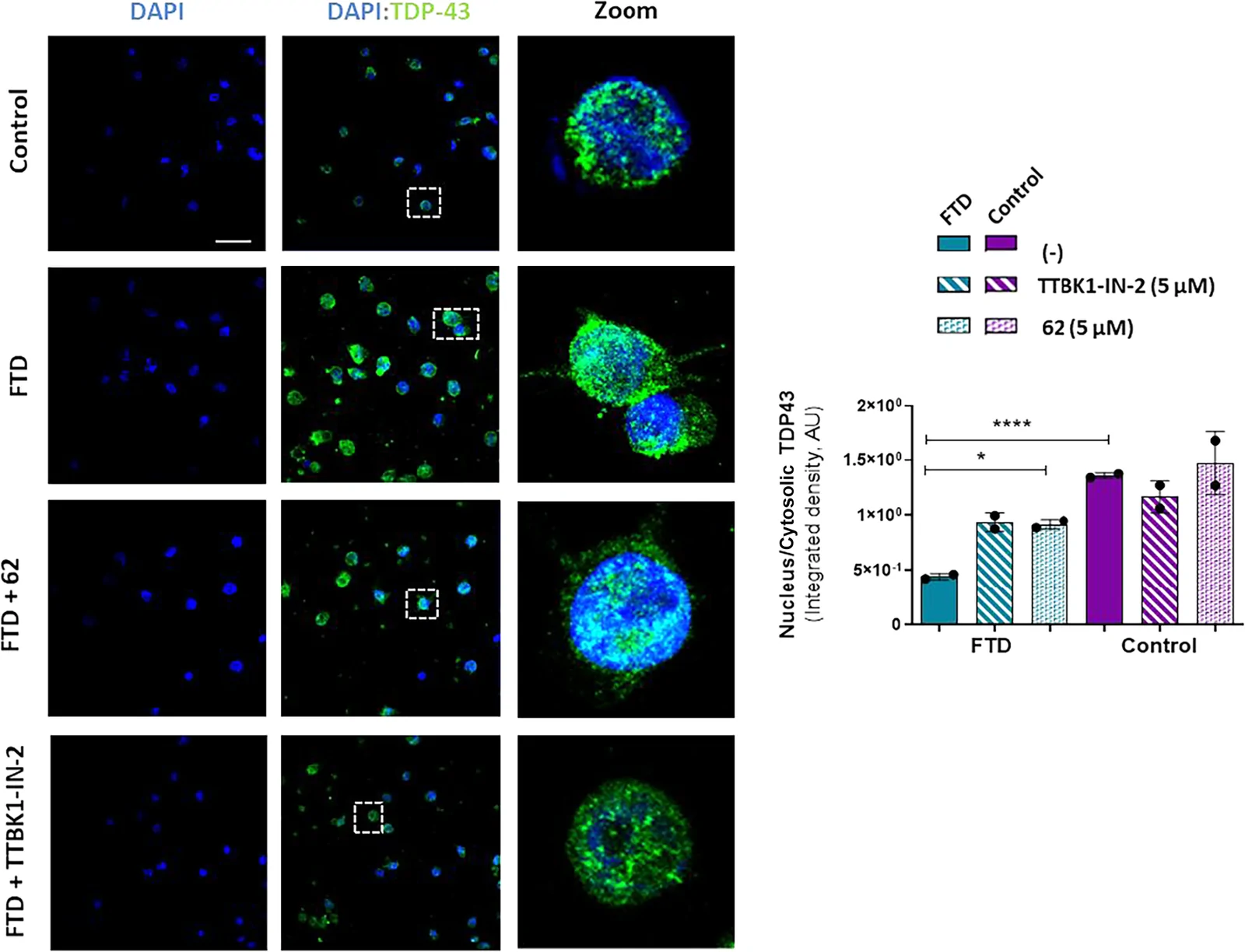
Effect of TTBK1 inhibitors on TDP-43 subcellular localization in lymphoblasts from FTD patients carrying a progranulin (*GRN*) mutation and a healthy subject. Representative immunofluorescence images of cells stained with a TDP-43 antibody (green) and DAPI (blue) are shown (Scale bar: 20 μm). TDP-43 immunofluorescence intensity (A.U., arbitrary units) was measured in at least 4 fields of view using ImageJ software. TDP-43 localization is expressed as the nuclear-cytoplasmic ratio. Statistical analysis was performed using one-way ANOVA followed by Bonferroni’s posthoc test (*p < 0.05, ****p < 0.0001).

## Conclusions

TDP-43 proteinopathies are gaining increasing relevance in the therapeutic field due to the lack of effective treatments and the severity of the associated diseases, including neuromuscular dysfunction in ALS and dementia in AD, LATE, and FTD. Small molecules capable of modulating TDP-43 pathology and restoring their physiological homeostasis may represent promising drug candidates for these disorders in the near future. Selective brain-permeable TTBK1 inhibitors have already been proposed as a potential therapeutic strategy for ALS,^29^ and the data presented in this manuscript demonstrate their efficacy also in FTD-TDP therapy.

Through a medicinal chemistry program, selective and brain-permeable TTBK1 inhibitors were developed. Target-based optimization has been challenging because the crystal structure of the same ligand bound to both isoenzymes, TTBK1 and TTBK2, had essentially identical binding modes, despite the selectivity observed in biochemical assays. However, molecular dynamics (MD) simulations suggested that P-loop stabilization may play a critical role in TTBK inhibition, favoring the binding of compound **62** to TTBK1, but not TTBK2. This behavior was probably driven by nonconserved residues within the P-loop.

Following a series of assays, including BBB penetration and phenotypic evaluation of TDP-43 recovery, the TTBK1 inhibitor **62** was selected for further development. This compound did not interfere with the ciliogenesis process in cellular models and exhibited an *in vivo* brain-to-plasma ratio of 3:1, together with a wide therapeutic window, showing a maximum tolerated dose >100 μM, whereas the effective dose is 5 mg/kg in the CaMKII-TDP43 transgenic model.

Chronic administration of compound **62** restored cognitive performance to levels comparable to those of wild-type animals. This effect mirrors the neuronal preservation observed in the prefrontal cortex and may be associated with the reduction in astrogliosis and microgliosis activation, together with decreased TDP-43 phosphorylation in the same brain region. In addition, the safety profile of this compound, particularly regarding the Ames test and ion channel inhibition, supports its potential as a promising candidate for further development as a selective TTBK1 inhibitor.

Finally, it is also worth mentioning that compound **62** was able to restore the physiological homeostasis of TDP-43 in lymphoblastoid cell lines derived from FTD patients carrying *GRN* mutations, suggesting promising translational potential. Only future clinical trials can confirm the therapeutic potential of TTBK1 inhibitors for the treatment of FTD and other dementias.

## Experimental Section

### Chemistry

Analytical grade solvents purchased from Sigma-Aldrich were used for all reactions. Commercially available reagents were used without further purification. Argon was used to carry out the reactions in an inert atmosphere. Microwave-assisted reactions were carried out using an Initiator device (Biotage). Experiments were performed with the temperature control mode in sealed microwave process vials. The temperature was measured with an IR sensor on the outside of the reaction vessel. Precoated aluminum foils (TLC plates GF254, Merck) were used for thin layer chromatography (TLC) to monitor all reactions. Crude reaction products were purified by flash column chromatography using the indicated solvent as eluent using either silica gel (E. Merck, grade 60, particle size 0.040–0.063 mm, 230–240 mesh ASTM) or the IsoleraOne flash purification system from Biotage. ^1^H and ^13^C NMR (nuclear magnetic resonance) spectra were recorded on a Bruker AV300 MHz (^1^H NMR, 300 MHz; ^13^C NMR, 75 MHz) or on a 500 MHz instrument (^1^H NMR, 500 MHz; ^13^C NMR, 125 MHz) from the NMR unit of Research Assistance Centers from Complutense University of Madrid. Chemical shifts are reported in δ values (ppm) and were calculated taking the reference of the appropriated deuterated solvents. Coupling constants (*J*) are expressed in Hz. The abbreviations used are s (singlet), d (doublet), t (triplet), q (quartet), and m (multiplet). Coupling constants (*J*) are expressed in Hz. Melting points (mp) were determined in a Büchi Melting Point M-560 apparatus. High-resolution mass spectrometry (HRMS-ESI) was performed in an Agilent 6546 mass spectrometer with an ESI/APCI ionization source and quadrupole/time-of-flight (QTOF), coupled to an Agilent 1260 Infinity II liquid chromatograph, at the Mass Spectrometry Service of the Institute of General Organic Chemistry (IQOG-CSIC). HPLC–MS (high-pressure liquid chromatography coupled to mass spectrometry) analysis was carried out on an Agilent 1260 Infinity II HPLC instrument coupled with an Agilent MSD XT quadrupole and equipped with a ZORBAX SB-C18 column (3.5 μM, 50 mm × 4.6 mm) using scan positive electrospray and H_2_O (0.1% H–COOH)/MeCN (0.09% H–COOH) as eluent at a flow rate of 0.8 mL/min and 23 °C, with an injection volume of 5 μL and using the following gradient: initial MeCN concentration of 5%, linear increase to 100% over 3 min, held at 100% for 1.45 min, followed by linear decrease back to 5% over 0.55 min. All the evaluated compounds are >95% pure by HPLC analysis.

The synthesis of the intermediates **2–42** and **85–87** is described in the Supporting Information.

### General Procedure for the Synthesis of Derivatives 1, 43–84

One Equiv. of 4-chloro-7*H*-pyrrolo­[2,3-*d*]­pyrimidine; 1 equiv of the corresponding aniline; and 0.1 equiv of InCl_3_ were dissolved in anhydrous MeCN or THF as indicated in each case. The mixture was stirred under MW at 120 °C until the reaction was complete. Afterward, the solvent was evaporated under reduced pressure and the crude residue was dissolved in CH_2_Cl_2_, neutralized using NaHCO_3_ and washed with brine. The organic layer was dried over MgSO_4_ and concentrated under high vacuum. The corresponding crude was purified by flash chromatography using CH_2_Cl_2_/MeOH (32:1) as eluent.

#### *N*-(4′-Methoxy-(1,1′-biphenyl)-4-yl)-7*H*-pyrrolo­[2,3-*d*]­pyrimidin-4-amine (**1**)

The title compound was obtained by reaction of 4-chloro-7*H*-pyrrolo­[2,3-*d*]­pyrimidine (1.00 mmol, 153 mg), 4′-methoxy-(1,1′-biphenyl)-4-amine (1.00 mmol, 200 mg), InCl_3_ (0.1 mmol, 22 mg), and anhydrous MeCN (4 mL). Yield: 30%. White solid. Mp 260–261 °C. ^1^H NMR (300 MHz, DMSO-*d*_6_) δ 11.76 (s, 1H), 9.37 (s, 1H), 8.30 (s, 1H), 8.00–7.95 (m, 2H), 7.70–7.55 (m, 4H), 7.24 (dd, *J* = 3.5, 2.3 Hz, 1H), 7.01 (d, *J* = 8.8 Hz, 1H), 6.82 (dd, *J* = 3.5, 1.9 Hz, 1H), 3.79 (s, 3H). ^13^C NMR (75 MHz, DMSO-*d*_6_) δ 158.4, 153.5, 150.9, 150.8, 139.3, 133.4, 132.4, 127.2 (2C), 126.1 (2C), 122.2, 120.5 (2C), 114.3 (2C), 103.7, 98.8, 55.1. HRMS (ESI): calc. for C_19_H_16_N_4_O [M + H]^+^ 317.1397; found: 317.1396. HPLC-MS (M + H)^+^ = 317.1, *R*_t_ = 2.76 min (98%).

#### *N*-((1,1′-Biphenyl)-4-yl)-7*H*-pyrrolo­[2,3-*d*]­pyrimidin-4-amine (**43**)

The title compound was obtained by reaction of 4-chloro-7*H*-pyrrolo­[2,3-*d*]­pyrimidine (0.55 mmol, 84 mg), (1,1′-biphenyl)-4-amine (**2**) (0.55 mmol, 93 mg), InCl_3_ (0.05 mmol, 12 mg) and anhydrous MeCN (4 mL). Yield: 50%. Beige solid. Mp 279–280 °C. ^1^H NMR (300 MHz, DMSO-*d*_6_) δ 11.80 (s, 1H), 9.44 (s, 1H), 8.32 (s, 1H), 8.02 (d, *J* = 8.7 Hz, 2H), 7.76–7.60 (m, 4H), 7.45 (t, *J* = 7.6 Hz, 2H), 7.36–7.28 (m, 1H), 7.26 (dd, *J* = 3.5, 2.3 Hz, 1H), 6.83 (dd, *J* = 3.5, 1.9 Hz, 1H). ^13^C NMR (75 MHz, DMSO-*d*_6_) δ 153.4, 150.8, 150.7, 140.3, 139.9, 133.6, 128.9 (2C), 126.8, 126.7 (2C), 126.1 (2C), 122.3, 120.5 (2C), 103.8, 98.8. HRMS (ESI): calc. for C_18_H_14_N_4_ [M + H]^+^ 287.1291; found: 287.1291. HPLC-MS (M + H)^+^ = 287.2, *R*_t_ = 2.82 min (95%).

#### 4′-((7*H*-Pyrrolo­[2,3-*d*]­pyrimidin-4-yl)­amino)-(1,1′-biphenyl)-2-ol (**44**)

The title compound was obtained by reaction of 4-chloro-7*H*-pyrrolo­[2,3-*d*]­pyrimidine (0.41 mmol, 63 mg), 4′-amino-(1,1′-biphenyl)-2-ol (**3**) (0.41 mmol, 76 mg), InCl_3_ (0.04 mmol, 9 mg) and anhydrous MeCN (4 mL). Yield: 55%. Beige solid. Mp 260–261 °C. ^1^H NMR (300 MHz, DMSO-*d*_6_) δ 11.75 (s, 1H), 9.46 (s, 1H), 9.35 (s, 1H), 8.28 (s, 1H), 7.95–7.87 (m, 2H), 7.57–7.49 (m, 2H), 7.30–7.22 (m, 2H), 7.13 (td, *J* = 7.6, 1.8 Hz, 1H), 6.96–6.78 (m, 3H). ^13^C NMR (75 MHz, DMSO-*d*_6_) δ 154.3, 153.5, 150.8, 150.7, 138.9, 132.2, 130.1, 129.1 (2C), 128.0, 127.6, 122.1, 119.7 (2C), 119.4, 116.0, 103.7, 98.8. HRMS (ESI): calc. for C_18_H_14_N_4_O [M + H]^+^ 303.1240; found: 303.1241. HPLC-MS (M + H)^+^ = 303.1, *R*_t_ = 2.46 min (96%).

#### 4′-((7*H*-Pyrrolo­[2,3-*d*]­pyrimidin-4-yl)­amino)-(1,1′-biphenyl)-3-ol (**45**)

The title compound was obtained by reaction of 4-chloro-7*H*-pyrrolo­[2,3-*d*]­pyrimidine (0.58 mmol, 89 mg), 4′-amino-(1,1′-biphenyl)-3-ol (**4**) (0.58 mmol, 107 mg), InCl_3_ (0.06 mmol, 13 mg) and anhydrous MeCN (4 mL). Yield: 30%. White solid. Mp 243–244 °C. ^1^H NMR (300 MHz, DMSO-*d*_6_) δ 12.06 (s, 1H), 9.95 (s, 1H), 9.55 (s, 1H), 8.32 (s, 1H), 7.92 (d, *J* = 8.7 Hz, 2H), 7.63 (d, *J* = 8.7 Hz, 2H), 7.31 (m, 1H), 7.24 (t, *J* = 7.8 Hz, 1H), 7.12–7.02 (m, 2H), 6.88 (m, 1H), 6.75 (ddd, *J* = 8.0, 2.4, 1.0 Hz, 1H). ^13^C NMR (75 MHz, DMSO-*d*_6_) δ 157.9, 152.5, 149.7, 148.8, 141.1, 138.7, 135.0, 130.0, 126.9 (2C), 123.0, 121.6 (2C), 117.1, 114.1, 113.1, 103.5, 99.7. HRMS (ESI): calc. for C_18_H_14_N_4_O [M + H]^+^ 303.1240; found: 303.1242. HPLC-MS (M + H)^+^ = 303.2, *R*_t_ = 2.43 min (99%).

#### 4′-((7*H*-Pyrrolo­[2,3-*d*]­pyrimidin-4-yl)­amino)-(1,1′-biphenyl)-4-ol (**46**)

The title compound was obtained by reaction of 4-chloro-7*H*-pyrrolo­[2,3-*d*]­pyrimidine (0.32 mmol, 50 mg), 4′-amino-(1,1′-biphenyl)-4-ol (**5**) (0.32 mmol, 60 mg), InCl_3_ (0.03 mmol, 7 mg) and anhydrous MeCN (2 mL). Yield: 28%. White solid. Mp 279–280 °C. ^1^H NMR (300 MHz, DMSO-*d*_6_) δ 11.74 (s, 1H), 9.45 (s, 1H), 9.33 (s, 1H), 8.28 (s, 1H), 7.94 (d, *J* = 8.7 Hz, 2H), 7.55 (d, *J* = 8.7 Hz, 2H), 7.48 (d, *J* = 8.6 Hz, 2H), 7.23 (dd, *J* = 3.5, 2.3 Hz, 1H), 6.83 (d, *J* = 8.7 Hz, 2H), 6.80 (dd, *J* = 3.5, 1.9 Hz, 1H). ^13^C NMR (75 MHz, DMSO-*d*_6_) δ 156.6, 153.4, 150.8, 150.7, 139.0, 133.8, 130.8, 127.2 (2C), 125.9 (2C), 122.1, 120.5 (2C), 115.7 (2C), 103.7, 98.8. HRMS (ESI): calc. for C_18_H_14_N_4_O [M + H]^+^ 303.1240; found: 303.1243. HPLC-MS (M + H)^+^ = 303.1, *R*_t_ = 2.37 min (98%).

#### *N*-(2′-Methoxy-(1,1′-biphenyl)-4-yl)-7*H*-pyrrolo­[2,3-*d*]­pyrimidin-4-amine (**47**)

The title compound was obtained by reaction of 4-chloro-7*H*-pyrrolo­[2,3-*d*]­pyrimidine (0.45 mmol, 68 mg), 2′-methoxy-(1,1′-biphenyl)-4-amine (**6**) (0.45 mmol, 90 mg), InCl_3_ (0.04 mmol, 10 mg) and anhydrous MeCN (3 mL). Yield: 49%. White solid. Mp 202–203 °C. ^1^H NMR (300 MHz, DMSO-*d*_6_) δ 11.94 (s, 1H), 9.70 (s, 1H), 8.31 (s, 1H), 7.98–7.81 (m, 2H), 7.58–7.44 (m, 2H), 7.31 (m, 3H), 7.10 (dd, *J* = 8.7, 1.1 Hz, 1H), 7.03 (td, *J* = 7.4, 1.1 Hz, 1H), 6.87 (dd, *J* = 3.5, 1.7 Hz, 1H), 3.78 (s, 3H). ^13^C NMR (75 MHz, DMSO-*d*_6_) δ 156.2, 152.9, 150.2, 149.6, 138.5, 132.5, 130.2, 129.5, 129.5 (2C), 128.5, 122.6, 120.8, 120.4 (2C), 111.7, 103.5, 99.4, 55.5. HRMS (ESI): calc. for C_19_H_16_N_4_O [M + H]^+^ 317.1397; found: 317.1396. HPLC-MS (M + H)^+^ = 317.2, *R*_t_ = 2.81 min (99%).

#### *N*-(3′-Methoxy-(1,1′-biphenyl)-4-yl)-7*H*-pyrrolo­[2,3-*d*]­pyrimidin-4-amine (**48**)

The title compound was obtained by reaction of 4-chloro-7*H*-pyrrolo­[2,3-*d*]­pyrimidine (0.68 mmol, 104 mg), 3′-methoxy-(1,1′-biphenyl)-4-amine (**7**) (0.68 mmol, 135 mg), InCl_3_ (0.07 mmol, 15 mg) and anhydrous MeCN (4 mL). Yield: 62%. Gray solid. Mp 207–208 °C. ^1^H NMR (300 MHz, DMSO-*d*_6_) δ 11.78 (s, 1H), 9.42 (s, 1H), 8.31 (s, 1H), 8.01 (d, *J* = 8.7 Hz, 2H), 7.66 (d, *J* = 8.8 Hz, 2H), 7.36 (t, *J* = 7.9 Hz, 1H), 7.30–7.15 (m, 3H), 6.89 (dd, *J* = 8.2, 2.5 Hz, 1H), 6.83 (dd, *J* = 8.5, 2.5 Hz, 1H), 3.83 (s, 3H). ^13^C NMR (75 MHz, DMSO-*d*_6_) δ 159.7, 153.4, 150.9, 150.7, 141.5, 140.1, 133.4, 129.9, 126.8 (2C), 122.3, 120.3 (2C), 118.5, 112.3, 111.6, 103.8, 98.8, 55.1. HRMS (ESI): calc. for C_19_H_16_N_4_O [M + H]^+^ 317.1397; found: 317.1399. HPLC-MS (M + H)^+^ = 317.1, *R*_t_ = 2.83 min (98%).

#### 4′-((7*H*-Pyrrolo­[2,3-*d*]­pyrimidin-4-yl)­amino)-(1,1′-biphenyl)-4-yl Acetate (**49**)

The title compound was obtained by reaction of 4-chloro-7*H*-pyrrolo­[2,3-*d*]­pyrimidine (0.33 mmol, 51 mg), 4′-amino-(1,1′-biphenyl)-4-yl acetate (**8**) (0.33 mmol, 70 mg), InCl_3_ (0.03 mmol, 7 mg) and anhydrous MeCN (3 mL). Yield: 53%. Gray solid. Mp 281–282 °C. ^1^H NMR (300 MHz, DMSO-*d*_6_) δ 11.78 (s, 1H), 9.41 (s, 1H), 8.31 (s, 1H), 8.02 (d, *J* = 8.7 Hz, 2H), 7.70 (d, *J* = 8.7 Hz, 2H), 7.66 (d, *J* = 8.8 Hz, 2H), 7.26 (dd, *J* = 3.5, 2.3 Hz, 1H), 7.20 (d, *J* = 8.7 Hz, 2H), 6.83 (dd, *J* = 3.5, 1.9 Hz, 1H), 2.29 (s, 3H). ^13^C NMR (75 MHz, DMSO-*d*_6_) δ 169.3, 153.4, 150.9, 150.7, 149.5, 140.0, 137.6, 132.7, 127.1 (2C), 126.7 (2C), 122.3, 122.2 (2C), 120.4 (2C), 103.8, 98.8, 20.9. HRMS (ESI): calc. for C_20_H_16_N_4_O_2_ [M + H]^+^ 345.1346; found: 345.1346. HPLC-MS (M + H)^+^ = 345.2, *R*_t_ = 2.72 min (98%).

#### *N*-(4′-Ethoxy-(1,1′-biphenyl)-4-yl)-7*H*-pyrrolo­[2,3-*d*]­pyrimidin-4-amine (**50**)

The title compound was obtained by reaction of 4-chloro-7*H*-pyrrolo­[2,3-*d*]­pyrimidine (0.65 mmol, 99 mg), 4′-ethoxy-(1,1′-biphenyl)-4-amine (**9**) (0.65 mmol, 140 mg), InCl_3_ (0.06 mmol, 13 mg) and anhydrous MeCN (4 mL). Yield: 17%. Gray solid. Mp 289–290 °C. ^1^H NMR (300 MHz, DMSO-*d*_6_) δ 11.75 (s, 1H), 9.36 (s, 1H), 8.29 (s, 1H), 7.96 (d, *J* = 8.7 Hz, 2H), 7.64–7.52 (m, 4H), 7.24 (dd, *J* = 3.5, 2.3 Hz, 1H), 6.99 (d, *J* = 8.8 Hz, 2H), 6.81 (dd, *J* = 3.5, 1.9 Hz, 1H), 4.06 (q, *J* = 6.9 Hz, 2H), 1.34 (t, *J* = 7.0 Hz, 3H). ^13^C NMR (75 MHz, DMSO-*d*_6_) δ 157.7, 153.4, 150.9, 150.8, 139.3, 133.4, 132.3, 127.2 (2C), 126.1 (2C), 122.2, 120.5 (2C), 114.80 (2C), 103.7, 98.8, 63.1, 14.7. HRMS (ESI): calc. for C_20_H_18_N_4_O [M + H]^+^ 331.1553; found: 331.1557. HPLC-MS (M + H)^+^ = 331.1, *R*_t_ = 2.96 min (99%).

#### *N*-(2′-Chloro-(1,1′-biphenyl)-4-yl)-7*H*-pyrrolo­[2,3-*d*]­pyrimidin-4-amine (**51**)

The title compound was obtained by reaction of 4-chloro-7*H*-pyrrolo­[2,3-*d*]­pyrimidine (0.59 mmol, 90 mg), 2′-chloro-(1,1′-biphenyl)-4-amine (**10**) (0.59 mmol, 120 mg), InCl_3_ (0.06 mmol, 13 mg) and anhydrous THF (3 mL). Yield: 21%. White solid. Mp 231–232 °C. ^1^H NMR (300 MHz, DMSO-*d*_6_) δ 11.77 (s, 1H), 9.42 (s, 1H), 8.31 (s, 1H), 8.01 (d, *J* = 8.7 Hz, 2H), 7.56 (dd, *J* = 7.9, 1.3 Hz, 1H), 7.46–7.35 (m, 5H), 7.26 (dd, *J* = 3.5, 2.3 Hz, 1H), 6.83 (dd, *J* = 3.5, 1.9 Hz, 1H). ^13^C NMR (75 MHz, DMSO-*d*_6_) δ 153.4, 150.9, 150.6, 140.0, 139.6, 132.1, 131.4, 131.3, 129.8, 129.3 (2C), 128.7, 127.4, 122.2, 119.6 (2C), 103.8, 98.7. HRMS (ESI): calc. for C_18_H_13_ClN_4_ [M + H]^+^ 321.0902; found: 321.0904. HPLC-MS (M + H)^+^ = 321.1, *R*_t_ = 3.06 min (99%).

#### *N*-(3′-Chloro-(1,1′-biphenyl)-4-yl)-7*H*-pyrrolo­[2,3-*d*]­pyrimidin-4-amine (**52**)

The title compound was obtained by reaction of 4-chloro-7*H*-pyrrolo­[2,3-*d*]­pyrimidine (0.20 mmol, 29 mg), 3′-chloro-(1,1′-biphenyl)-4-amine (**11**) (0.20 mmol, 40 mg), InCl_3_ (0.02 mmol, 5 mg) and anhydrous THF (2 mL). Yield: 32%. White solid. Mp 269–270 °C. ^1^H NMR (300 MHz, DMSO-*d*_6_) δ 11.79 (s, 1H), 9.44 (s, 1H), 8.32 (s, 1H), 8.04 (d, *J* = 8.8 Hz, 2H), 7.73 (t, *J* = 1.9 Hz, 1H), 7.70 (d, *J* = 8.7 Hz, 2H), 7.65 (dt, *J* = 7.8, 1.4 Hz, 1H), 7.47 (t, *J* = 7.9 Hz, 1H), 7.39–7.36 (m, 1H), 7.26 (dd, *J* = 3.5, 2.3 Hz, 1H), 6.83 (dd, *J* = 3.5, 2.0 Hz, 1H). ^13^C NMR (75 MHz, DMSO-*d*_6_) δ 153.3, 150.9, 150.7, 142.1, 140.7, 133.7, 131.7, 130.7, 126.9 (2C), 126.5, 125.7, 124.7, 122.4, 120.2 (2C), 103.9, 98.7. HRMS (ESI): calc. for C_18_H_13_ClN_4_ [M + H]^+^ 321.0902; found: 321.0903. HPLC-MS (M + H)^+^ = 321.1, *R*_t_ = 3.18 min (95%).

#### *N*-(4′-Chloro-(1,1′-biphenyl)-4-yl)-7*H*-pyrrolo­[2,3-*d*]­pyrimidin-4-amine (**53**)

The title compound was obtained by reaction of 4-chloro-7*H*-pyrrolo­[2,3-*d*]­pyrimidine (0.14 mmol, 22 mg), 4′-chloro-(1,1′-biphenyl)-4-amine (**12**) (0.14 mmol, 30 mg), InCl_3_ (0.01 mmol, 3 mg) and anhydrous MeCN (2 mL). Yield: 57%. White solid. Mp 280–281 °C. ^1^H NMR (300 MHz, DMSO-*d*_6_) δ 11.79 (s, 1H), 9.44 (s, 1H), 8.33 (s, 1H), 8.06–7.97 (m, 2H), 7.76–7.62 (m, 4H), 7.54–7.44 (m, 2H), 7.26 (dd, *J* = 3.5, 2.3 Hz, 1H), 6.84 (dd, *J* = 3.5, 1.9 Hz, 1H). ^13^C NMR (75 MHz, DMSO-*d*_6_) δ 153.3, 150.9, 150.7, 140.4, 138.8, 132.0, 131.5, 128.8 (2C), 127.8 (2C), 126.7 (2C), 122.3, 120.3 (2C), 103.9, 98.8. HRMS (ESI): calc. for C_18_H_13_ClN_4_ [M + H]^+^ 321.0902; found: 321.0903. HPLC-MS (M + H)^+^ = 321.2, *R*_t_ = 3.17 min (99%).

#### 4′-((7*H*-Pyrrolo­[2,3-*d*]­pyrimidin-4-yl)­amino)-(1,1′-biphenyl)-4-carbonitrile (**54**)

The title compound was obtained by reaction of 4-chloro-7*H*-pyrrolo­[2,3-*d*]­pyrimidine (0.41 mmol, 63 mg), 4′-amino-(1,1′-biphenyl)-4-carbonitrile (**13**) (0.41 mmol, 80 mg), InCl_3_ (0.04 mmol, 9 mg) and anhydrous MeCN (4 mL). Yield: 63%. White solid. Mp 334–335 °C. ^1^H NMR (300 MHz, DMSO-*d*_6_) δ 11.81 (s, 1H), 9.50 (s, 1H), 8.33 (s, 1H), 8.09 (d, *J* = 8.8 Hz, 2H), 7.90 (s, 4H), 7.78 (d, *J* = 8.7 Hz, 2H), 7.28 (dd, *J* = 3.5, 2.3 Hz, 1H), 6.84 (dd, *J* = 3.5, 1.9 Hz, 1H). ^13^C NMR (75 MHz, DMSO-*d*_6_) δ 153.2, 150.9, 150.7, 144.4, 141.4, 132.8 (2C), 131.1, 127.2 (2C), 126.8 (2C), 122.5, 120.2 (2C), 119.1, 109.1, 104.0, 98.7. HRMS (ESI): calc. for C_19_H_13_N_5_ [M + H]^+^ 312.1244; found: 312.1236. HPLC-MS (M + H)^+^ = 312.2, *R*_t_ = 2.84 min (99%).

#### *N*-(4′-((7*H*-Pyrrolo­[2,3-*d*]­pyrimidin-4-yl)­amino)-(1,1′-biphenyl)-4-yl)­acetamide (**55**)

The title compound was obtained by reaction of 4-chloro-7*H*-pyrrolo­[2,3-*d*]­pyrimidine (0.39 mmol, 60 mg), *N*-(4′-amino-(1,1′-biphenyl)-4-yl)­acetamide (**14**) (0.39 mmol, 88 mg), InCl_3_ (0.04 mmol, 9 mg) and anhydrous MeCN (2 mL). Yield: 56%. Beige solid. Mp 334–335 °C. ^1^H NMR (500 MHz, DMSO-*d*_6_) δ 11.76 (s, 1H), 10.00 (s, 1H), 9.38 (s, 1H), 8.30 (s, 1H), 7.98 (d, *J* = 8.8 Hz, 2H), 7.72–7.56 (m, 6H), 7.24 (dd, *J* = 3.4, 2.3 Hz, 1H), 6.82 (dd, *J* = 3.5, 2.0 Hz, 1H), 2.06 (s, 3H). ^13^C NMR (125 MHz, DMSO-*d*_6_) δ 168.3, 153.4, 150.9, 150.8, 139.6, 138.3, 134.5, 133.2, 126.3 (2C), 126.2 (2C), 122.2, 120.4 (2C), 119.3 (2C), 103.8, 98.8, 24.0. HRMS (ESI): calc. for C_20_H_17_N_5_O [M + H]^+^ 344.1506; found: 344.1505. HPLC-MS (M + H)^+^ = 344.2, *R*_t_ = 2.36 min (99%).

#### *N* ^4^,*N* ^4^-Dimethyl-*N* ^4′^-(7*H*-pyrrolo­[2,3-*d*]­pyrimidin-4-yl)-(1,1′-biphenyl)-4,4′-diamine (**56**)

The title compound was obtained by reaction of 4-chloro-7*H*-pyrrolo­[2,3-*d*]­pyrimidine (0.22 mmol, 33 mg), *N* ^4^,*N* ^4^-dimethyl-(1,1′-biphenyl)-4,4′-diamine (**15**) (0.22 mmol, 47 mg), InCl_3_ (0.02 mmol, 4 mg) and anhydrous MeCN (2 mL). Yield: 23%. Brown solid. Mp 268–269 °C. ^1^H NMR (300 MHz, DMSO-*d*_6_) δ 11.74 (s, 1H), 9.33 (s, 1H), 8.28 (s, 1H), 7.92 (d, *J* = 8.8 Hz, 2H), 7.56 (d, *J* = 8.7 Hz, 2H), 7.51 (d, *J* = 8.8 Hz, 2H), 7.23 (dd, *J* = 3.5, 2.3 Hz, 1H), 6.84–6.76 (m, 3H), 2.93 (s, 6H). ^13^C NMR (75 MHz, DMSO-*d*_6_) δ 153.5, 150.9, 150.8, 149.5, 138.6, 134.0, 127.7, 126.6 (2C), 125.5 (2C), 122.1, 120.6 (2C), 112.8 (2C), 103.7, 98.8, 40.1 (2C). HRMS (ESI): calc. for C_20_H_19_N_5_ [M + H]^+^ 330.1713; found: 330.1716. HPLC-MS (M + H)^+^ = 330.2, *R*_t_ = 2.45 min (99%).

#### *N* ^4^-Ethyl-*N* ^4′^-(7*H*-pyrrolo­[2,3-*d*]­pyrimidin-4-yl)-(1,1′-biphenyl)-4,4′-diamine (**57**)

The title compound was obtained by reaction of 4-chloro-7*H*-pyrrolo­[2,3-*d*]­pyrimidine (0.38 mmol, 58 mg), *N* ^4^-ethyl-(1,1′-biphenyl)-4,4′-diamine (**16**) (0.38 mmol, 80 mg), InCl_3_ (0.04 mmol, 9 mg) and anhydrous MeCN (2 mL). Yield: 48%. White solid. Mp 261–262 °C. ^1^H NMR (300 MHz, DMSO-*d*_6_) δ 11.73 (s, 1H), 9.30 (s, 1H), 8.28 (s, 1H), 7.90 (d, *J* = 8.7 Hz, 2H), 7.52 (d, *J* = 8.7 Hz, 2H), 7.41 (d, *J* = 8.7 Hz, 2H), 7.23 (dd, *J* = 3.5, 2.3 Hz, 1H), 6.80 (dd, *J* = 3.5, 1.9 Hz, 1H), 6.63 (d, *J* = 8.7 Hz, 2H), 5.62 (s, 1H), 3.06 (dd, *J* = 7.2, 4.7 Hz, 2H), 1.18 (t, *J* = 7.1 Hz, 3H). ^13^C NMR (75 MHz, DMSO-*d*_6_) δ 153.5, 150.8 (2C), 148.1, 138.3, 134.5, 127.2, 126.7 (2C), 125.3 (2C), 122.0, 120.6 (2C), 112.3 (2C), 103.6, 98.8, 37.3, 14.4. HRMS (ESI): calc. for C_20_H_19_N_5_ [M + H]^+^ 330.1713; found: 330.1716. HPLC-MS (M + H)^+^ = 330.2, *R*_t_ = 2.32 min (99%).

#### *N*-(4′-Morpholino-(1,1′-biphenyl)-4-yl)-7*H*-pyrrolo­[2,3-*d*]­pyrimidin-4-amine (**58**)

The title compound was obtained by reaction of 4-chloro-7*H*-pyrrolo­[2,3-*d*]­pyrimidine (0.39 mmol, 60 mg), 4′-morpholino-(1,1′-biphenyl)-4-amine (**17**) (0.39 mmol, 100 mg), InCl_3_ (0.04 mmol, 9 mg) and anhydrous MeCN (2 mL). Yield: 21%. Beige solid. Mp 350–351 °C. ^1^H NMR (300 MHz, DMSO-*d*_6_) δ 11.75 (s, 1H), 9.35 (s, 1H), 8.29 (s, 1H), 7.95 (d, *J* = 8.7 Hz, 2H), 7.59 (d, *J* = 8.8 Hz, 2H), 7.55 (d, *J* = 8.8 Hz, 2H), 7.24 (dd, *J* = 3.5, 2.3 Hz, 1H), 7.01 (d, *J* = 8.9 Hz, 2H), 6.81 (dd, *J* = 3.5, 1.9 Hz, 1H), 3.75 (dd, *J* = 6.1, 3.6 Hz, 4H), 3.14 (dd, *J* = 5.8, 4.0 Hz, 4H). ^13^C NMR (75 MHz, DMSO-*d*_6_) δ 153.4, 150.8, 150.8, 150.0, 139.0, 133.6, 130.7, 126.6 (2C), 125.8 (2C), 122.1, 120.5 (2C), 115.4 (2C), 103.7, 98.8, 66.1 (2C), 48.3 (2C). HRMS (ESI): calc. for C_22_H_21_N_5_O [M + H]^+^ 372.1819; found: 372.1818. HPLC-MS (M + H)^+^ = 372.2, *R*_t_ = 2.65 min (99%).

#### *N*-(4′-Nitro-(1,1′-biphenyl)-4-yl)-7*H*-pyrrolo­[2,3-*d*]­pyrimidin-4-amine (**59**)

The title compound was obtained by reaction of 4-chloro-7*H*-pyrrolo­[2,3-*d*]­pyrimidine (0.51 mmol, 79 mg), 4′-nitro-(1,1′-biphenyl)-4-amine (**18**) (0.51 mmol, 110 mg), InCl_3_ (0.05 mmol, 11 mg) and anhydrous MeCN (4 mL). Yield: 65%. Yellow solid. Mp 300–301 °C. ^1^H NMR (300 MHz, DMSO-*d*_6_) δ 11.82 (s, 1H), 9.55 (s, 1H), 8.35 (s, 1H), 8.31–8.26 (m, 2H), 8.16–8.09 (m, 2H), 8.00–7.94 (m, 2H), 7.85–7.79 (m, 2H), 7.28 (dd, *J* = 3.5, 2.3 Hz, 1H), 6.86 (dd, *J* = 3.5, 1.9 Hz, 1H). ^13^C NMR (75 MHz, DMSO-*d*_6_) δ 153.2, 151.0, 150.7, 146.4, 146.0, 141.7, 130.6, 127.5 (2C), 126.9 (2C), 124.2 (2C), 122.6, 120.2 (2C), 104.1, 98.8. HRMS (ESI): calc. for C_18_H_13_N_5_O_2_ [M + H]^+^ 332.1142; found: 332.1141. HPLC-MS (M + H)^+^ = 332.2, *R*_t_ = 3.00 min (99%).

#### *N*-(4-(1-Methyl-1*H*-pyrrol-2-yl)­phenyl)-7*H*-pyrrolo­[2,3-*d*]­pyrimidin-4-amine (**60**)

The title compound was obtained by reaction of 4-chloro-7*H*-pyrrolo­[2,3-*d*]­pyrimidine (0.34 mmol, 52 mg), 4-(1-methyl-1*H*-pyrrol-2-yl)­aniline (**19**) (0.34 mmol, 60 mg), InCl_3_ (0.03 mmol, 7 mg) and anhydrous MeCN (3 mL). Yield: 61%. Beige solid. Mp 216–217 °C. ^1^H NMR (500 MHz, DMSO-*d*_6_) δ 11.76 (s, 1H), 9.37 (s, 1H), 8.29 (s, 1H), 7.95 (d, *J* = 8.7 Hz, 2H), 7.40 (d, *J* = 8.6 Hz, 2H), 7.24 (dd, *J* = 3.4, 2.3 Hz, 1H), 6.83–6.78 (m, 2H), 6.12 (dd, *J* = 3.6, 1.9 Hz, 1H), 6.04 (dd, *J* = 3.5, 2.7 Hz, 1H), 3.66 (s, 3H). ^13^C NMR (125 MHz, DMSO-*d*_6_) δ 153.4, 150.9, 150.7, 139.0, 133.5, 128.1 (2C), 126.7, 123.7, 122.2, 120.0 (2C), 107.7, 107.2, 103.7, 98.7, 34.8. HRMS (ESI): calc. for C_17_H_15_N_5_ [M + H]^+^ 290.1400; found: 290.1398. HPLC-MS (M + H)^+^ = 290.2, *R*_t_ = 2.63 min (99%).

#### *N*-(4-(Furan-3-yl)­phenyl)-7*H*-pyrrolo­[2,3-*d*]­pyrimidin-4-amine (**61**)

The title compound was obtained by reaction of 4-chloro-7*H*-pyrrolo­[2,3-*d*]­pyrimidine (0.63 mmol, 96 mg), 4-(furan-3-yl)­aniline (**20**) (0.63 mmol, 100 mg), InCl_3_ (0.06 mmol, 14 mg) and anhydrous MeCN (4 mL). Yield: 57%. Beige solid. Mp 256–257 °C. ^1^H NMR (300 MHz, DMSO-*d*_6_) δ 11.76 (s, 1H), 9.35 (s, 1H), 8.30 (s, 1H), 8.11 (dd, *J* = 1.5, 0.9 Hz, 1H), 7.93 (d, *J* = 8.7 Hz, 2H), 7.72 (t, *J* = 1.7 Hz, 1H), 7.58 (d, *J* = 8.7 Hz, 2H), 7.24 (dd, *J* = 3.5, 2.3 Hz, 1H), 6.93 (dd, *J* = 1.9, 0.9 Hz, 1H), 6.80 (dd, *J* = 3.5, 1.9 Hz, 1H). ^13^C NMR (75 MHz, DMSO-*d*_6_) δ 153.4, 150.9, 150.8, 144.1, 139.3, 138.4, 125.8, 125.7 (2C), 125.6, 122.2, 120.4 (2C), 108.7, 103.7, 98.8. HRMS (ESI): calc. for C_16_H_12_N_4_O [M + H]^+^ 277.1084; found: 277.1085. HPLC-MS (M + H)^+^ = 277.1, *R*_t_ = 2.55 min (95%).

#### *N*-(4-(Furan-2-yl)­phenyl)-7*H*-pyrrolo­[2,3-*d*]­pyrimidin-4-amine (**62**)

The title compound was obtained by reaction of 4-chloro-7*H*-pyrrolo­[2,3-*d*]­pyrimidine (0.89 mmol, 136 mg), 4-(furan-2-yl)­aniline (**21**) (0.89 mmol, 142 mg), InCl_3_ (0.09 mmol, 20 mg) and anhydrous MeCN (4 mL). Yield: 61%. Beige solid. Mp 262–263 °C. ^1^H NMR (300 MHz, DMSO-*d*_6_) δ 11.78 (s, 1H), 9.43 (s, 1H), 8.31 (s, 1H), 7.99 (d, *J* = 8.6 Hz, 2H), 7.75–7.55 (m, 3H), 7.25 (dd, *J* = 3.5, 2.3 Hz, 1H), 6.94–6.64 (m, 2H), 6.56 (dd, *J* = 3.4, 1.8 Hz, 1H). ^13^C NMR (75 MHz, DMSO-*d*_6_) δ 153.4, 153.3, 150.9, 150.8, 142.2, 139.9, 124.3, 123.9 (2C), 122.4, 120.2 (2C), 112.0, 104.3, 103.9, 98.8. HRMS (ESI): calc. for C_16_H_12_N_4_O [M + H]^+^ 277.1084; found: 277.1084. HPLC-MS (M + H)^+^ = 277.1, *R*_t_ = 2.70 min (>99%).

#### Methyl 5-(4-((7*H*-Pyrrolo­[2,3-*d*]­pyrimidin-4-yl)­amino)­phenyl)­furan-2-carboxylate (**63**)

The title compound was obtained by reaction of 4-chloro-7*H*-pyrrolo­[2,3-*d*]­pyrimidine (0.65 mmol, 100 mg), methyl 5-(4-aminophenyl)­furan-2-carboxylate (0.65 mmol, 141 mg), InCl_3_ (0.06 mmol, 14 mg) and anhydrous THF (4 mL). Yield: 33%. Yellow solid. Mp 258–259 °C. ^1^H NMR (300 MHz, DMSO-*d*_6_) δ 11.82 (s, 1H), 9.53 (s, 1H), 8.34 (s, 1H), 8.08 (d, *J* = 8.9 Hz, 2H), 7.79 (d, *J* = 8.8 Hz, 2H), 7.41 (d, *J* = 3.7 Hz, 1H), 7.28 (dd, *J* = 3.5, 2.3 Hz, 1H), 7.05 (d, *J* = 3.6 Hz, 1H), 6.84 (dd, *J* = 3.5, 1.9 Hz, 1H), 3.84 (s, 3H). ^13^C NMR (75 MHz, DMSO-*d*_6_) δ 158.4, 157.3, 153.1, 151.0, 150.6, 142.3, 141.5, 125.1 (2C), 122.6, 122.3, 120.8, 119.9 (2C), 106.5, 104.0, 98.7, 51.7. HRMS (ESI): calc. for C_18_H_14_N_4_O_3_ [M + H]^+^ 335.1139; found: 335.1138. HPLC-MS (M + H)^+^ = 335.1, *R*_t_ = 2.58 min (99%).

#### *N*-(4-(Thiophen-3-yl)­phenyl)-7*H*-pyrrolo­[2,3-*d*]­pyrimidin-4-amine (**64**)

The title compound was obtained by reaction of 4-chloro-7*H*-pyrrolo­[2,3-*d*]­pyrimidine (0.61 mmol, 93 mg), 4-(thiophen-3-yl)­aniline (**22**) (0.61 mmol, 107 mg), InCl_3_ (0.06 mmol, 14 mg) and anhydrous THF (4 mL). Yield: 45%. Beige solid. Mp 290–291 °C. ^1^H NMR (300 MHz, DMSO-*d*_6_) δ 11.77 (s, 1H), 9.38 (s, 1H), 8.30 (s, 1H), 7.95 (d, *J* = 8.8 Hz, 2H), 7.77 (dd, *J* = 2.9, 1.4 Hz, 1H), 7.69 (d, *J* = 8.7 Hz, 2H), 7.62 (dd, *J* = 5.0, 2.9 Hz, 1H), 7.55 (dd, *J* = 5.0, 1.4 Hz, 1H), 7.25 (dd, *J* = 3.5, 2.3 Hz, 1H), 6.81 (dd, *J* = 3.5, 1.9 Hz, 1H). ^13^C NMR (75 MHz, DMSO-*d*_6_) δ 153.4, 150.9, 150.7, 141.4, 139.5, 129.0, 126.9, 126.2 (2C), 126.0, 122.2, 120.3 (2C), 119.4, 103.7, 98.8. HRMS (ESI): calc. for C_16_H_12_N_4_S [M + H]^+^ 292.0783; found: 292.0782. HPLC-MS (M + H)^+^ = 293.1, *R*_t_ = 2.75 min (95%).

#### 1-(3-(4-((7*H*-Pyrrolo­[2,3-*d*]­pyrimidin-4-yl)­amino)­phenyl)­thiophen-2-yl)­ethan-1-one (**65**)

The title compound was obtained by reaction of 4-chloro-7*H*-pyrrolo­[2,3-*d*]­pyrimidine (0.83 mmol, 127 mg), 1-(3-(4-aminophenyl)­thiophen-2-yl)­ethan-1-one (**23**) (0.83 mmol, 180 mg), InCl_3_ (0.08 mmol, 18 mg) and anhydrous MeCN (4 mL). Yield: 51%. Yellow solid. Mp 210–211 °C. ^1^H NMR (300 MHz, DMSO-*d*_6_) δ 11.80 (s, 1H), 9.47 (s, 1H), 8.32 (s, 1H), 8.03 (d, *J* = 8.8 Hz, 2H), 7.94 (d, *J* = 5.0 Hz, 1H), 7.43 (d, *J* = 8.6 Hz, 2H), 7.27 (dd, *J* = 3.5, 2.3 Hz, 1H), 7.20 (d, *J* = 5.0 Hz, 1H), 6.84 (dd, *J* = 3.5, 1.9 Hz, 1H), 2.18 (s, 3H). ^13^C NMR (75 MHz, DMSO-*d*_6_) δ 191.4, 153.3, 150.9, 150.7, 146.6, 140.7, 138.5, 132.3, 132.1, 129.5 (2C), 129.0, 122.4, 119.5 (2C), 103.9, 98.7, 29.0. HRMS (ESI): calc. for C_18_H_14_N_4_OS [M + H]^+^ 335.0961; found: 335.0959. HPLC-MS (M + H)^+^ = 335.1, *R*_t_ = 2.63 min (99%).

#### ^*t*^ *B*utyl (4-(4-((7*H*-Pyrrolo­[2,3-*d*]­pyrimidin-4-yl)­amino)­phenyl)­thiophen-2-yl)­carbamate (**66**)

The title compound was obtained by reaction of 4-chloro-7*H*-pyrrolo­[2,3-*d*]­pyrimidine (0.47 mmol, 72 mg), ^*t*^butyl (4-(4-aminophenyl)­thiophen-2-yl)­carbamate (**24**) (0.47 mmol, 130 mg), InCl_3_ (0.05 mmol, 11 mg) and anhydrous THF (4 mL). Yield: 10%. Red solid. Mp 226–227 °C. ^1^H NMR (500 MHz, DMSO-*d*_6_) δ 11.76 (s, 1H), 10.48 (s, 1H), 9.37 (s, 1H), 8.28 (s, 1H), 7.96–7.88 (m, 2H), 7.59–7.50 (m, 2H), 7.24 (dd, *J* = 3.5, 2.3 Hz, 1H), 7.12 (d, *J* = 1.8 Hz, 1H), 6.87 (d, *J* = 1.8 Hz, 1H), 6.80 (dd, *J* = 3.5, 1.9 Hz, 1H), 1.49 (s, 9H). ^13^C NMR (125 MHz, DMSO-*d*_6_) δ 153.3, 152.5, 150.8, 150.7, 141.9, 139.3, 138.1, 129.3, 125.7 (2C), 122.2, 120.4 (2C), 109.7, 108.3, 103.7, 98.8, 79.9, 28.1 (3C). HRMS (ESI): calc. for C_21_H_21_N_5_O_2_S [M + H]^+^ 408.1489; found: 408.1488. HPLC-MS (M + H)^+^ = 408.2, *R*_t_ = 2.95 min (95%).

#### *N*-(4-(2-Chlorothiophen-3-yl)­phenyl)-7*H*-pyrrolo­[2,3-*d*]­pyrimidin-4-amine (**67**)

The title compound was obtained by reaction of 4-chloro-7*H*-pyrrolo­[2,3-*d*]­pyrimidine (0.95 mmol, 145 mg), 4-(2-chlorothiophen-3-yl)­aniline (**25**) (0.95 mmol, 200 mg), InCl_3_ (0.09 mmol, 21 mg) and anhydrous MeCN (4 mL). Yield: 77%. White solid. Mp 244–245 °C. ^1^H NMR (300 MHz, DMSO-*d*_6_) δ 11.79 (s, 1H), 9.46 (s, 1H), 8.32 (s, 1H), 8.03 (d, *J* = 8.8 Hz, 2H), 7.63–7.50 (m, 3H), 7.28–7.22 (m, 2H), 6.83 (dd, *J* = 3.5, 1.9 Hz, 1H). ^13^C NMR (75 MHz, DMSO-*d*_6_) δ 153.3, 150.9, 150.7, 140.1, 137.7, 128.7, 128.3 (2C), 126.9, 124.3, 122.4, 122.1, 119.9 (2C), 103.9, 98.8. HRMS (ESI): calc. for C_16_H_11_ClN_4_S [M + H]^+^ 327.0466; found: 327.0467. HPLC-MS (M + H)^+^ = 327.1, *R*_t_ = 3.07 min (99%).

#### *N*-(4-(Thiophen-2-yl)­phenyl)-7*H*-pyrrolo­[2,3-*d*]­pyrimidin-4-amine (**68**)

The title compound was obtained by reaction of 4-chloro-7*H*-pyrrolo­[2,3-*d*]­pyrimidine (0.28 mmol, 43 mg), 4-(thiophen-2-yl)­aniline (**26**) (0.28 mmol, 50 mg), InCl_3_ (0.03 mmol, 6 mg) and anhydrous THF (2 mL). Yield: 45%. Beige solid. Mp 271–272 °C. ^1^H NMR (300 MHz, DMSO-*d*_6_) δ 11.78 (s, 1H), 9.42 (s, 1H), 8.30 (s, 1H), 7.97 (d, *J* = 8.7 Hz, 2H), 7.63 (d, *J* = 8.7 Hz, 2H), 7.47 (dd, *J* = 5.1, 1.1 Hz, 1H), 7.43 (dd, *J* = 3.6, 1.1 Hz, 1H), 7.25 (dd, *J* = 3.5, 2.4 Hz, 1H), 7.12 (dd, *J* = 5.1, 3.6 Hz, 1H), 6.82 (dd, *J* = 3.5, 1.9 Hz, 1H). ^13^C NMR (75 MHz, DMSO-*d*_6_) δ 153.3, 150.9, 150.7, 143.6, 140.0, 128.4, 127.4, 125.6 (2C), 124.5, 122.5, 122.3, 120.3 (2C), 103.8, 98.7. HRMS (ESI): calc. for C_16_H_12_N_4_S [M + H]^+^ 293.0855; found: 293.0851. HPLC-MS (M + H)^+^ = 293.1, *R*_t_ = 2.80 min (99%).

#### *N*-(4-(5-Methylthiophen-2-yl)­phenyl)-7*H*-pyrrolo­[2,3-*d*]­pyrimidin-4-amine (**69**)

The title compound was obtained by reaction of 4-chloro-7*H*-pyrrolo­[2,3-*d*]­pyrimidine (0.31 mmol, 47 mg), 4-(5-methylthiophen-2-yl)­aniline (**27**) (0.31 mmol, 60 mg), InCl_3_ (0.03 mmol, 6 mg) and anhydrous THF (2 mL). Yield: 21%. White solid. Mp 280–281 °C. ^1^H NMR (300 MHz, DMSO-*d*_6_) δ 11.77 (s, 1H), 9.39 (s, 1H), 8.29 (s, 1H), 7.94 (d, *J* = 8.7 Hz, 2H), 7.55 (d, *J* = 8.6 Hz, 2H), 7.25–7.23 (m, 1H), 7.20 (d, *J* = 3.6 Hz, 1H), 6.85–6.73 (m, 2H), 2.46 (s, 3H). ^13^C NMR (75 MHz, DMSO-*d*_6_) δ 153.3, 150.9, 150.7, 141.2, 139.7, 137.9, 127.8, 126.6, 125.2 (2C), 122.3, 122.2, 120.4 (2C), 103.8, 98.7, 15.1. HRMS (ESI): calc. for C_17_H_14_N_4_S [M + H]^+^ 307.1012; found: 307.1001. HPLC-MS (M + H)^+^ = 307.1, *R*_t_ = 3.02 min (99%).

#### *N*-(4-(4-Methylthiophen-2-yl)­phenyl)-7*H*-pyrrolo­[2,3-*d*]­pyrimidin-4-amine (**70**)

The title compound was obtained by reaction of 4-chloro-7*H*-pyrrolo­[2,3-*d*]­pyrimidine (0.44 mmol, 67 mg), 4-(4-methylthiophen-2-yl)­aniline (**28**) (0.44 mmol, 85 mg), InCl_3_ (0.04 mmol, 9 mg) and anhydrous THF (2 mL). Yield: 42%. White solid. Mp 263–264 °C. ^1^H NMR (300 MHz, DMSO-*d*_6_) δ 11.78 (s, 1H), 9.41 (s, 1H), 8.30 (s, 1H), 7.96 (d, *J* = 8.7 Hz, 2H), 7.58 (d, *J* = 8.7 Hz, 2H), 7.25 (t, *J* = 2.5 Hz, 2H), 7.04 (t, *J* = 1.3 Hz, 1H), 6.81 (dd, *J* = 3.5, 1.9 Hz, 1H), 2.23 (d, *J* = 1.1 Hz, 3H). ^13^C NMR (75 MHz, DMSO-*d*_6_) δ 153.3, 150.9, 150.7, 143.3, 139.9, 138.3, 127.6, 125.4 (2C), 124.7, 122.3, 120.3 (2C), 119.6, 103.8, 98.7, 15.6. HRMS (ESI): calc. for C_17_H_14_N_4_S [M + H]^+^ 307.1012; found: 307.1006. HPLC-MS (M + H)^+^ = 307.1, *R*_t_ = 3.01 min (99%).

#### *N*-(4-(3-Methylthiophen-2-yl)­phenyl)-7*H*-pyrrolo­[2,3-*d*]­pyrimidin-4-amine (**71**)

The title compound was obtained by reaction of 4-chloro-7*H*-pyrrolo­[2,3-*d*]­pyrimidine (0.79 mmol, 120 mg), 4-(3-methylthiophen-2-yl)­aniline (**29**) (0.79 mmol, 150 mg), InCl_3_ (0.08 mmol, 18 mg) and anhydrous MeCN (4 mL). Yield: 68%. White solid. Mp 263–264 °C. ^1^H NMR (300 MHz, DMSO-*d*_6_) δ 11.79 (s, 1H), 9.44 (s, 1H), 8.31 (s, 1H), 8.01 (d, *J* = 8.7 Hz, 2H), 7.44 (d, *J* = 8.7 Hz, 2H), 7.39 (d, *J* = 5.1 Hz, 1H), 7.26 (dd, *J* = 3.5, 2.3 Hz, 1H), 6.98 (d, *J* = 5.1 Hz, 1H), 6.83 (dd, *J* = 3.5, 1.9 Hz, 1H), 2.30 (s, 3H). ^13^C NMR (75 MHz, DMSO-*d*_6_) δ 153.4, 150.9, 150.7, 139.7, 137.1, 132.3, 131.4, 128.6 (2C), 127.6, 123.3, 122.3, 120.1 (2C), 103.8, 98.8, 14.8. HRMS (ESI): calc. for C_17_H_14_N_4_S [M + H]^+^ 307.1012; found: 307.1002. HPLC-MS (M + H)^+^ = 307.1, *R*_t_ = 2.98 min (99%).

#### 1-(5-(4-((7*H*-Pyrrolo­[2,3-*d*]­pyrimidin-4-yl)­amino)­phenyl)­thiophen-2-yl)­ethan-1-one (**72**)

The title compound was obtained by reaction of 4-chloro-7*H*-pyrrolo­[2,3-*d*]­pyrimidine (0.19 mmol, 30 mg), 1-(5-(4-aminophenyl)­thiophen-2-yl)­ethan-1-one (**30**) (0.19 mmol, 43 mg), InCl_3_ (0.02 mmol, 4 mg) and anhydrous MeCN (3 mL). Yield: 73%. Yellow solid. Mp 260–261 °C. ^1^H NMR (300 MHz, DMSO-*d*_6_) δ 11.86 (s, 1H), 9.58 (s, 1H), 8.33 (s, 1H), 8.11–8.00 (m, 2H), 7.93 (d, *J* = 4.0 Hz, 1H), 7.79–7.71 (m, 2H), 7.56 (d, *J* = 4.0 Hz, 1H), 7.27 (d, *J* = 3.5 Hz, 1H), 6.84 (d, *J* = 3.4 Hz, 1H), 2.53 (s, 3H). ^13^C NMR (75 MHz, DMSO-*d*_6_) δ 190.4, 153.1, 152.1, 151.0, 150.6, 141.7, 141.6, 135.3, 126.4 (2C), 126.0, 123.6, 122.6, 120.1 (2C), 104.0, 98.7, 26.3. HRMS (ESI): calc. for C_18_H_14_N_4_OS [M + H]^+^ 334.0888; found: 334.0890. HPLC-MS (M + H)^+^ = 335.2, *R*_t_ = 2.76 min (99%).

#### *N*-(4-(5-(Morpholinomethyl)­thiophen-2-yl)­phenyl)-7*H*-pyrrolo­[2,3-*d*]­pyrimidin-4-amine (**73**)

The title compound was obtained by reaction of 4-chloro-7*H*-pyrrolo­[2,3-*d*]­pyrimidine (0.49 mmol, 75 mg), 4-(5-(morpholinomethyl)­thiophen-2-yl)­aniline (**31**) (0.49 mmol, 134 mg), InCl_3_ (0.05 mmol, 11 mg) and anhydrous MeCN (3 mL). Yield: 59%. Yellow solid. Mp 227–228 °C. ^1^H NMR (300 MHz, DMSO-*d*_6_) δ 11.77 (s, 1H), 9.36 (s, 1H), 8.31 (s, 1H), 7.95 (d, *J* = 8.7 Hz, 2H), 7.68–7.61 (m, 3H), 7.38 (d, *J* = 1.5 Hz, 1H), 7.24 (dd, *J* = 3.5, 2.3 Hz, 1H), 6.81 (dd, *J* = 3.5, 1.9 Hz, 1H), 3.69 (s, 2H), 3.59 (t, *J* = 4.6 Hz, 4H), 2.43 (t, *J* = 4.6 Hz, 4H). ^13^C NMR (75 MHz, DMSO-*d*_6_) δ 153.4, 150.9, 150.8, 142.2, 140.5, 139.5, 129.0, 126.0 (2C), 125.0, 122.2, 120.3 (2C), 119.0, 103.8, 98.8, 66.2 (2C), 56.9, 52.9 (2C). HRMS (ESI): calc. for C_21_H_21_N_5_OS [M + H]^+^ 392.1540; found: 392.1539. HPLC-MS (M + H)^+^ = 392.2, *R*_t_ = 1.97 min (98%).

#### *N*-(4-(5-Chlorothiophen-2-yl)­phenyl)-7*H*-pyrrolo­[2,3-*d*]­pyrimidin-4-amine (**74**)

The title compound was obtained by reaction of 4-chloro-7*H*-pyrrolo­[2,3-*d*]­pyrimidine (0.90 mmol, 138 mg), 4-(5-chlorothiophen-2-yl)­aniline (**32**) (0.90 mmol, 190 mg), InCl_3_ (0.09 mmol, 20 mg) and anhydrous MeCN (3 mL). Yield: 65%. White solid. Mp 288–289 °C. ^1^H NMR (300 MHz, DMSO-*d*_6_) δ 11.92 (s, 1H), 9.74 (s, 1H), 8.31 (s, 1H), 7.97 (d, *J* = 8.7 Hz, 2H), 7.60 (d, *J* = 8.7 Hz, 2H), 7.32 (d, *J* = 3.9 Hz, 1H), 7.28 (dd, *J* = 3.5, 2.3 Hz, 1H), 7.13 (d, *J* = 3.9 Hz, 1H), 6.87 (dd, *J* = 3.5, 1.8 Hz, 1H). ^13^C NMR (75 MHz, DMSO-*d*_6_) δ 152.9, 150.4, 149.8, 142.6, 140.0, 128.2, 126.9, 126.2, 125.5 (2C), 122.7, 122.3, 120.8 (2C), 103.8, 99.2. HRMS (ESI): calc. for C_16_H_11_ClN_4_S [M + H]^+^ 327.0466; found: 327.0466. HPLC-MS (M + H)^+^ = 327.1, *R*_t_ = 3.27 min (97%).

#### *N*-(4-(5-Nitrothiophen-2-yl)­phenyl)-7*H*-pyrrolo­[2,3-*d*]­pyrimidin-4-amine (**75**)

The title compound was obtained by reaction of 4-chloro-7*H*-pyrrolo­[2,3-*d*]­pyrimidine (1.27 mmol, 195 mg), 4-(5-nitrothiophen-2-yl)­aniline (**33**) (1.27 mmol, 280 mg), InCl_3_ (0.13 mmol, 29 mg) and anhydrous MeCN (5 mL). Yield: 30%. Red solid. Mp 309–310 °C. ^1^H NMR (300 MHz, DMSO-*d*_6_) δ 11.86 (s, 1H), 9.63 (s, 1H), 8.36 (s, 1H), 8.16 (d, *J* = 4.4 Hz, 1H), 8.11 (d, *J* = 8.8 Hz, 2H), 7.84 (d, *J* = 8.8 Hz, 2H), 7.59 (d, *J* = 4.4 Hz, 1H), 7.30 (dd, *J* = 3.5, 2.3 Hz, 1H), 6.85 (dd, *J* = 3.5, 1.9 Hz, 1H). ^13^C NMR (75 MHz, DMSO-*d*_6_) δ 152.9, 152.5, 151.1, 150.6, 147.9, 142.8, 131.7, 126.8 (2C), 124.6, 122.8, 122.6, 119.9 (2C), 104.2, 98.7. HRMS (ESI): calc. for C_16_H_11_N_5_O_2_S [M + H]^+^ 338.0706; found: 338.0706. HPLC-MS (M + H)^+^ = 338.1, *R*_t_ = 3.16 min (99%).

#### 5-(4-((7*H*-Pyrrolo­[2,3-*d*]­pyrimidin-4-yl)­amino)­phenyl)-*N*-methylthiophene-2-carboxamide (**76**)

The title compound was obtained by reaction of 4-chloro-7*H*-pyrrolo­[2,3-*d*]­pyrimidine (0.52 mmol, 80 mg), 5-(4-aminophenyl)-*N*-methylthiophene-2-carboxamide (**34**) (0.52 mmol, 120 mg), InCl_3_ (0.05 mmol, 11 mg) and anhydrous MeCN (4 mL). Yield: 17%. Brown solid. Mp 350–351 °C. ^1^H NMR (500 MHz, DMSO-*d*_6_) δ 11.80 (s, 1H), 9.48 (s, 1H), 8.43 (q, *J* = 4.5 Hz, 1H), 8.32 (s, 1H), 8.05–7.97 (m, 2H), 7.71–7.61 (m, 3H), 7.43 (d, *J* = 3.8 Hz, 1H), 7.27 (dd, *J* = 3.5, 2.3 Hz, 1H), 6.83 (dd, *J* = 3.5, 1.9 Hz, 1H), 2.78 (d, *J* = 4.6 Hz, 3H). ^13^C NMR (125 MHz, DMSO-*d*_6_) δ 161.5, 153.2, 150.9, 150.7, 147.5, 140.9, 137.8, 128.8, 126.6, 125.9 (2C), 122.9, 122.5, 120.2 (2C), 103.9, 98.7, 26.0. HRMS (ESI): calc. for C_18_H_15_N_5_OS [M + H]^+^ 350.1070; found: 350.1071. HPLC-MS (M + H)^+^ = 350.2, *R*_t_ = 2.36 min (99%).

#### *N*-(3′-((7*H*-Pyrrolo­[2,3-*d*]­pyrimidin-4-yl)­amino)-(1,1′-biphenyl)-4-yl)­acetamide (**77**)

The title compound was obtained by reaction of 4-chloro-7*H*-pyrrolo­[2,3-*d*]­pyrimidine (0.41 mmol, 63 mg), *N*-(3′-amino-(1,1′-biphenyl)-4-yl)­acetamide (**35**) (0.41 mmol, 93 mg), InCl_3_ (0.04 mmol, 9 mg) and anhydrous MeCN (3 mL). Yield: 36%. White solid. Mp 261–262 °C. ^1^H NMR (300 MHz, DMSO-*d*_6_) δ 11.76 (s, 1H), 10.04 (s, 1H), 9.36 (s, 1H), 8.30 (s, 1H), 8.10 (t, *J* = 1.9 Hz, 1H), 7.95 (ddd, *J* = 8.1, 2.2, 1.0 Hz, 1H), 7.73–7.66 (m, 2H), 7.64–7.58 (m, 2H), 7.40 (t, *J* = 7.9 Hz, 1H), 7.31–7.22 (m, 2H), 6.81 (dd, *J* = 3.5, 1.9 Hz, 1H), 2.07 (s, 3H). ^13^C NMR (75 MHz, DMSO-*d*_6_) δ 168.3, 153.5, 150.9, 150.8, 140.9, 140.1, 138.8, 134.9, 129.0, 126.8 (2C), 122.2, 120.0, 119.3 (2C), 118.8, 118.0, 103.7, 98.7, 24.0. HRMS (ESI): calc. for C_20_H_17_N_5_O [M + H]^+^ 344.1506; found: 344.1504. HPLC-MS (M + H)^+^ = 344.2, *R*_t_ = 2.38 min (99%).

#### *N*-(3-(Furan-3-yl)­phenyl)-7*H*-pyrrolo­[2,3-*d*]­pyrimidin-4-amine (**78**)

The title compound was obtained by reaction of 4-chloro-7*H*-pyrrolo­[2,3-*d*]­pyrimidine (0.60 mmol, 92 mg), 3-(furan-3-yl)­aniline (**36**) (0.60 mmol, 95 mg), InCl_3_ (0.06 mmol, 13 mg) and anhydrous MeCN (4 mL). Yield: 70%. White solid. Mp 222–223 °C. ^1^H NMR (300 MHz, DMSO-*d*_6_) δ 11.76 (s, 1H), 9.35 (s, 1H), 8.30 (s, 1H), 8.13 (t, *J* = 1.2 Hz, 1H), 8.02 (t, *J* = 1.9 Hz, 1H), 7.88 (ddd, *J* = 8.1, 2.2, 1.2 Hz, 1H), 7.80–7.72 (m, 1H), 7.35 (t, *J* = 7.8 Hz, 1H), 7.29–7.20 (m, 2H), 6.90 (dd, *J* = 1.9, 0.9 Hz, 1H), 6.80 (dd, *J* = 3.5, 1.8 Hz, 1H). ^13^C NMR (75 MHz, DMSO-*d*_6_) δ 153.6, 150.9, 150.8, 144.3, 140.9, 139.1, 132.1, 129.0, 126.1, 122.2, 119.6, 119.2, 117.5, 108.8, 103.7, 98.8. HRMS (ESI): calc. for C_16_H_12_N_4_O [M + H]^+^ 277.1084; found: 277.1081. HPLC-MS (M + H)^+^ = 277.2, *R*_t_ = 2.61 min (99%).

#### *N*-(3-(Furan-2-yl)­phenyl)-7*H*-pyrrolo­[2,3-*d*]­pyrimidin-4-amine (**79**)

The title compound was obtained by reaction of 4-chloro-7*H*-pyrrolo­[2,3-*d*]­pyrimidine (1.39 mmol, 213 mg), 3-(furan-2-yl)­aniline (**37**) (1.39 mmol, 221 mg), InCl_3_ (0.14 mmol, 31 mg) and anhydrous MeCN (5 mL). Yield: 51%. White solid. Mp 216–217 °C. ^1^H NMR (300 MHz, DMSO-*d*_6_) δ 11.80 (s, 1H), 9.42 (s, 1H), 8.34 (s, 1H), 8.27–8.18 (m, 1H), 7.95 (dt, *J* = 7.3, 2.1 Hz, 1H), 7.76 (dd, *J* = 1.8, 0.8 Hz, 1H), 7.43–7.32 (m, 2H), 7.27 (dd, *J* = 3.5, 2.3 Hz, 1H), 6.89 (dd, *J* = 3.4, 0.8 Hz, 1H), 6.85 (dd, *J* = 3.5, 1.8 Hz, 1H), 6.60 (dd, *J* = 3.4, 1.8 Hz, 1H). ^13^C NMR (75 MHz, DMSO-*d*_6_) δ 153.6, 153.3, 151.0, 150.8, 142.8, 141.0, 130.6, 129.1, 122.3, 119.3, 117.4, 115.0, 112.1, 105.8, 103.8, 98.8. HRMS (ESI): calc. for C_16_H_12_N_4_O [M + H]^+^ 277.1084; found: 277.1086. HPLC-MS (M + H)^+^ = 277.2, *R*_t_ = 2.64 min (99%).

#### *N*-(3-(Thiophen-3-yl)­phenyl)-7*H*-pyrrolo­[2,3-*d*]­pyrimidin-4-amine (**80**)

The title compound was obtained by reaction of 4-chloro-7*H*-pyrrolo­[2,3-*d*]­pyrimidine (0.97 mmol, 149 mg), 3-(thiophen-3-yl)­aniline (**38**) (0.97 mmol, 170 mg), InCl_3_ (0.09 mmol, 22 mg) and anhydrous MeCN (4 mL). Yield: 81%. White solid. Mp 239–240 °C. ^1^H NMR (300 MHz, DMSO-*d*_6_) δ 11.77 (s, 1H), 9.36 (s, 1H), 8.31 (s, 1H), 8.15 (dt, *J* = 2.0, 1.3 Hz, 1H), 7.91 (dt, *J* = 7.0, 2.3 Hz, 1H), 7.80 (dd, *J* = 2.9, 1.4 Hz, 1H), 7.66 (dd, *J* = 5.0, 2.9 Hz, 1H), 7.51 (dd, *J* = 5.0, 1.4 Hz, 1H), 7.42–7.33 (m, 2H), 7.25 (dd, *J* = 3.5, 1.9 Hz, 1H), 6.81 (dd, *J* = 3.4, 1.5 Hz, 1H). ^13^C NMR (75 MHz, DMSO-*d*_6_) δ 153.6, 150.9, 150.8, 141.7, 140.9, 135.4, 129.0, 127.1, 126.1, 122.2, 120.8, 120.0, 119.2, 118.0, 103.7, 98.7. HRMS (ESI): calc. for C_16_H_12_N_4_S [M + H]^+^ 293.0855; found: 293.0857. HPLC-MS (M + H)^+^ = 293.2, *R*_t_ = 2.71 min (99%).

#### 5-(3-((7*H*-Pyrrolo­[2,3-*d*]­pyrimidin-4-yl)­amino)­phenyl)-*N*-methylthiophene-2-carboxamide (**81**)

The title compound was obtained by reaction of 4-chloro-7*H*-pyrrolo­[2,3-*d*]­pyrimidine (1.3 mmol, 200 mg), 5-(3-aminophenyl)-*N*-methylthiophene-2-carboxamide (**39**) (1.3 mmol, 300 mg), InCl_3_ (0.13 mmol, 28 mg) and anhydrous MeCN (4 mL). Yield: 90%. Pale yellow solid. Mp 276–277 °C. ^1^H NMR (300 MHz, DMSO-*d*_6_) δ 11.79 (s, 1H), 9.42 (s, 1H), 8.49 (d, *J* = 4.9 Hz, 1H), 8.32 (s, 1H), 8.24 (d, *J* = 2.0 Hz, 1H), 7.98 (dt, *J* = 7.8, 1.6 Hz, 1H), 7.70 (d, *J* = 3.9 Hz, 1H), 7.49 (d, *J* = 3.9 Hz, 1H), 7.44–7.33 (m, 2H), 7.27 (dd, *J* = 3.5, 2.3 Hz, 1H), 6.81 (dd, *J* = 3.5, 1.9 Hz, 1H), 2.79 (d, *J* = 4.5 Hz, 3H). ^13^C NMR (75 MHz, DMSO-*d*_6_) δ 161.4, 153.4, 150.9, 150.7, 147.4, 141.2, 138.9, 133.3, 129.4, 128.8, 124.0, 122.4, 120.0, 119.1, 117.0, 103.8, 98.7, 26.0. HRMS (ESI): calc. for C_18_H_15_N_5_OS [M – H]^−^ 348.0925; found: 348.0921. HPLC-MS (M + H)^+^ = 350.2, *R*_t_ = 2.38 min (97%).

#### 2′-((7*H*-Pyrrolo­[2,3-*d*]­pyrimidin-4-yl)­amino)-(1,1′-biphenyl)-2-ol (**82**)

The title compound was obtained by reaction of 4-chloro-7*H*-pyrrolo­[2,3-*d*]­pyrimidine (1.48 mmol, 227 mg), 2′-amino-(1,1′-biphenyl)-2-ol (**40**) (1.48 mmol, 274 mg), InCl_3_ (0.15 mmol, 33 mg) and anhydrous MeCN (4 mL). Yield: 17%. White solid. ^1^H NMR (300 MHz, DMSO-*d*_6_) δ 11.62 (s, 1H), 8.09 (s, 1H), 7.97 (dd, *J* = 8.1, 1.3 Hz, 1H), 7.31 (ddd, *J* = 12.2, 7.5, 1.6 Hz, 2H), 7.19–7.09 (m, 2H), 7.08–7.00 (m, 1H), 6.96 (t, *J* = 3.1 Hz, 2H), 6.65–6.51 (m, 1H), 6.48 (d, *J* = 3.4 Hz, 1H). ^13^C NMR (75 MHz, DMSO-*d*_6_) δ 153.8, 151.1, 150.6, 137.9, 134.6, 131.7, 130.94, 128.5, 127.9, 126.1, 123.1, 123.1, 121.6, 118.0, 115.5, 115.3, 103.5, 98.4, 40.3, 40.1, 39.8, 39.5, 39.2, 39.0, 38.7. HRMS (ESI): calc. for C_18_H_14_N_4_O [M + H]^+^ 303.1168; found: 303.1166. HPLC-MS (M + H)^+^ = 303.2, *R*_t_ = 2.41 min (95%).

#### *N*-(2-(Furan-3-yl)­phenyl)-7*H*-pyrrolo­[2,3-*d*]­pyrimidin-4-amine (**83**)

The title compound was obtained by reaction of 4-chloro-7*H*-pyrrolo­[2,3-*d*]­pyrimidine (1.36 mmol, 209 mg), 2-(furan-3-yl)­aniline (**41**) (1.36 mmol, 240 mg), InCl_3_ (0.14 mmol, 30 mg) and anhydrous MeCN (4 mL). Yield: 11%. White solid. Mp 218–219 °C. ^1^H NMR (300 MHz, DMSO-*d*_6_) δ 11.64 (s, 1H), 9.04 (s, 1H), 8.09 (s, 1H), 7.88 (t, *J* = 1.1 Hz, 1H), 7.74–7.58 (m, 2H), 7.52–7.44 (m, 1H), 7.40–7.30 (m, 2H), 7.09 (dd, *J* = 3.5, 1.9 Hz, 1H), 6.81 (dd, *J* = 1.9, 0.8 Hz, 1H), 6.24–6.10 (m, 1H). ^13^C NMR (75 MHz, DMSO-*d*_6_) δ 155.5, 151.3, 151.1, 143.2, 140.4, 136.1, 130.0, 129.8, 128.7, 127.4, 126.6, 123.2, 121.7, 110.4, 102.7, 98.9. HRMS (ESI): calc. for C_16_H_12_N_4_O [M + H]^+^ 277.1084; found: 277.1083. HPLC-MS (M + H)^+^ = 277.2, *R*_t_ = 2.37 min (99%).

#### *N*-(2-(Furan-2-yl)­phenyl)-7*H*-pyrrolo­[2,3-*d*]­pyrimidin-4-amine (**84**)

The title compound was obtained by reaction of 4-chloro-7*H*-pyrrolo­[2,3-*d*]­pyrimidine (0.85 mmol, 131 mg), 2-(furan-2-yl)­aniline (**42**) (0.85 mmol, 150 mg), InCl_3_ (0.08 mmol, 18 mg) and anhydrous MeCN (3 mL). Yield: 55%. White solid. Mp 218–219 °C. ^1^H NMR (300 MHz, DMSO-*d*_6_) δ 11.65 (s, 1H), 9.11 (s, 1H), 8.07 (s, 1H), 7.87–7.79 (m, 1H), 7.72 (dd, *J* = 1.8, 0.7 Hz, 1H), 7.61–7.48 (m, 1H), 7.41–7.31 (m, 2H), 7.12 (dd, *J* = 3.5, 2.3 Hz, 1H), 6.67 (dd, *J* = 3.4, 0.8 Hz, 1H), 6.50 (dd, *J* = 3.4, 1.8 Hz, 1H), 6.27 (dd, *J* = 3.5, 1.9 Hz, 1H). ^13^C NMR (75 MHz, DMSO-*d*_6_) δ 155.1, 151.2, 151.1, 150.5, 142.4, 134.7, 129.5, 127.7, 127.4, 126.3, 126.1, 121.8, 112.0, 109.0, 102.8, 98.7. HRMS (ESI): calc. for C_16_H_12_N_4_O [M + H]^+^ 277.1084; found: 277.1086. HPLC-MS (M + H)^+^ = 277.2, *R*_t_ = 2.42 min (99%).

### General Procedure for the Synthesis of Derivatives 88–95

One Equiv. of *N*-(4-bromophenyl)-7*H*-pyrrolo­[2,3-*d*]­pyrimidin-4-amine (**85**), *N*-(4-iodophenyl)-7*H*-pyrrolo­[2,3-*d*]­pyrimidin-4-amine (**86**), or *N*-(3-bromophenyl)-7*H*-pyrrolo­[2,3-*d*]­pyrimidin-4-amine (**87**) was added to a previously degassed solution of the corresponding arylborane (1.2 equiv); K_3_PO_4_ (4.0 or 5.0 equiv. if the arylborane is in its chloride salt form), or Na_2_CO_3_ (2.5 equiv); and Pd­(PPh_3_)_4_ (0.06 equiv) in a mixture of dioxane/H_2_O/EtOH, 2:1.5:1. The reaction mixture was purged with argon for 15 min and subsequently heated under MW at 120 °C for 20 min. Then, the solvent was evaporated under reduced pressure and the crude residue was dissolved in 20 mL of CH_2_Cl_2_ and washed with water and brine. The organic layer was dried over MgSO_4_ and evaporated under high vacuum. Finally, the corresponding crude was purified by flash chromatography using CH_2_Cl_2_/MeOH (32:1) as eluent.

#### *N* ^4′^-(7*H*-Pyrrolo­[2,3-*d*]­pyrimidin-4-yl)-(1,1′-biphenyl)-2,4′-diamine (**88**)

The title compound was obtained by reaction of *N*-(4-bromophenyl)-7*H*-pyrrolo­[2,3-*d*]­pyrimidin-4-amine (**85**) (0.86 mmol, 250 mg), (2-aminophenyl)­boronic acid hydrochloride (1.03 mmol, 178 mg), K_3_PO_4_ (4.3 mmol, 911 mg), Pd­(PPh_3_)_4_ (0.05 mmol, 58 mg) and a solution of dioxane/H_2_O/EtOH (4.5 mL). Yield: 42%. White solid. Mp 228–229 °C. ^1^H NMR (300 MHz, DMSO-*d*_6_) δ 11.76 (s, 1H), 9.38 (s, 1H), 8.30 (s, 1H), 7.99 (d, *J* = 8.6 Hz, 2H), 7.45–7.33 (m, 2H), 7.25 (dd, *J* = 3.5, 2.4 Hz, 1H), 7.07–6.98 (m, 2H), 6.83 (dd, *J* = 3.5, 1.9 Hz, 1H), 6.78–6.73 (m, 1H), 6.67–6.59 (m, 1H), 4.76 (s, 2H). ^13^C NMR (75 MHz, DMSO-*d*_6_) δ 153.5, 150.9, 150.8, 145.0, 139.2, 133.1, 129.9, 128.7 (2C), 127.8, 125.8, 122.2, 120.3 (2C), 116.8, 115.2, 103.7, 98.8. HRMS (ESI): calc. for C_18_H_15_N_5_ [M + H]^+^ 302.1400; found: 302.1402. HPLC-MS (M + H)^+^ = 302.2, *R*_t_ = 2.42 min (96%).

#### *N* ^4′^-(7*H*-Pyrrolo­[2,3-*d*]­pyrimidin-4-yl)-(1,1′-biphenyl)-3,4′-diamine (**89**)

The title compound was obtained by reaction of *N*-(4-bromophenyl)-7*H*-pyrrolo­[2,3-*d*]­pyrimidin-4-amine (**85**) (0.86 mmol, 250 mg), (3-aminophenyl)­boronic acid hydrochloride (1.03 mmol, 178 mg), K_3_PO_4_ (4.3 mmol, 911 mg), Pd­(PPh_3_)_4_ (0.05 mmol, 58 mg) and a solution of dioxane/H_2_O/EtOH (4.5 mL). Yield: 21%. Beige solid. Mp 219–220 °C. ^1^H NMR (300 MHz, DMSO-*d*_6_) δ 11.79 (s, 1H), 9.42 (s, 1H), 8.30 (s, 1H), 7.96 (d, *J* = 8.7 Hz, 2H), 7.55 (d, *J* = 8.7 Hz, 2H), 7.25 (dd, *J* = 3.5, 2.2 Hz, 1H), 7.10 (t, *J* = 7.8 Hz, 1H), 6.88 (t, *J* = 2.0 Hz, 1H), 6.88–6.78 (m, 2H), 6.61–6.51 (m, 1H). ^13^C NMR (75 MHz, DMSO-*d*_6_) δ 153.3, 150.8, 150.6, 148.3, 140.7, 139.6, 134.6, 129.4, 126.5 (2C), 122.3, 120.5 (2C), 114.5, 113.0, 112.1, 103.7, 98.9. HRMS (ESI): calc. for C_18_H_15_N_5_ [M + H]^+^ 302.1400; found: 302.1402. HPLC-MS (M + H)^+^ = 302.2, *R*_t_ = 2.13 min (97%).

#### *N*-(4-(1*H*-Pyrazol-4-yl)­phenyl)-7*H*-pyrrolo­[2,3-*d*]­pyrimidin-4-amine (**90**)

The title compound was obtained by reaction of *N*-(4-bromophenyl)-7*H*-pyrrolo­[2,3-*d*]­pyrimidin-4-amine (**85**) (0.35 mmol, 100 mg), 4-(4,4,5,5-tetramethyl-1,3,2-dioxaborolan-2-yl)-1*H*-pyrazole (0.41 mmol, 88 mg), K_3_PO_4_ (1.38 mmol, 368 mg), Pd­(PPh_3_)_4_ (0.02 mmol, 24 mg) and a solution of dioxane/H_2_O/EtOH (4.5 mL). Yield: 15%. White solid. Mp 288–289 °C. ^1^H NMR (500 MHz, DMSO-*d*_6_) δ 12.87 (s, 1H), 11.73 (s, 1H), 9.29 (s, 1H), 8.27 (s, 1H), 8.11 (s, 1H), 7.92–7.83 (m, 3H), 7.61–7.54 (m, 2H), 7.22 (dd, *J* = 3.4, 2.3 Hz, 1H), 6.78 (dd, *J* = 3.5, 1.9 Hz, 1H). ^13^C NMR (125 MHz, DMSO-*d*_6_) δ 153.5, 150.8, 150.8, 138.3, 135.9, 126.8, 125.2 (2C), 124.8, 122.0, 121.2, 120.6 (2C), 103.6, 98.8. HRMS (ESI): calc. for C_15_H_12_N_6_ [M + H]^+^ 277.1196; found: 277.1195. HPLC-MS (M + H)^+^ = 277.2, *R*_t_ = 1.83 min (99%).

#### *N*-(4-(1*H*-Pyrazol-3-yl)­phenyl)-7*H*-pyrrolo­[2,3-*d*]­pyrimidin-4-amine (**91**)

The title compound was obtained by reaction of *N*-(4-bromophenyl)-7*H*-pyrrolo­[2,3-*d*]­pyrimidin-4-amine (**85**) (1.20 mmol, 350 mg), 3-(4,4,5,5-tetramethyl-1,3,2-dioxaborolan-2-yl)-1*H*-pyrazole (1.40 mmol, 279 mg), K_3_PO_4_ (4.8 mmol, 1.02 g), Pd­(PPh_3_)_4_ (0.07 mmol, 83 mg) and a solution of dioxane/H_2_O/EtOH (9 mL). Yield: 10%. White solid. Mp 270–271 °C. ^1^H NMR (500 MHz, DMSO-*d*_6_) δ 12.78 (s, 1H), 11.76 (s, 1H), 9.37 (s, 2H), 8.30 (s, 1H), 7.95 (d, *J* = 8.2 Hz, 3H), 7.76 (s, 3H), 7.24 (dd, *J* = 3.5, 2.3 Hz, 1H), 6.81 (dd, *J* = 3.5, 1.9 Hz, 1H), 6.68–6.62 (m, 1H). ^13^C NMR (125 MHz, DMSO-*d*_6_) δ 153.4, 150.9, 150.8, 139.8, 131.4, 128.7, 125.3 (2C), 122.2, 120.2 (2C), 104.2, 103.8, 101.4, 98.8. HRMS (ESI): calc. for C_15_H_12_N_6_ [M + H]^+^ 277.1196; found: 277.1195. HPLC-MS (M + H)^+^ = 277.2, *R*_t_ = 2.14 min (95%).

#### *N*-(4-(1*H*-Indazol-5-yl)­phenyl)-7*H*-pyrrolo­[2,3-*d*]­pyrimidin-4-amine (**92**)

The title compound was obtained by reaction of *N*-(4-iodophenyl)-7*H*-pyrrolo­[2,3-*d*]­pyrimidin-4-amine (**86**) (0.49 mmol, 164 mg), 5-(4,4,5,5-tetramethyl-1,3,2-dioxaborolan-2-yl)-1*H*-indazole (0.59 mmol, 144 mg), K_3_PO_4_ (2.36 mmol, 500 mg), Pd­(PPh_3_)_4_ (0.03 mmol, 35 mg) and a solution of dioxane/H_2_O/EtOH (4.5 mL). Yield: 25%. White solid. Mp 317–318 °C. ^1^H NMR (300 MHz, DMSO-*d*_6_) δ 13.08 (s, 1H), 11.78 (s, 1H), 9.40 (s, 1H), 8.31 (s, 1H), 8.12 (s, 1H), 8.04–7.98 (m, 3H), 7.72–7.66 (m, 3H), 7.65–7.57 (m, 1H), 7.25 (dd, *J* = 3.5, 2.3 Hz, 1H), 6.83 (dd, *J* = 3.5, 1.9 Hz, 1H). ^13^C NMR (75 MHz, DMSO-*d*_6_) δ 153.4, 150.8, 150.7, 139.3, 139.1, 134.4, 133.8, 132.6, 126.8 (2C), 125.4, 123.6, 122.2, 120.5 (2C), 117.5, 110.5, 103.7, 98.8. HRMS (ESI): calc. for C_19_H_14_N_6_ [M + H]^+^ 327.1353; found: 327.1355. HPLC-MS (M + H)^+^ = 327.1, *R*_t_ = 3.52 min (99%).

#### 3′-((7*H*-Pyrrolo­[2,3-*d*]­pyrimidin-4-yl)­amino)-(1,1′-biphenyl)-2-ol (**93**)

The title compound was obtained by reaction of *N*-(3-bromophenyl)-7*H*-pyrrolo­[2,3-*d*]­pyrimidin-4-amine (**87**) (1.04 mmol, 300 mg), 2-(4,4,5,5-tetramethyl-1,3,2-dioxaborolan-2-yl)­phenol (1.25 mmol, 274 mg), K_3_PO_4_ (4.16 mmol, 882 mg), Pd­(PPh_3_)_4_ (0.06 mmol, 86 mg) and a solution of dioxane/H_2_O/EtOH (4.5 mL). Yield: 82%. White solid. Mp 210–211 °C. ^1^H NMR (300 MHz, DMSO-*d*_6_) δ 11.74 (s, 1H), 9.49 (s, 1H), 9.32 (s, 1H), 8.27 (s, 1H), 7.99 (t, *J* = 1.9 Hz, 1H), 7.93 (ddd, *J* = 8.1, 2.2, 1.1 Hz, 1H), 7.35 (t, *J* = 7.9 Hz, 1H), 7.27 (dd, *J* = 7.6, 1.7 Hz, 1H), 7.23 (dd, *J* = 3.5, 2.3 Hz, 1H), 7.21–7.12 (m, 2H), 6.96 (dd, *J* = 8.1, 1.2 Hz, 1H), 6.89 (td, *J* = 7.4, 1.2 Hz, 1H), 6.82 (dd, *J* = 3.5, 1.8 Hz, 1H). ^13^C NMR (75 MHz, DMSO-*d*_6_) δ 154.3, 153.7, 150.9, 150.8, 140.0, 138.9, 130.3, 128.4, 128.0, 127.9, 123.1, 122.1, 121.1, 119.4, 118.7, 116.0, 103.7, 98.9. HRMS (ESI): calc. for C_18_H_14_N_4_O [M + H]^+^ 303.1240; found: 303.1241. HPLC-MS (M + H)^+^ = 303.2, *R*_t_ = 2.51 min (99%).

#### *N* ^3′^-(7*H*-Pyrrolo­[2,3-*d*]­pyrimidin-4-yl)-(1,1′-biphenyl)-2,3′-diamine (**94**)

The title compound was obtained by reaction of *N*-(3-bromophenyl)-7*H*-pyrrolo­[2,3-*d*]­pyrimidin-4-amine (**87**) (0.78 mmol, 225 mg), (2-aminophenyl)­boronic acid hydrochloride (0.93 mmol, 161 mg), Na_2_CO_3_ (1.95 mmol, 206 mg), Pd­(PPh_3_)_4_ (0.05 mmol, 54 mg) and a solution of dioxane/H_2_O/EtOH (4.5 mL). Yield: 21%. Gray solid. Mp 204–205 °C. ^1^H NMR (300 MHz, DMSO-*d*_6_) δ 11.75 (s, 1H), 9.37 (s, 1H), 8.27 (s, 1H), 7.96 (t, *J* = 1.9 Hz, 1H), 7.89 (ddd, *J* = 8.2, 2.2, 1.0 Hz, 1H), 7.41 (t, *J* = 7.9 Hz, 1H), 7.24 (dd, *J* = 3.5, 2.3 Hz, 1H), 7.11–7.01 (m, 3H), 6.81 (dd, *J* = 3.5, 1.9 Hz, 1H), 6.80–6.74 (m, 1H), 6.65 (td, *J* = 7.3, 1.2 Hz, 1H), 4.87 (s, 2H). ^13^C NMR (75 MHz, DMSO-*d*_6_) δ 153.5, 150.9, 150.8, 144.9, 140.6, 139.8, 129.8, 128.9, 128.2, 125.8, 122.3, 122.2, 120.5, 118.6, 116.7, 115.2, 103.8, 98.8. HRMS (ESI): calc. for C_18_H_15_N_5_ [M – H]^−^ 300.1255; found: 300.1251. HPLC-MS (M + H)^+^ = 302.2, *R*_t_ = 2.45 min (99%).

#### *N* ^3^-(7*H*-Pyrrolo­[2,3-*d*]­pyrimidin-4-yl)-(1,1′-biphenyl)-3,4′-diamine (**95**)

The title compound was obtained by reaction of *N*-(3-bromophenyl)-7*H*-pyrrolo­[2,3-*d*]­pyrimidin-4-amine (**87**) (0.69 mmol, 200 mg), (4-aminophenyl)­boronic acid hydrochloride (0.83 mmol, 143 mg), K_3_PO_4_ (3.45 mmol, 731 mg), Pd­(PPh_3_)_4_ (0.05 mmol, 54 mg) and a solution of dioxane/H_2_O/EtOH (4.5 mL). Yield: 49%. Beige solid. Mp 243–244 °C. ^1^H NMR (300 MHz, DMSO-*d*_6_) δ 11.73 (s, 1H), 9.29 (s, 1H), 8.28 (s, 1H), 7.97 (t, *J* = 1.9 Hz, 1H), 7.86 (ddd, *J* = 8.1, 2.2, 1.0 Hz, 1H), 7.41–7.27 (m, 3H), 7.23 (dd, *J* = 3.5, 2.3 Hz, 1H), 7.18 (ddd, *J* = 7.7, 1.8, 1.0 Hz, 1H), 6.79 (dd, *J* = 3.5, 1.9 Hz, 1H), 6.70–6.61 (m, 2H), 5.22 (s, 2H). ^13^C NMR (75 MHz, DMSO-*d*_6_) δ 153.6, 150.9, 150.8, 148.4, 141.1, 140.8, 128.8, 127.6, 127.1 (2C), 122.1, 119.3, 117.7, 117.3, 114.2 (2C), 103.7, 98.8. HRMS (ESI): calc. for C_18_H_15_N_5_ [M + H]^+^ 302.1400; found: 302.1401. HPLC-MS (M + H)^+^ = 302.2, *R*_t_ = 2.12 min (95%).

#### Synthesis of *N* ^4^-(7*H*-Pyrrolo­[2,3-*d*]­pyrimidin-4-yl)-(1,1′-biphenyl)-4,4′-diamine (**96**)

Compound **59** (0.90 mmol, 300 mg) and SnCl_2_·2H_2_O (2.25 mmol, 507 mg) were dissolved in a mixture of 1 mL of HCl 37% and 3 mL of ethanol. The reaction mixture was stirred under MW for 20 min at 120 °C. Afterward, the resulting solid was isolated by filtration, dissolved in H_2_O, and neutralized with 1 M NaOH. The white precipitate formed was subsequently isolated by filtration and dried under reduced pressure. Yield: 74%. White solid. Mp 199–200 °C. ^1^H NMR (300 MHz, DMSO-*d*_6_) δ 11.80 (s, 1H), 9.42 (s, 1H), 8.28 (s, 1H), 7.88 (d, *J* = 8.6 Hz, 2H), 7.53 (d, *J* = 8.7 Hz, 2H), 7.39 (d, *J* = 8.6 Hz, 2H), 7.24 (dd, *J* = 3.4, 2.3 Hz, 1H), 6.80 (dd, *J* = 3.4, 1.8 Hz, 1H), 6.69 (d, *J* = 8.5 Hz, 2H). ^13^C NMR (75 MHz, DMSO-*d*_6_) δ 153.3, 150.6, 150.4, 146.7, 138.2, 134.7, 128.2, 126.7 (2C), 125.4 (2C), 122.2, 120.9 (2C), 114.9 (2C), 103.6, 99.0. HRMS (ESI): calc. for C_18_H_15_N_5_ [M + H]^+^ 301.1327; found: 301.1317. HPLC-MS (M + H)^+^ = 302.2, *R*_t_ = 2.08 min (99%).

### Biology

#### Protein Expression and Purification

cDNA of human TTBK1 encoding for the kinase domain (residues 13–320) was cloned into a pSUMO-LIC vector (N-terminal His_6_ tag and 96-aa SUMO1 protein followed by a SUMO protease cleavage site). TTBK2 (residues 1–299) was cloned into a pNIC28-Bsa4 vector (N-terminal His_6_ tag followed by a TEV cleavage site). The plasmids were cotransformed into λ-phosphatase *Escherichia coli* BL21­(DE3)-R3-pRARE2. Precultures were grown at 37 °C overnight in Luria-Broth (LB) medium supplemented with kanamycin (50 mg/L) and chloramphenicol (34 mg/L). A 1% inoculum was transferred to Terrific-Broth (TB) medium (kanamycin, 50 mg/L), and cells were cultured at 37 °C with shaking until an OD_600_ of 1.8 was reached. At this point, the cultures were cooled to 18 °C, and protein expression was induced overnight with 0.5 mM IPTG when an OD_600_ of 2.8–3.0 was reached. Subsequently, cells were harvested and resuspended in a buffer containing 50 mM Tris pH 7.5, 500 mM NaCl, 10 mM imidazole, pH 7.0, 5% glycerol, and 1 mM TCEP, followed by cell lysis via sonication. The lysate was treated with 3.5 mL of 5% polyethylenimine (PEI) and clarified by centrifugation. The recombinant proteins were initially purified by Ni^2+^-affinity chromatography. Expression tags were removed via treating with sentrin-specific protease 1 (SENP1) for SUMO-tag cleavage (in TTBK1 purification) or TEV for His-tag cleavage (in TTBK2 purification). Then, the cleaved proteins were separated via reverse Ni^2+^-affinity chromatography and further purified by size-exclusion chromatography (SEC) on a HiLoad 26/600 Superdex-75pg column with a buffer containing 25 mM HEPES, pH 7.5, 250 mM NaCl, 10% glycerol, and 0.5 mM TCEP. Finally, the recombinant TTBK1 and TTBK2 proteins were concentrated to ∼8 and 5 mg/mL respectively.

#### *In Vitro* TTBK1 and TTBK2 Activity Assays

Kinase-Glo Luminescent Kinase Assay was obtained from Promega (Promega Biotech Iberica, SL). Human recombinant TTBK1 (13–320) and TTBK2 (1–299) were expressed in *E. coli* and purified using the protocol specified in the Protein Expression and Purification part. The specific CK1 family substrate peptide (RRKDLHDDEEDEAMSITA) was produced in the CIB Protein Chemistry service. ATP and all other reagents were from Sigma-Aldrich (St. Louis, MO). The assay buffer contained 40 mM Tris (pH 7.5), 20 mM MgCl_2_, 0.1 mg/mL BSA and 50 μM DTT. Kinase-Glo assays were performed in an assay buffer using white 96-well plates. In a typical assay, 10 μL of the CK1 peptide substrate at a final concentration of 0.12 μg/μL and 10 μL of the enzyme (15 ng) were added to each well followed by 10 μL of assay buffer containing the test compound (the test compounds were initially dissolved in DMSO at a concentration of 1 mM and subsequently diluted in assay buffer to the desired concentrations, ensuring that the final DMSO content in the reaction mixture did not exceed 1%) and 10 μL of ATP at 1 μM as final concentration. After 40 min incubation at room temperature, the enzymatic reaction was stopped with 40 μL of Kinase-Glo reagent. Glow-type luminescence (RLU) was recorded after 10 min using a microplate reader (GloMax Discover Microplate Reader, Promega Biotech Ibérica, SL). The enzymatic activity was determined using the difference of the RLU measured in the ATP-only control and the RLU of the corresponding sample. The inhibitory activities were calculated relative to the enzymatic activity measured in the absence of inhibitor (maximal activity control). The IC_50_ was defined as the concentration of each compound that reduces 50% of the enzymatic activity with respect to the maximal activity control.

#### Kinase Profiling

The kinase profiling studies were carried out by the MRC Phosphorylation Unit (University of Dundee) using a radioactive filter binding assay using ^33^P ATP and applying the appropriate protocol in any case.^40,41^

#### Parallel Artificial Membrane Permeability Assay (PAMPA) Blood–Brain Barrier (BBB)

Blood-brain barrier (BBB) compounds’ penetration was predicted using the parallel artificial membrane permeability assay (PAMPA).^42^ Ten commercial drugs of known BBB permeability (caffeine, enoxacin, hydrocortisone, desipramine, ofloxacin, piroxicam, testosterone, promazine, verapamil, and atenolol) were included in each experiment to validate the analysis set. Controls and test compounds (1–2 mg) were dissolved in 5 mL of assay buffer (7:3 v/v, PBS pH 7.4/EtOH). Samples were filtered (PDVF 20 μm filters, Symta) and UV absorbance was quantified by scanning from 220 to 400 nm (VarioskanTM, TermoFisher) to identify 2–5 optimal wavelengths. Once the initial absorbance of the samples was determined, 180 μL of each was added to the donor plate (MAIPS4510, Millipore), previously covered with 4 μL of porcine brain lipid (141101, Avanti Polar Lipids) dissolved in dodecane at 20 mg/mL. Then, the acceptor plate (141101, Millipore) was filled with 180 μL of assay buffer and the donor plate was stacked on top of the acceptor plate in a sandwich-like configuration for 2.5 h at room temperature. Following incubation, the donor plate was carefully removed, and the UV absorbance on the acceptor plate was recorded at the preselected wavelengths to determine the concentration of the compound that successfully crossed the lipid barrier. Every sample was analyzed in triplicate and results are given as the mean standard deviation (SD) of two independent experiments. Commercial drugs, PBS, ethanol, and dodecane were purchased from Sigma-Aldrich, Acros Organics, Merck, Aldrich, and Fluka.

#### Neuronal Cell Culture and Neuroprotection Assays

Human neuroblastoma SH-SY5Y cells were purchased from the European Collection of Cell Cultures (Health Protection Agency, Salisbury, UK), and were grown in DMEM (Gibco, ThermoFisher scientific) supplemented with 10% fetal bovine serum (FBS), and 1% penicillin/streptomycin under humidified 5% CO_2_ conditions at 37 °C. For cytotoxicity and neuroprotection assays, cells were seeded into 96-well plates at 40,000 cells/well. 24 h later, cells were treated with TTBK1 inhibitors at 1, 5, or 10 μM for cytotoxicity studies, and 1 μM, or the GSK3β inhibitor (Tideglusib, 5 μM) as a positive control for neuroprotection assays. Following 1 h, cells were exposed to ethacrynic acid at 80 μM (SML1083, Merck Life Science) for an additional 24 h only for neuroprotection tests. Cell viability was then assessed using the MTT assay or processed for Western blotting analysis. Cell survival was normalized to untreated controls and presented as percentage. The results represent the mean of *n* = 4 independent experiments.

#### MTT Assay

Cell viability was determined using 3-(4,5-dimethylthiazol-2-yl)-2,5-diphenyltetrazolium bromide (MTT) assay.^43^ Briefly, 20 μL of a solution of MTT in PBS (M5655, Merck Life Science) was added into each well to achieve a final concentration of 0.5 g/L. After 2 h of incubation (37 °C and 5% CO_2_) the culture medium was carefully removed, and formazan crystals were dissolved in 200 μL/well of DMSO. The optical density was measured at 560 nm using a microplate reader (GloMax Discover Microplate Reader, Promega Biotech Ibérica, SL). Cell viability was calculated as a percentage relative to the untreated control group defined as 100% of viability. Data come from the mean of *n* = 4 independent experiments.

#### Establishment and Culture of Patient-Derived Lymphoblastoid Cell Lines

Human lymphoblastoid cell lines (LCLs) were established from two FTD patients carrying a loss-of-function mutation in the progranulin gene and one healthy age-matched control (Table S12). Peripheral blood samples were obtained following written informed consent. All study protocols were approved by the Institutional Review Boards of Donostia Hospital (approval number CEI 18/004) and the Spanish National Research Council (CSIC) Institutional Review Board (date of approval Feb 1, 2018), in accordance with National and European Union Guidelines. LCLs were generated by infecting peripheral blood lymphocytes with the Epstein–Barr virus (EBV) as previously described.^44^ Cells were grown in suspension in upright T-flasks using RPMI-1640 medium (Gibco) supplemented with 10% (v/v) fetal bovine serum (FBS), and 1% penicillin/streptomycin. Cultures were maintained at 37 °C in a humidified atmosphere containing 5% CO_2_. For experimental assays, lymphoblasts were seeded at a density of 1 × 10^6^ cells/mL. Cells were treated with TTBK1 inhibitor **62** (5 μM), or the reference compound **TTBK1-IN-2** (5 μM) for 24 h. Following the incubation period, cells were harvested and processed for immunoblotting and immunofluorescence analysis.

#### Immunoblotting Analysis

Total protein extracts were obtained by cell lysis followed by centrifugation at 4,000 rpm for 10 min. Protein concentrations were determined using the Pierce BCA protein assay kit (Thermo Fisher). Equal amounts of proteins (20–40 μg) were separated by SDS–polyacrylamide gel electrophoresis and subsequently transferred onto poly­(vinylidene) fluoride (PVDF) membranes (Millipore). Membranes were then blocked with 5% Bovine Serum Albumin (BSA) (Sigma) and incubated overnight at 4 °C with the appropriate primary antibodies, listed in Table S4. After washing with Tris-buffered saline containing 0.1% Tween-20 (TBS-T), signals from the primary antibodies were amplified using species-specific antisera antibodies conjugated with horseradish peroxidase and immunoreactive bands were visualized with a chemiluminescent substrate detection system ECL (Bio-Rad). Relative band intensities were quantified using a ChemiDoc station with Quantity One 1D analysis software (Bio-Rad) and normalized to GAPDH and expressed as relative values. Data come from the mean of *n* = 4 independent experiments.

#### hTERT-RPE1 Ciliogenesis Studies

Human retinal pigment epithelial (RPE) cell line immortalized by stable expression of human telomerase reverse transcriptase (hTERT) were grown to confluence on coverslips using DMEM-F12 supplemented with 10% FBS. Medium was then replaced by DMEM-F12 + 0.5% FBS + 1% BSA containing the inhibitors at the indicated concentrations (compound **62** at 4 μM, and compound **TTBK1-IN-1** at 0.4 μM), or DMSO as control. After 24 h, cells were fixed and immunostained with ciliary markers ARL13B, and γ-tubulin, and with DAPI (Table S4). Cells were then imaged and the percentage of ciliated cells quantitated in each coverslip. Data represent the mean of *n* = 3 independent experiments.

#### Immunofluorescence Analysis

To facilitate the adhesion of nonadherent lymphoblasts, coverslips were coated with 0.25% gelatin (Sigma, Madrid, Spain) for 30 min at room temperature, followed by 1 mg/mL poly-l-lysine (Sigma, Madrid, Spain) diluted 1:50 in Borax buffer (Na_2_B_4_O_7_·10H_2_O 15 mM, pH 8.4) overnight at 37 °C. After treatment with the compounds, cells were fixed with 4% paraformaldehyde in PBS for 20 min and permeabilized with 0.1% Triton X-100 for 10 min. Samples were blocked with 1% BSA in PBS for 90 min at 37 °C and subsequently incubated overnight at 4 °C with the primary antibody against TDP-43 (Table S4). After washing with PBS, cells were incubated with the corresponding Alexa Fluor-conjugated secondary antibody (1:1000) for 1 h at 37 °C. Nuclei were counterstained with DAPI (1:1000). Preparations were mounted using FluorSave reagent (Calbiochem, Madrid, Spain). High-resolution confocal images were acquired using a Leica TCS SP5 microscope equipped with a 100× oil immersion objective. Image analysis was performed using ImageJ software (version 1.53K). To quantify TDP-43 distribution, the nuclear/cytoplasmic ratio was determined by measuring the fluorescence intensity in the nucleus (DAPI-positive) and cytoplasmic compartments in at least four independent fields of view per condition.

#### Cardiovascular Safety Assays

Cardiovascular safety was evaluated using fluorescence-based high-throughput screening (HTS) in HEK293 cell lines stably expressing human Cav1.2, Nav1.5, or hERG channels in Fundacion Medina. L-type calcium channel activity was assessed using a Cav1.2/Kir2.3 coexpression model. Calcium influx is triggered by buffer addition that increases calcium concentration to 2 mM while maintaining the potassium concentration at 25 mM. Changes in intracellular calcium are monitored using a calcium-sensitive fluorescent dye Fluo-4 (Invitrogen) in a FLIPR TETRA plate reader (Molecular Devices), with israpidine (250 nM) as a positive control. Sodium channel (Nav1.5) assays utilized the FLIPR Membrane Potential Assay dye, using veratridine to maintain the open-state and tetrodotoxin (50 μM) as a positive control. hERG potassium channel inhibition was determined via the FluxOR potassium channel assay, with astemizole (10 μM) as a standard. In all assays, compounds were tested in a 10-point dose–response curve (50 μM top concentration, 1:2 dilution) and in triplicate. Fluorescence kinetics were monitored using a FLIPR TETRA system, and IC_50_ values were calculated using Genedata Screener. All measurements were performed at room temperature after a 30 min incubation to allow equilibration of the test compounds.

#### Genotoxicity Studies

The mutagenic potential was evaluated using Ames fluctuation assay (Ames MPFTM 98/100 Microplate Format Mutagenicity Assay, from Xenometrix AG; Allschwill, Switzerland) against *Salmonella typhimurium* strains TA98 and TA100 in Fundación Medina. The evaluation was performed both in the presence and absence of an exogenous metabolic activation system (S9) derived from Aroclor 1254-induced rat liver. The test compound was dissolved in DMSO and tested at four concentrations of 6.25, 12.5, 25, and 50 μM. Briefly, bacteria were incubated 90 min in medium containing the test compound or controls, and limited amount of histidine. After incubation, the cultures were diluted in pH indicator medium lacking histidine and aliquoted into a 384-well plate. Within 48 h at 37 °C, mutagenicity was determined by monitoring the pH-dependent color change from purple to yellow caused by catabolic activity of revertant colonies. All experiments were performed in triplicate with DMSO as negative control (0.5% v/v). The results were compared with three different mutagenic positive controls: 2-nitrofluorene (2-NF) for TA98 without S9 mix, 4-nitroquinoline-*N*-oxide (4-NQO) for TA100 without S9 mix, and 2-aminoanthracene (2-AA) for both TA98 and TA100 with S9 mix. A dose-dependent increase in the number of revertant colonies upon exposure to the chemical compared to the controls indicates that the sample is mutagenic in the Ames MPF 98/100 assay.

#### *In Vivo* Pharmacokinetic Studies

The study was conducted in accordance with the guidelines of the Institutional Animal Ethics Committee (IAEC) and approved by Sai Life Sciences (Hinjewadi, Pune, India) (no. SAIDMPK/PK-24–01–0104, February 2024). Healthy male BALB/c mice (8–12 weeks old, 17–30 g) were used. The animals were divided into two groups (*n* = 24 per group) with three mice per time point design. Group 1 received a 5 mg/kg dose of the compound administered intraperitoneally (i.p.) as a solution formulation (5% NMP, 5% Solutol HS-15, and 90% normal saline). Group 2 was administered a 10 mg/kg through oral route (p.o.) as a hazy uniform suspension (0.5% Tween 80 and 0.5% Na-CMC in RO water). Blood samples (∼60 μL) and brain tissue were collected from three mice per time point at 0.083 (for i.p. only), 0.25, 0.5, 1, 2, 4, 6 (for p.o. only), 8 and 24 h. Following collection, brain and plasma samples were processed for analysis by protein precipitation method and analyzed with fit-for-purpose LC-MS/MS method (LLOQ = 2.00 ng/mL for both plasma and brain). Pharmacokinetic parameters were calculated through noncompartmental analysis using Phoenix WinNonlin (ver. 8.0).

#### Metabolic Profile

The formation of the main metabolites of compound **62** was evaluated in incubations with human, rat, minipig, and dog liver microsomes (HLM, RLM, MPLM, and DLM, respectively) at Fundación Medina. The metabolic reactions were initiated by adding the liver microsomal protein solution (final concentration 1 mg/mL) to an equal volume of buffer containing the test compound **62** (1 μM) and cofactors NADPH (1.3 mM). The total incubation volume was 400 μL. Control incubations without NADPH were also performed to assess NADPH-independent metabolism and/or chemical instability in the incubation buffer. Verapamil was included as a positive control to monitor metabolic activity. Reactions were terminated by the addition of 60 μL of ice-cold MeCN, followed by centrifugation at 3,700 rpm for 15 min. Samples were analyzed by LC-HRMS in positive ionization mode using an Agilent 1290 Infinity UHPLC system coupled to a SCIEX TripleTOF 5600 mass spectrometer, and data were processed using MetabolitePilot software. LC-HRMS analysis was performed using TOF MS with IDA MS/MS acquisition in combination with an Atlantis T3 C18 column (2.1 mm × 150 mm, 3 μm). The mobile phase consisted of H_2_O and MeCN with 0.1% formic acid, at a flow rate of 0.3 mL/min. The column temperature was maintained at 30 °C, with an injection volume of 3 μL and a total run time of 20 min.

#### Maximum Tolerated Dose (MTD) Studies

The MTD study was carried out in Sprague–Dawley rats following oral gavage administration for 7 consecutive in SaiLife Sciences. The experimental protocol consisted of a toxicity phase comprising four groups: one control (G1) and three treatment groups (G2–G4) receiving treatment at doses of 10, 50, and 100 mg/kg/day, respectively (*n* = 3 per sex/group). For the toxicokinetic (TK) phase, three satellite groups (G2TK–G4TK) were maintained (*n* = 3 per sex/group). Control animals (G1) were administered the vehicle (0.1% v/v Tween 80 and 0.5% aqueous HPMC). In all groups, the dosing volume was maintained at a constant 10 mL/kg/day. Rats from toxicokinetic groups G2TK to G4TK were subjected to plasma drug concentration determination on day 1 and 7 at 0 (only on day 7), 0.5, 1, 2, 4, 8, and 24 h postdose administration. Parameters evaluated during the study included in-life observations such as clinical signs observation, body weights, percent body weight gains, feed consumption, hematology, clinical chemistry, toxicokinetic, gross pathology, organ weights, and histopathology.

#### X-ray Data Collection and Structure Determination

Co-crystallization trials were performed using the sitting-drop vapor-diffusion method at 293 K with a mosquito crystallization robot (TTP Labtech, Royston UK). The concentration of TTBK1 protein used for crystallization was 9.9 mg/mL and for TTBK2 was 8.8 mg/mL (measured using Nanodrop). Prior to crystallization trials, proteins were incubated for 1 h with compound **62** at a final concentration of 1 mM with a DMSO concentration of 2%. The crystallization plates were set up using 200 nL drops with three different Prot+Lig/Reservoir ratios (2:1, 1:1 or 1:2) for each crystallization condition. TTBK1 crystals grew in a condition containing 20% PEG 3350, 0.2 M sodium acetate, and 0.1 M Tris, pH 8.0, while TTBK2 crystals grew in a condition containing 25% PEG 3350, 0.2 M ammonium acetate, and 0.1 M HEPES, pH 7.5. Crystals were cryo-protected with mother liquor supplemented with 20% ethylene glycol and flash-frozen in liquid nitrogen. The crystals of compound **62** with both TTBK1 and TTBK2 grew in the 1:2 ratio drops at the above-mentioned crystallization condition, corresponding to a final DMSO concentration of 0.7% in the crystallization drop. The growing temperature was 293 K for both proteins.

X-ray data sets were collected at 100 K at beamlines I03 (TTBK1) and I04 (TTBK2) of the Diamond Light Source (DLS), Didcot, UK. Diffraction data for both crystals were integrated with autoPROC^45^ and scaled with AIMLESS.^46^ Structures were solved by molecular replacement using MOLREP^47^ with PDB entry 7Q8W for TTBK1 and 7Q8Y for TTBK2 as a starting model. The structures were then refined through iterative manual model building with COOT^48^ and refinement with PHENIX.^49^ The accuracy of the final models was validated using MolProbity.^50^ The data collection and refinement statistics are summarized in Supporting Information Table S3.

#### Computational Protocol

Extended molecular dynamics (MD) simulations were conducted to characterize the structural stability and dynamic behavior of the simulated systems. The crystallographic structures of TTBK1 and TTBK2 bound to compound **62** were used as initial models (PDB IDs 31HO and 31HP, respectively). Each protein–ligand complex was embedded in a pre-equilibrated octahedral box of TIP3P water molecules.^51^ Ionizable residues were assigned their standard protonation states corresponding to physiological pH. The final solvated systems contained the protein–ligand complex, approximately 12,000 water molecules, and 12 chloride ions, resulting in a total system size of approximately 41,000 atoms. The systems were equilibrated using the NPT ensemble, while production simulations were performed under NVT conditions. Periodic boundary conditions were applied throughout the simulations, and long-range electrostatic interactions were treated using the particle mesh Ewald method with a grid spacing of 1 Å. All simulations were carried out using Amber24.^52^ The Amber ff14SB force field^53^ was used to describe the protein, while ligand parameters were generated using the GAFF force field.^54^ Restrained electrostatic potential-fitted (RESP)^55^ partial atomic charges were derived from B3LYP/6–31G­(d)^56^ quantum mechanical calculations. Prior to equilibration, each system was subjected to a stepwise energy minimization protocol. First, the positions of protein hydrogen atoms were optimized using 2,000 cycles of steepest descent followed by 8,000 cycles of conjugate gradient minimization. The same minimization strategy was then applied to the solvent molecules and counterions. Finally, all atoms in the system were minimized using 4,000 cycles of steepest descent followed by 1,000 cycles of conjugate gradient minimization. Equilibration was performed in six sequential stages. The system was initially heated from 0 to 100 K over 20 ps under NVT conditions. This was followed by four NPT thermalization steps, each lasting 50 ps, to gradually increase the temperature from 100 to 300 K. A final 5 ns equilibration step was then performed at 300 K and 1 atm to ensure density stabilization. The equilibrated structures were subsequently used as the starting conformations for the production MD simulations. During equilibration, distance restraints were applied to preserve the initial ligand binding pose and minimize artificial displacement at the beginning of the simulations. These restraints were gradually reduced and fully removed during the early stages of the production runs.^57,58^ Each production MD simulation was continued for 700 ns.

##### Essential Dynamics

Essential dynamics (ED) analysis was performed to determine the principal collective motions associated with the conformational changes sampled during the MD simulations. This method enables the motions along individual modes to be examined and visualized independently, thereby facilitating the identification of the dominant large-scale movements of the protein. For this purpose, a positional covariance matrix was constructed and diagonalized to obtain the collective deformation modes, represented by eigenvectors. The corresponding eigenvalues describe the relative contribution of each mode to the overall structural variance. In the present study, ED analysis was performed on 20,000 snapshots extracted from each 700 ns trajectory, considering only backbone atoms. The calculations were carried out using PCAsuite, implemented within the pyPCcazip toolkit.^59^

#### *In Vivo* CaMKIIα-TDP43 Mouse Model

The *in vivo* efficacy of the selective TTBK1 inhibitor was evaluated in FVB/N-Tg (CaMKIIα-TDP43) mouse model previously described by Tsai et al.^37^ These mice overexpress human TDP-43 selectively in forebrain regions under the control of the CaMKIIα promoter, resulting in increased TDP-43 levels in the medial prefrontal cortex and hippocampus. Animals were housed in the animal facility of the Complutense University of Madrid (CAI-Animalario, Faculty of Medicine, ref ES280790000086) under controlled temperature (22 ± 1 °C) and photoperiod conditions (12 h light/dark cycle), with ad libitum access to food and water. All experimental procedures were performed in accordance with European legislation (Directive 2010/63/EU), ARRIVE guidelines, and institutional regulations, and were approved by the corresponding ethics committees and regulatory authorities (PROEX 059/16).

Male transgenic mice and wild-type (WT) littermates (*n* = 10–12 per group) received daily intraperitoneal (i.p.) administrations of compound **62** from postnatal day (PND) 45 to PND90. Dose selection was based on previous pharmacokinetic studies.

Recognition memory was evaluated using the novel object recognition (NOR) test, which was performed at PND60 and PND90, as previously described in Santos García.^60^ Mice were tested in an opaque methacrylate arena (50 cm × 50 cm × 50 cm) containing fresh bedding material. During the training session, animals were allowed to explore two identical objects. In the test session, one familiar object was replaced by a novel object, and exploratory behavior was recorded. Memory performance was assessed using the discrimination index [(time exploring the novel object-time exploring the familiar object)/total exploration time], expressed as percentages.

Following euthanasia, the brains were rapidly removed, and the hemispheres were separated. Immunofluorescence analyses were performed using antibodies against CITP2, GFAP, Iba1, and S100β. For quantification, images were acquired under identical exposure and illumination conditions and analyzed using ImageJ/Fiji software (NIH, Bethesda, MD, USA). At least six images per animal were analyzed for each experimental condition.

Brain tissue samples were homogenized in RIPA buffer supplemented with protease and phosphatase inhibitor cocktails (Roche, Mannheim, Germany). Protein concentration was determined using the Pierce BCA Protein Assay Kit (Thermo Fisher Scientific, Madrid, Spain). Equal amounts of protein were separated by SDS-PAGE using TGX Stain-FreeTM gels (Bio-Rad Laboratories, Hercules, CA, USA) and transferred onto PVDF membranes (Millipore, Bedford, MA, USA). Membranes were incubated with TDP-43 and phospho-TDP-43 antibodies, followed by species-specific horseradish peroxidase-conjugated secondary antibodies. Immunoreactive bands were visualized using enhanced chemiluminescence (ECL, Bio-Rad, Madrid, Spain) and quantified using a ChemiDoc imaging system with Quantity One software (Bio-Rad Laboratories). Protein levels were normalized to total protein staining and expressed as a percentage relative to WT vehicle-treated animals.

#### Statistical Analysis

Statistical analyses were performed with Graph Pad Prism (Version 8.0). Data are presented as mean ± standard error of the mean (SEM). Normality was checked with the Shapiro–Wilk test. Parametric tests were therefore used in the statistical analysis. Significant differences between groups were evaluated by using analysis of variance (ANOVA), followed by the corresponding multiple comparison test. The value of *p* < 0.05 was considered significant.

## Supplementary Material









## Data Availability

The coordinates and structure factors of TTBK1 and TTBK2 in complex with compound **62** have been deposited in the Protein Data Bank (PDB) under accession numbers 31HO and 31HP, respectively.

## Notes

Molecular strings (CSV)
Comparative SAR of *para*- and *meta-* orientated biphenyls analogues; PAMPA-BBB assay effective Permeability *P*_e_ values; X-ray data collection and refinement statistics; list of antibodies used; kinase panel profiling for **62**; Ion channel inhibition of **62**; results of AMES test for inhibitor **62**; metabolites of **62** after microsomes treatment; demographic characteristics of FTD and control subjects; dose–response curve of **TTBK1-IN-1** against TTBK1 and TTBK2 kinase domain using Kinase-Glo assay; cell viability of SH-SY5Y with TTBK1 inhibitors; total TDP-43/GAPDH quantification plot; *p*-TDP-43 levels in SH-SY5Y cells after EA insult with and without compound **62** treatment; crystals and X-ray structures of **62** with the human TTBK1 and TTBK2 kinase domain; TTBK1 activity in hTERT-RPE1 cells; HPLC chromatograms, and ^1^H and ^13^C NMR spectra of compound **62**, chemical procedures to obtain compounds **2**–**42** and **85**–**87** (PDF)
TTBK1-comp **62** (PDB)
TTBK2-comp **62** (PDB)

## 1

Institut Químic de Sarrià (IQS), Universitat Ramon Llull, Barcelona 08017, Spain

## 2

Universidad Alfonso X El Sabio, Madrid, Spain.

## 3

Technological University Dublin (TUD).

## Abbreviations Used

ALS: amyotrophic lateral sclerosis
ATP: adenosine triphosphate
BBB: blood-brain-barrier
CNS: central nervous system
DMSO: dimethyl sulfoxide
EA: ethacrynic acid
ED: essential dynamics
FL: full-length
FTD: frontotemporal dementia
HBA: hydrogen-bond acceptor
HBD: hydrogen-bond donor
IC_50_: half-maximal inhibitory concentration
i.p.: intraperitoneally
Kp: brain-to-plasma ratios
LATE: limbic-predominant age-related TDP-43 encephalopathy
LCLs: lymphoblastoid cell lines
MeCN: acetonitrile
MD: molecular dynamics
Mp: melting point
MTD: maximum tolerated dose
MW: microwave
NMR: nuclear magnetic resonance
NOR: novel object recognition
PAMPA: parallel artificial membrane permeability assay
*P*_e_: effective permeability
PND: postnatal day
p.o.: orally (*per os*)
r.t.: room temperature
SD: standard deviation
SEM: standard error of the mean
S_N_Ar: nucleophilic aromatic substitution
THF: tetrahydrofuran
TTBK1: tau tubulin kinase 1
TTBK2: tau tubulin kinase 2
TDP-43: transactive response DNA-binding protein of 43 kDa
SI: selectivity index
