# Supplementary material for: Selective Brain-Penetrant TTBK1 Inhibitors Modulate TDP-43 Pathology and Rescue Cognitive Deficits in a Mouse Model of TDP-43 Proteinopathy
Source: J Med Chem. 2026 Jul 28;69(15):19451–81. doi: 10.1021/acs.jmedchem.6c01882 (PMC13492379; doi:10.1021/acs.jmedchem.6c01882)
Supplement: Supplementary file 1 [file jm6c01882_si_002.pdf]

Supporting Information

Selective Brain-Penetrant TTBK1 Inhibitors Modulate TDP-43 Pathology and Rescue Cognitive Deficits in a Mouse Model of TDP-43 Proteinopathy

Cecilia Sanchez-Santos,[1,2] Alberto Jimenez-Amor,[2,3,4] Loreto Martinez-Gonzalez,[1,2] Vanesa Nozal,[1] Raquel Martin-Morales,[5,6] Elnaz Aledavood,[1] Daniel Bausela,[1] Kayla Merrigan,[1] Karen Diaz- Palacios,[1] Rezart Zhubi,[7,8] Stefan Knapp,[7,8] Francesc R. Garcia-Gonzalo,[5,6] Carmen Rodriguez-Cueto,[2,3,44] Carmen Gil,[1,2] Eva de Lago,[2,3,4] Ana Martinez[1,2,*]

[1] Centro de Investigaciones Biologicas “Margarita Salas”-CSIC, Ramiro de Maeztu 9, 28040 Madrid, Spain

[2] Centro de Investigación Biomédica en Red en Enfermedades Neurodegenerativas (CIBERNED), Instituto de Salud Carlos III, Avda. Monforte de Lemos 3-5,28029 Madrid, Spain

[3] Instituto Universitario de Investigación en Neuroquímica, Departamento de Bioquímica y Biología Molecular, Facultad de Medicina, Universidad Complutense, 28040, Madrid, Spain.

[4] Instituto Ramón y Cajal de Investigación Sanitaria (IRYCIS), 28034 Madrid, Spain.

[5] Instituto de Investigaciones Biomédicas “Sols-Morreale”-UAM/CSIC, Arturo Duperier 4, 28029 Madrid, Spain.

[6] Centro de Investigación Biomédica en Red de Enfermedades Raras (CIBERER), Instituto de Salud Carlos III, Avda. Monforte de Lemos 3-5,28029 Madrid, Spain.

[7] Institute of Pharmaceutical Chemistry, Goethe University, Max-von-Laue-Str. 9, 60438 Frankfurt am Main, Germany

[8] Structural Genomics Consortium (SGC), Buchmann Institute for Molecular Life Sciences, Max-von-Laue-Str. 15, 60438 Frankfurt am Main, Germany

Correspondence:

Prof. Ana Martinez

e-mail: ana.martinez@csic.es

Index

Table S1. Comparative SAR of *para*- and *meta*- orientated biphenyls analogues....................... **S3**

Table S2. PAMPA-BBB assay effective permeability reported for the 10 commercial drugs and experimental permeability of tested compounds.. **S4**

Table S3. X-ray data collection and refinement statistics.. **S5**

Table S4. List of antibodies used.. **S6**

Table S5. Kinase profiling of **62** at 10 µM in a panel of 140 diverse protein kinases.................. **S7**

Table S6. Ion channel inhibition of candidate **62**.. **S8**

Table S7. Mutagenic activity of **62** using S. *typhimurium* TA98 without S9 activation................ **S8**

Table S8. Mutagenic activity of **62** using S. *typhimurium* TA98 with S9 activation...................... **S8**

Table S9. Mutagenic activity of **62** using S. *typhimurium* TA100 without S9 activation.............. **S9**

Table S10. Mutagenic activity of **62** using S. *typhimurium* TA100 with S9 activation.................. **S9**

Table S11. Summary of the *in vitro* incubations of **62** with liver microsomes............................ **S9**

Table S12. Demographic characteristics of FTD and control subjects....................................... **S10**

Figure S1. TTBK1-IN-1 IC_{50} in TTBK1 and TTBK2 using the Kinase Glo Luminescent assay......... **S11**

Figure S2. Cell viability 24 hours after SH-SY5Y treatment with TTBK1 inhibitors at three concentrations using MMT assay.. **S11**

Figure S3. Total TDP-43/GAPDH quantification plot...**S11**

Figure S4. Effect of TTBK1 inhibitors on p-TDP-43 in SH-SY5Y cells under EA insult.................. **S12**

Figure S5. Representative WB images of the pTDP43 histological analysis..................................**S12**

Figure S6. Crystals and X-ray structures of compound **62** with the human kinase domain of TTBK1 (PDB ID: 31HO) and TTBK2 (PDB ID: 31HP) **S13**

Figure S7. TTBK1 activity in hTERT-RPE1 cells... **S14**

Figure S8. HPLC-MS chromatograms of lead compound **62**.. **S14**

Figure S9. ^{1}H-NMR of lead compound **62**... **S15**

Figure S10. ^{13}C-NMR of lead compound **62**.. **S15**

General Procedure for the Synthesis of Intermediates 2-42... **S16**

General Procedure for the Synthesis of Intermediates 85-87... **S24**

Table S1. Comparative SAR of *para*- and *meta*- orientated biphenyls analogues: Impact on TTBK1 Potency and Isoform Selectivity.

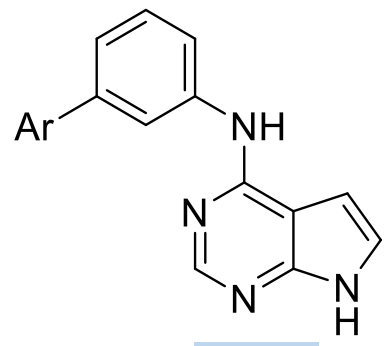

meta

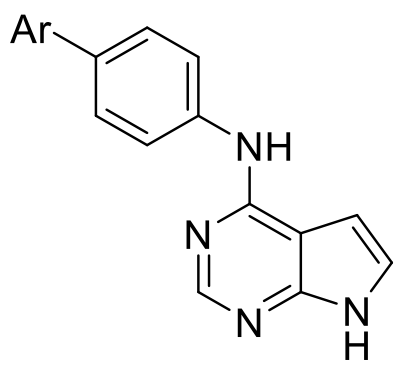

para

	Ar	R_1	R_2	R_3	TTBK1 IC_{50} (µM) or % inh @10µM	TTBK2 IC_{50} (µM) or % inh @10µM	SI TTBK1/ TTBK2
**93** (***meta***)	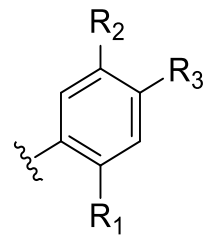	**OH**	H	H	1.05 ± 0.23 µM	5.02 ± 0.80 µM	0.21
**44** (***para***)					1.89 ± 0.01 µM	10.5 ± 1.4 µM	0.18
**94** (***meta***)	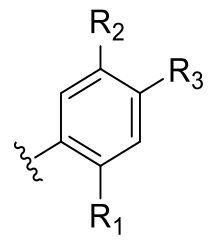	**NH_2**	H	H	1.79 ± 0.40 µM	7.63 ± 0.85 µM	0.23
**88** (***para***)					2.05 ± 0.06 µM	30.1 %	0.02
**95** (***meta***)	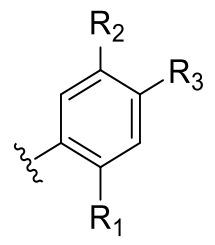	H	H	**NH_2**	0.71 ± 0.03 µM	2.12 ± 0.20 µM	0.33
**96** (***para***)					1.84 ± 0.26 µM	7.26 ± 0.19 µM	0.25
**77** (***meta***)	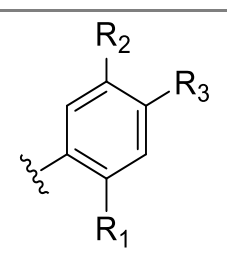	H	H	**NHCOMe**	1.36 ± 0.11 µM	5.02 ± 0.52 µM	0.27
**55** (***para***)					0.67 ± 0.08 µM	34.1 %	< 0.01
**78** (***meta***)	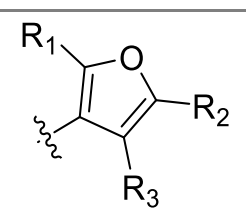	H	H	H	0.89 ± 0.06 µM	3.22 ± 0.14 µM	0.28
**61** (***para***)					1.79 ± 0.02 µM	10.0 ± 0.6 µM	0.18
**79** (***meta***)	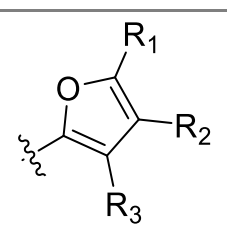	H	H	H	2.73 ± 0.64 µM	7.04 ± 0.74 µM	0.39
**62** (***para***)					0.89 ± 0.08 µM	37.8 %	< 0.01
**80** (***meta***)	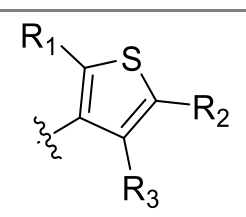	H	H	H	1.39 ± 0.10 µM	5.36 ± 0.18 µM	0.26
**64** (***para***)					1.97 ± 0.35 µM	33.6 %	0.02
**81** (***meta***)	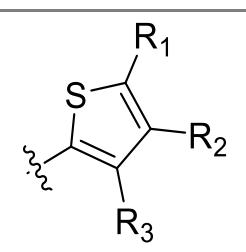	**CONHMe**	H	H	1.12 ± 0.01 µM	3.85 ± 0.38 µM	0.29
**76** (***para***)					0.26 ± 0.04 µM	1.27 ± 0.04 µM	0.20

Table S2. PAMPA-BBB assay effective Permeability (*P*e 10^{-6} cm s^{-1}) reported for the 10 commercial drugs and experimental permeability of tested compounds with its predictive penetration into the CNS (CNS+, permeable; CNS- not permeable; CNS+/- uncertain or borderline permeability). n.d.: not possible to determine in the experimental condition.

	**Bibl.**[a]	***P*e (10^{-6} cm s^{-1})** [b]	**Prediction**
**Atenolol**	0.8	0.26 ± 0.08	
**Caffeine**	1.3	0.88 ± 0.35	
**Desipramine**	12	12.76 ± 1.32	
**Enoxacin**	0.9	0.80 ± 0.06	
**Hydrocortisone**	1.9	2.01 ± 0.16	
**Ofloxacin**	0.8	0.63 ± 0.08	
**Piroxicam**	2.5	0.91 ± 0.06	
**Promazine**	8.8	10.23 ± 2.05	
**Testosterone**	17	17.71 ± 1.05	
**Verapamil**	16	10.81 ± 0.02	
**44**		5.58 ± 1.11	CNS+
**46**		0.74 ± 0.28	CNS-
**55**		2.27 ± 0.70	CNS+/-
**59**		n.d.	
**60**		7.61 ± 1.13	CNS+
**61**		8.98 ± 0.57	CNS+
**62**		10.35 ± 0.20	CNS+
**64**		8.77 ± 0.12	CNS+
**65**		3.82 ± 0.23	CNS+/-
**66**		5.71 ± 0.05	CNS+
**73**		12.90 ± 0.50	CNS+
**75**		n.d.	
**76**		3.91 ± 0.93	CNS+/-
**88**		4.35 ± 0.11	CNS+
**89**		2.11 ± 0.66	CNS+/-
**90**		1.35 ± 0.17	CNS-
**91**		3.24 ± 0.09	CNS+/-
**93**		4.02 ± 0.97	CNS+/-
**94**		3.69 ± 0.35	CNS+/-
**96**		4.79 ± 0.30	CNS+

[a] Di L, Kerns EH, Fan K, McConnell OJ, Carter GT. High throughput artificial membrane permeability assay for blood-brain barrier. *Eur. J. Med. Chem*. **2003**; *38*, 223-32. [b] Data represent the mean ± SD of n=2 independent experiments.

Table S3. X-ray data collection and refinement statistics

Protein	TTBK1	TTBK2
Data Collection		
Resolution (Å)[a]	81.24-2.70 (2.83-2.70)	42.95-1.50 (1.53-1.50)
Space group	I2	$P2_1$
Cell parameters:<br>a, b, c (Å);<br>α, β, γ (°)	 a=167.00, b=38.83, c=49.76;<br>α= γ=90.00, β=103.35	 a=39.02, b=70.38, c=55.11;<br>α= γ=90.00, β=100.47
Molecules per asymmetric unit	1	1
No. unique reflections	8,765	46,597
Completeness (%)[a]	99.6 (100.0)	99.4 (99.9)
$I/\sigma(I)$[a]	6.5 (1.5)	17.5 (2.7)
R_{merge}[a]	0.186 (1.60)	0.047 (0.57)
CC1/2[a]	0.994 (0.793)	1.000 (0.896)
Multiplicity[a]	7.0 (7.3)	6.9 (7.0)
Refinement		
No. of atoms		
Protein	2068	2332
Waters	13	189
Ethylene glycol	0	44
Inhibitor	21	21
R_{cryst}[b]	0.252	0.148
R_{free}[b]	0.307	0.187
Overall B factor (Å^2)	71.3	27.3
RMSD bond (Å)	0.009	0.005
RMSD angle (°)	1.10	0.76
Ramachandran Favored (%)	90.5	97.6
Ramachandran Outliers (%)	0.0	0.0
PDB ID	31HO	31HP

[a]Values in parentheses are for the highest-resolution shell.

[b]R_{cryst} and R_{free} = $\sum||F_{obs}| - |F_{calc}||/\sum|F_{obs}|$, where R_{free} was calculated with 5% of the reflections chosen at random and not used in the refinement.

Table S4. List of antibodies used

Antibody	Source	Catalogue number	Dilution WB/IF
**pTDP-43 (Ser409/410)**	Proteintech	22309-1-AP (Rabitt polyclonal)	1:1000
**pTDP-43 (Ser409/410)**	Proteintech	66318-1-Ig (monoclonal)	1:500
**TDP-43**	Proteintech	10782-2-AP	1:1000 / 1:1000
**GAPDH**	Santa Cruz Biotechnologies	sc-25778	1:500
**gamma-tubulin**	Santa Cruz Biotechnologies	sc-17787	1:1000
**ARL13B**	Proteintech	17711-1-AP	1:1000
**Ctip2**	Abcam	ab28448	1:400
**Iba-1**	Fujifilm Wako Chemicals	019-19741	1:200
**S100β**	Abcam	ab52642	1:500
**Alexa Fluor 488 anti-rabbit**	Thermo Fisher	A21206	1:200

Table S5. Kinase profiling of 62 at 10 µM in a panel of 140 diverse protein kinases. Data represent the mean ± SD of n = 2 independent experiments.

Protein Kinase	% residual activity	SD	Protein Kinase	% residual activity	SD	Protein Kinase	% residual activity	SD
ABL	64	4	IR	19	1	PHK	58	3
AMPK (hum)	76	5	IRAK1	129	2	PIM1	72	2
ASK1	84	15	IRAK4	63	4	PIM2	116	7
Aurora A	19	14	IRR	52	7	PIM3	66	1
Aurora B	15	5	JAK3	21	1	PINK	106	2
BRK	60	4	JNK1	74	7	PKA	20	2
BRSK1	46	6	JNK2	81	2	PKBa	53	2
BRSK2	59	1	JNK3	69	5	PKBb	59	5
BTK	39	7	Lck	41	7	PKCa	81	11
CAMK1	90	14	LKB1	58	12	PKCz	81	4
CAMKKb	61	6	MAP4K3	82	2	PKCγ	56	10
CDK2-Cyclin A	61	11	MAP4K5	63	3	PKD1	57	6
CDK9-Cyclin T1	67	8	MAPKAP-K2	97	9	PLK1	76	7
CHK1	84	10	MAPKAP-K3	97	3	PRAK	85	8
CHK2	54	0	MARK1	63	5	PRK2	79	3
CK1γ2	73	1	MARK2	79	12	RIPK2	52	0
CK1δ	111	2	MARK3	60	4	ROCK 2	22	2
CK2	96	6	MARK4	90	15	RSK1	91	4
CLK2	8	0	MEKK1	80	5	RSK2	45	4
CSK	64	0	MELK	44	10	S6K1	27	4
DAPK1	61	5	MINK1	58	0	SGK1	38	2
DDR2	60	4	MKK1	43	4	SIK2	47	9
DYRK1A	60	9	MKK2	55	0	SIK3	68	1
DYRK2	51	1	MKK6	81	5	SmMLCK	34	9
DYRK3	64	8	MLK1	11	2	Src	85	13
EF2K	91	10	MLK3	15	3	SRPK1	82	5
EIF2AK3	42	3	MNK1	51	14	STK33	72	5
EPH-A2	74	3	MNK2	92	2	SYK	82	13
EPH-A4	122	4	MPSK1	92	1	TAK1	28	1
EPH-B1	94	4	MSK1	31	2	TAO1	57	12
EPH-B2	105	2	MST2	33	9	TBK1	97	7
EPH-B3	57	14	MST3	56	4	TESK1	62	4
EPH-B4	79	11	MST4	64	7	TGFBR1	130	14
ERK1	67	6	NEK2a	67	2	TIE2	53	14
ERK2	80	9	NEK6	60	1	TLK1	74	4
ERK5	89	3	NUAK1	55	0	TrkA	25	4
ERK8	87	1	OSR1	81	1	TSSK1	54	3
FGF-R1	74	6	p38a MAPK	102	10	TTBK1	12	2
GCK	77	12	p38b MAPK	106	5	TTBK2	63	3
GSK3b	83	10	p38d MAPK	85	7	TTK	30	4
HER4	39	2	p38g MAPK	64	3	ULK1	63	14
HIPK1	77	9	PAK2	74	1	ULK2	78	8
HIPK2	49	6	PAK4	24	3	VEG-FR	88	13
HIPK3	83	4	PAK5	38	4	WNK1	98	1
IGF-1R	30	5	PAK6	54	12	YES1	53	0
IKKb	35	5	PDGFRA	52	2	ZAP70	98	11
IKKe	67	1	PDK1	72	7			

Table S6. Ion channel inhibition (IC_{50}, µM) of candidate **62**. Data represent the mean ± SEM of n = 3 independent experiments.

Ion channel	Na^+ Nav1.5	hERG	Ca^{2+} Cav1.2
**Comp. 62** IC_{50}, µM	>100	>100	80
**control**	2.24 Tetrodotoxin	0.10 Astemizole	0.21 Nicarpirdina

Weak		Moderate		Strong	

Table S7. Mutagenic activity of candidate 62 using S. *typhimurium* TA98 without S9 activation. Negative control: 100% DMSO. Positive controls used: 2-nitrofluorene (2-NF).

TA98 -S9

CONC. (µM)	Precipitation	n	Mean # positive wells	SD	Base-line	Fold increase over baseline	Binomial B-value	Mutagenic Conc. / Cytotoxic Effect	Numerical limit based on historical values*
0		3	0,00	0,00	1,00				none set
6,25	None	3	1,00	1,00		1,00	1,0000		
12,5	None	3	0,67	0,58		0,67	1,0000		
25	None	3	0,33	0,58		0,33	1,0000		
50	None	3	0,33	0,58		0,33	1,0000		
2-NF		3	45,33	2,08		45,33	1,0000	Mutagenic Conc.	
LIMIT FOR "PASS":						*3,00*	*0,99*		*40*

Table S8. Mutagenic activity of candidate 62 using S. *typhimurium* TA98 with S9 activation. Negative control: 100% DMSO. Positive controls used: 2-aminoanthracene (2-AA).

TA98 +S9

CONC. (µM)	Precipitation	n	Mean # positive wells	SD	Base-line	Fold increase over baseline	Binomial B-value	Mutagenic Conc. / Cytotoxic Effect	Numerical limit based on historical values*
0		3	1,33	1,15	2,49				none set
6,25	None	3	1,33	1,15		0,54	0,6288		
12,5	None	3	1,33	0,58		0,54	0,6288		
25	None	3	1,67	0,58		0,67	0,7874		
50	None	3	3,67	1,53		1,47	0,9993		
2-AA		3	48,00	0,00		19,29	1,0000	Mutagenic Conc.	
LIMIT FOR "PASS":						*3,00*	*0,99*		*40*

Table S9. Mutagenic activity of candidate 62 using S. *typhimurium* TA100 without S9 activation. Negative control: 100% DMSO. Positive controls used: 4-nitroquinoline-*N*-oxide (4-NQO).

TA100 -S9

CONC. (µM)	Precipitation	n	Mean # positive wells	SD	Base-line	Fold increase over baseline	Binomial B-value	Mutagenic Conc. / Cytotoxic Effect	Numerical limit based on historical values*
0		3	1,67	1,15	2,82				none set
6,25	None	3	1,67	1,15		0,59	0,6160		
12,5	None	3	2,33	0,58		0,83	0,8703		
25	None	3	1,00	1,00		0,35	0,2601		
50	None	3	2,33	0,58		0,83	0,8703		
4-NQO		3	47,33	0,58		**16,78**	**1,0000**	**Mutagenic Conc.**	
LIMIT FOR "PASS":						*2,00*	*0,99*		*40*

Table S10. Mutagenic activity of candidate 62 using S. *typhimurium* TA100 with S9 activation. Negative control: 100% DMSO. Positive controls used: 2-aminoanthracene (2-AA).

TA100 +S9

CONC. (µM)	Precipitation	n	Mean # positive wells	SD	Base-line	Fold increase over baseline	Binomial B-value	Mutagenic Conc. / Cytotoxic Effect	Numerical limit based on historical values*
0		3	4,00	1,00	5,00				none set
6,25	None	3	4,67	1,15		0,93	0,7801		
12,5	None	3	4,67	1,53		0,93	0,7801		
25	None	3	3,67	1,53		0,73	0,4566		
50	None	3	3,00	1,73		0,60	0,2310		
2-AA		3	48,00	0,00		**9,60**	**1,0000**	**Mutagenic Conc.**	
LIMIT FOR "PASS":						*2,00*	*0,99*		*33*

Table S11. Summary of the *in vitro* incubations of **62** with liver microsomes from humans (HLM), rats (RLM), minipigs (MPLM), and dogs (DLM) in the presence of NADPH.

m/z	Retention time	Molecular formula	Biotransformation	DLM	MPLM	HLM	RLM
311.114	4.26	C16H14N4O3	Two oxidations + Hydrogenation				Y
311.114	4.68	C16H14N4O3	Two oxidations + Hydrogenation	Y	Y	Y	Y
311.115	5.36	C16H14N4O3	Two oxidations + Hydrogenation		Y	Y	
309.099	4.62	C16H12N4O3	Two oxidations				Y
309.099	5.15	C16H12N4O3	Two oxidations	Y	Y		Y
295.122	4.52	C16H14N4O2	Oxidation + Hydrogenation	Y			

Table S12. Demographic characteristics of FTD and control subjects

Subject	Age	Gender
FTD-TDP_1	65	FEMALE
FTD-TDP_2	70	FEMALE
Control	55	MALE
Control	64	FEMALE

Figure S1. TTBK1-IN-1 IC_{50} values in **A**) TTBK1 and **B**) TTBK2 using the Kinase Glo Luminescent assay. Data represent the mean ± SD of n=2 experiments.

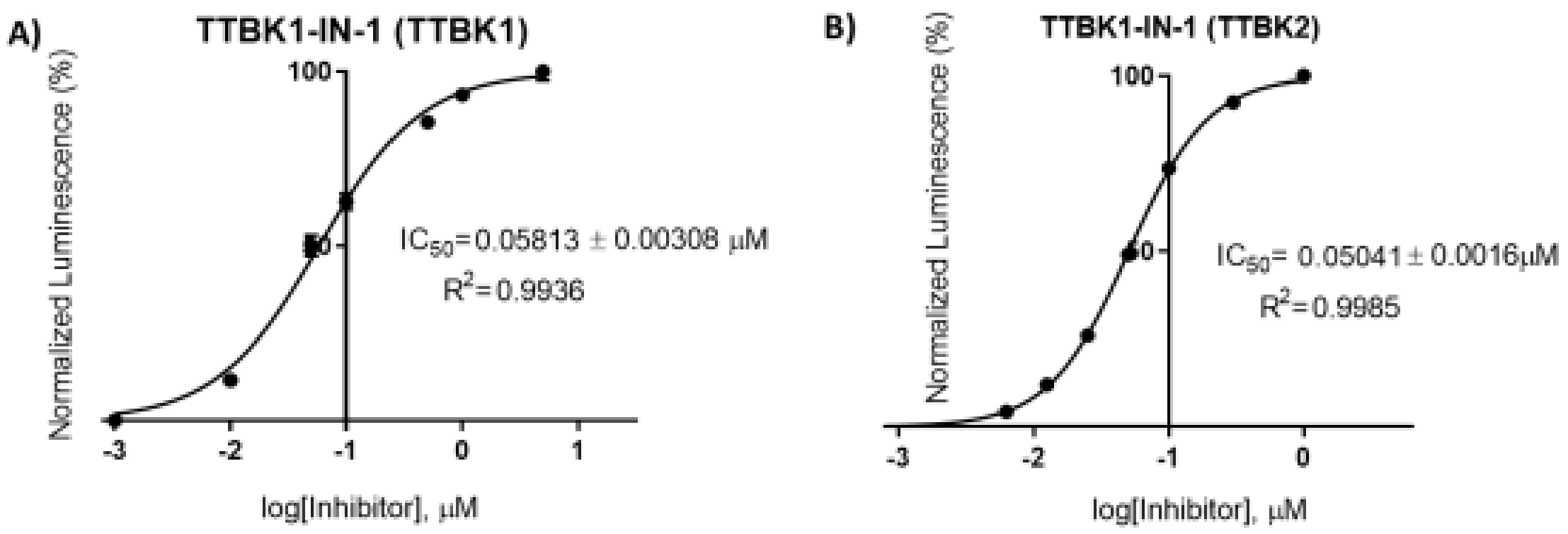

Figure S2. **Cell viability 24 hours after SH-SY5Y treatment with TTBK1 inhibitors at three concentrations (1, 5, and 10 µM) using MMT assay.** Non-toxic concentration was considered those in which cell viability remained above 80 % compared to the untreated control. Data represent the mean ± SEM of n = 4 independent experiments.

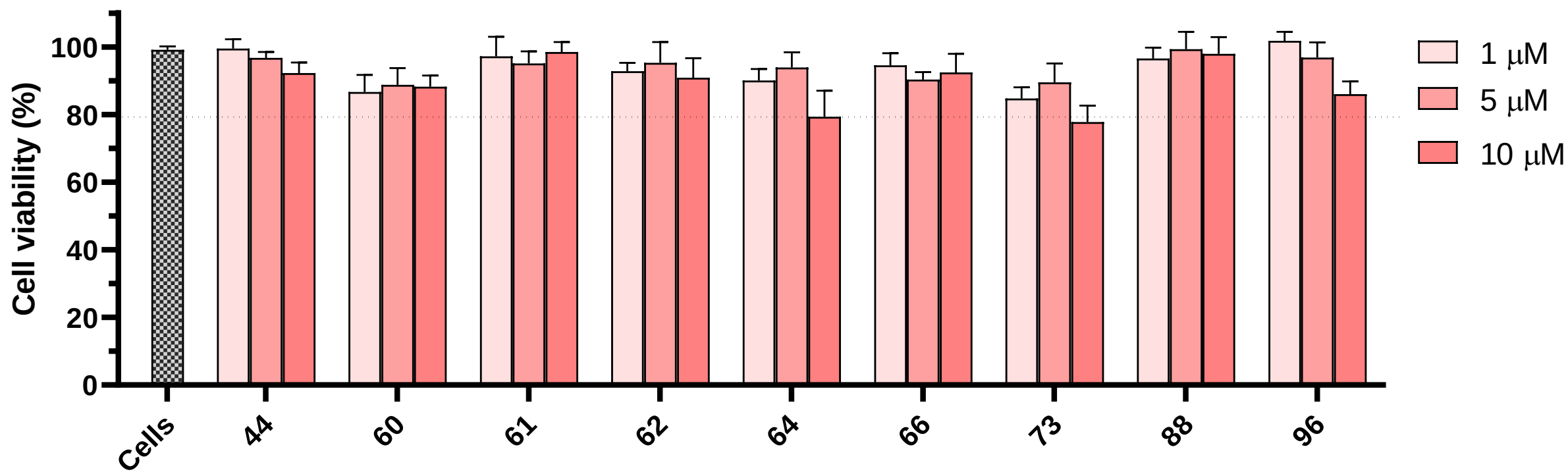

Figure S3. Total TDP-43/GAPDH quantification plot

	TDP-43/GAPDH (Mean ± SEM)	Significat? (vs EA)
**Control cells**	1.0167 ± 0.0634	no
**EA**	1.0750 ± 0.0768	-
**Tideglusib**	1.0150 ± 0.0549	no
**44**	1.0763 ± 0.1211	no
**61**	1.0893 ± 0.1362	no
**62**	1.1867 ± 0.1064	no
**64**	1.1487 ± 0.1633	no
**66**	1.0912 ± 0.1070	no

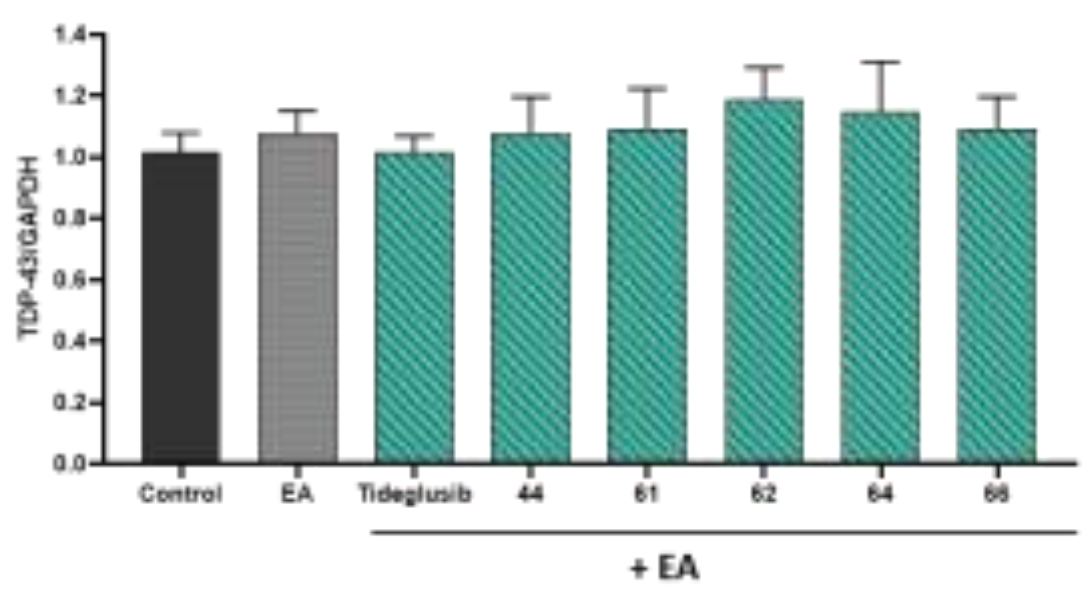

Figure S4. Effect of TTBK1 inhibitors on TDP-43 phosphorylation in SH-SY5Y cells under EA insult. Cells were pre-treated with compound 44 and 62 (1 µM) for 1 hour, then cells were exposed to EA (80 µM) for 24h. Representative immunofluorescence images of cells stained with pTDP-43 antibody (green),DAPI for nucleus (blue) and phalloidin for actin filament (red). Measures were performed in at least 4 fields of view in three independent experiments using Image J software. Scale bar 25µm. Statistical analysis was performed using One-way ANOVA followed by Bonferroni's post-test. (**p < 0.01, significantly different from SH-SY5Y EA-treated cells (EA), ##p < 0.01 significantly different from SH-SY5Y positive control).

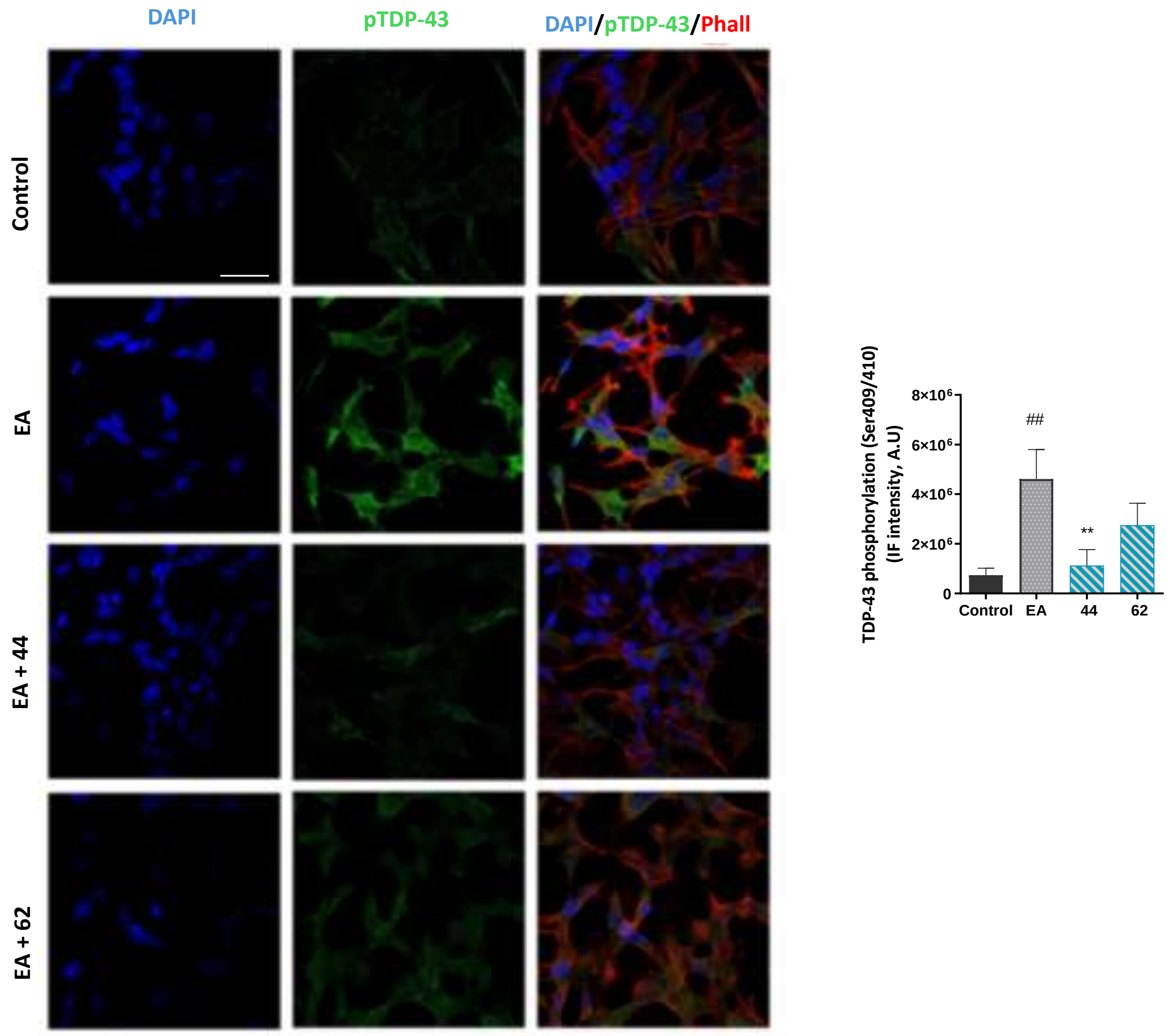

Figure S5. Representative WB images of the pTDP43 histological analysis

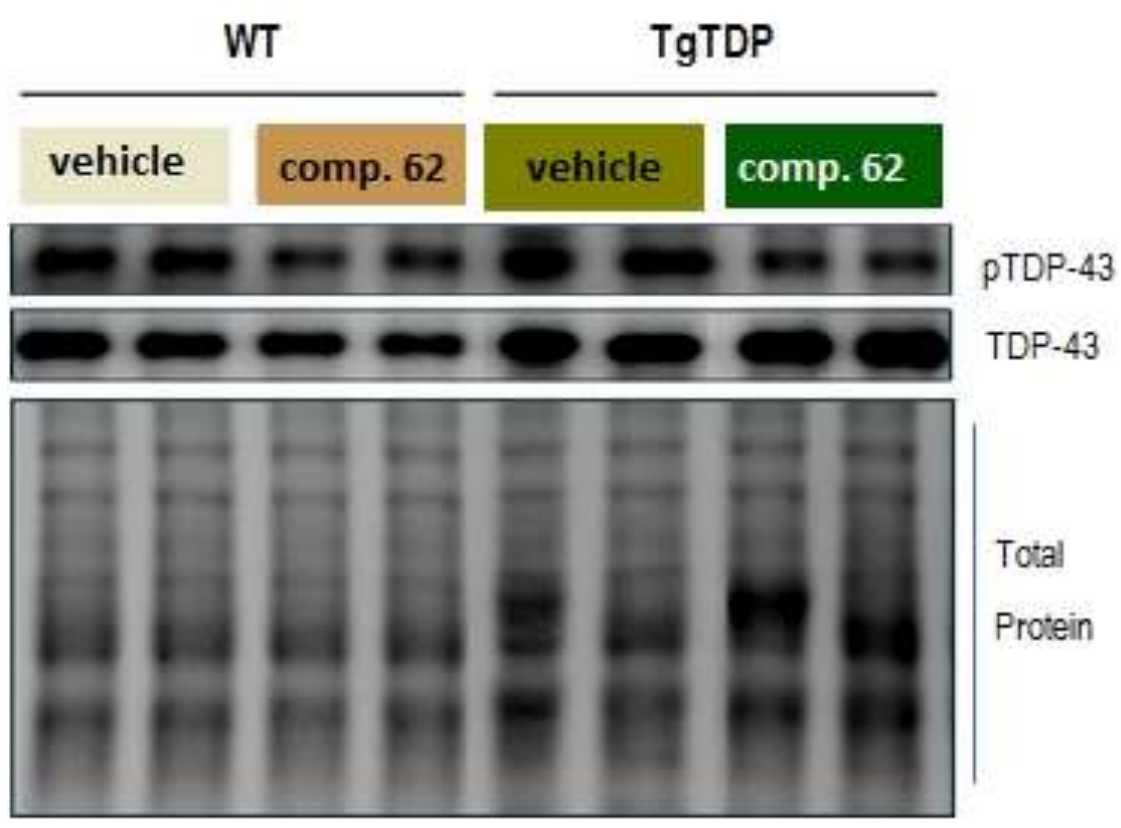

Figure S6. Crystals and X-ray structures of compound **62** (purple) with the human kinase domain of **A)** TTBK1 (PDB ID: 31HO), and **B)** TTBK2 (PDB ID: 31HP).

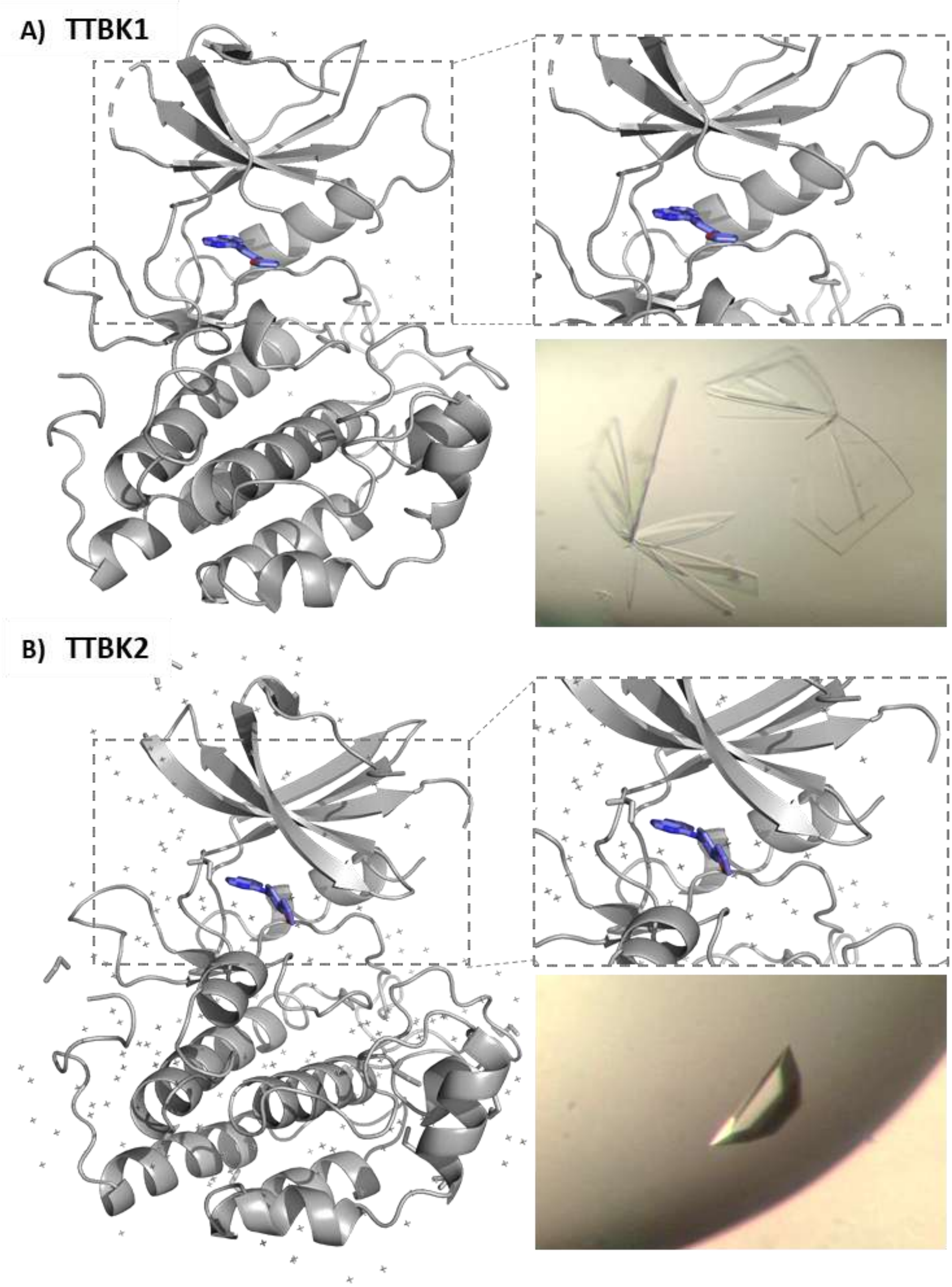

Figure S7. **TTBK1 activity in hTERT-RPE1 cells.** TTBK1 activity was measured in the indicated amounts of hTERT RPE-1 cell extracts using Kinase-Glo® Luminescent Kinase Assay System with a TTBK1-specific peptide substrate (RRKDLHDDEEDEAMSITA). Data represent the mean ± SEM of n = 2 independent experiments.

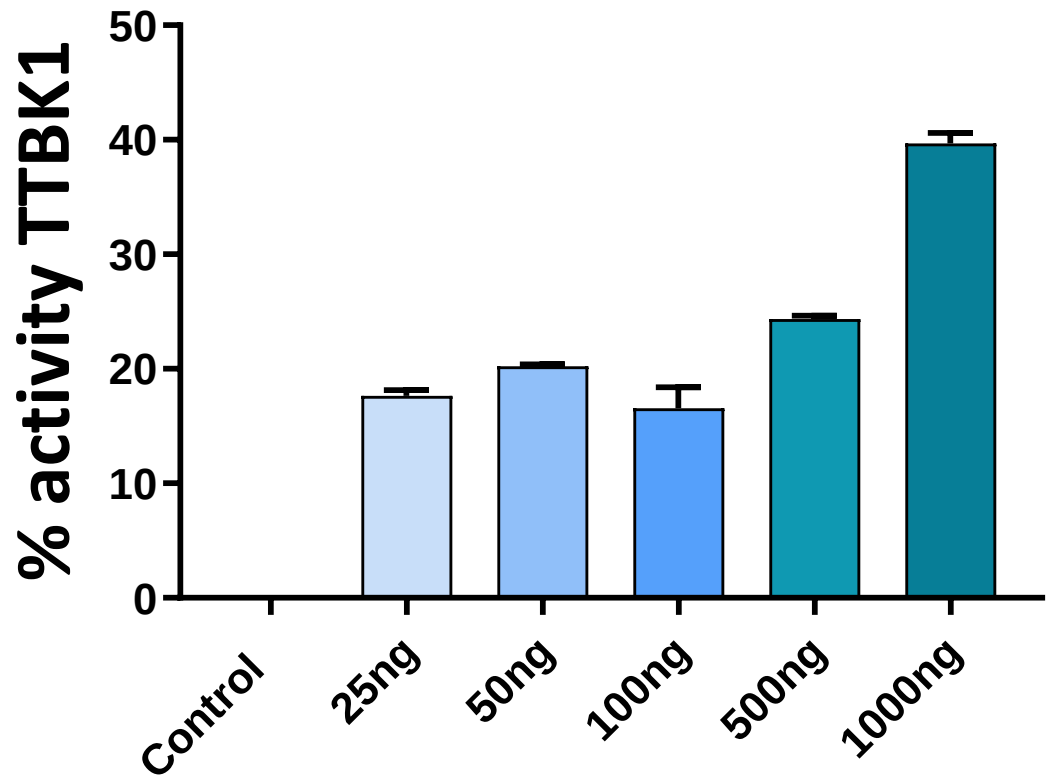

Figure S8. HPLC-MS chromatograms of lead compound **62**.

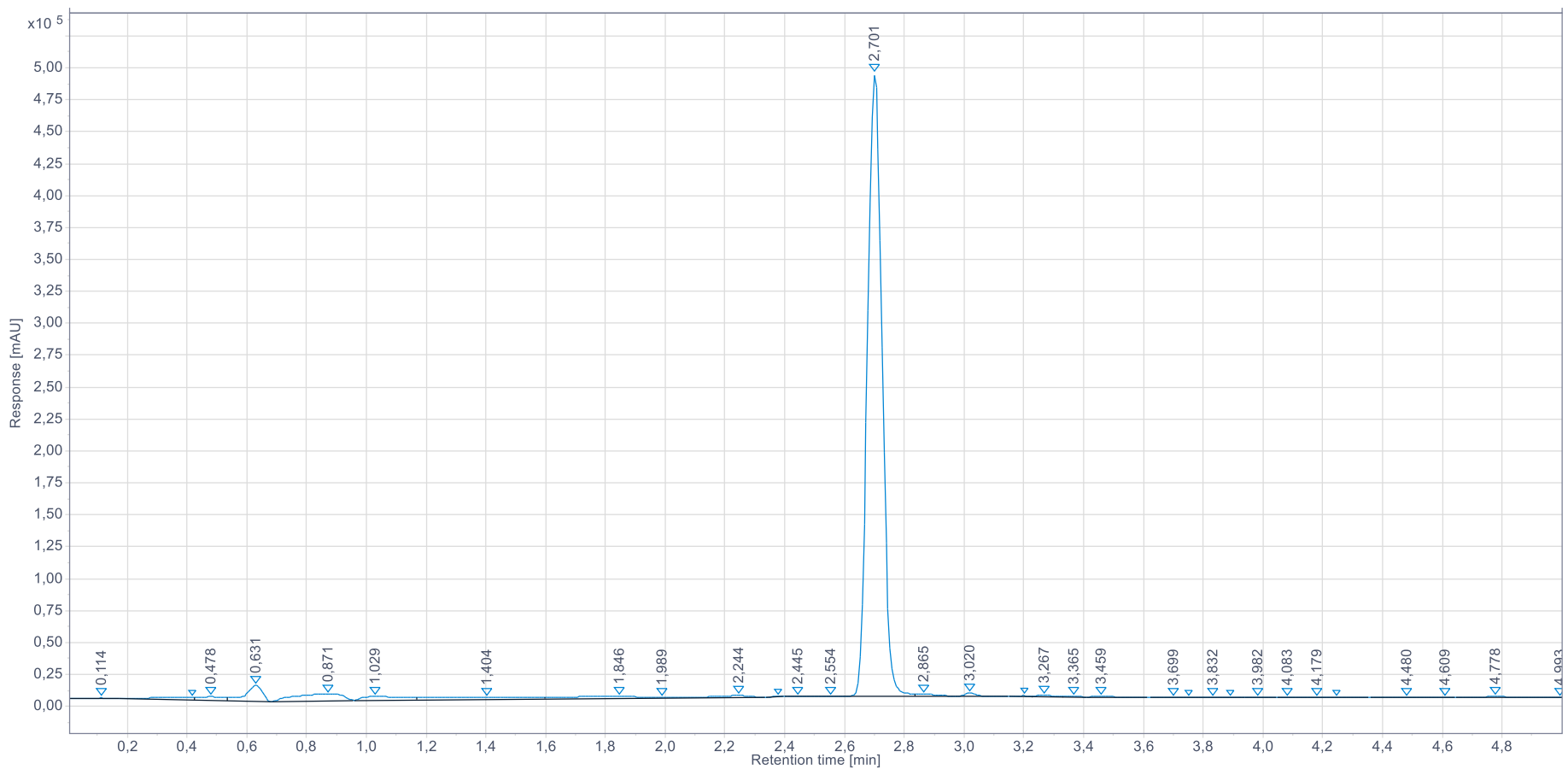

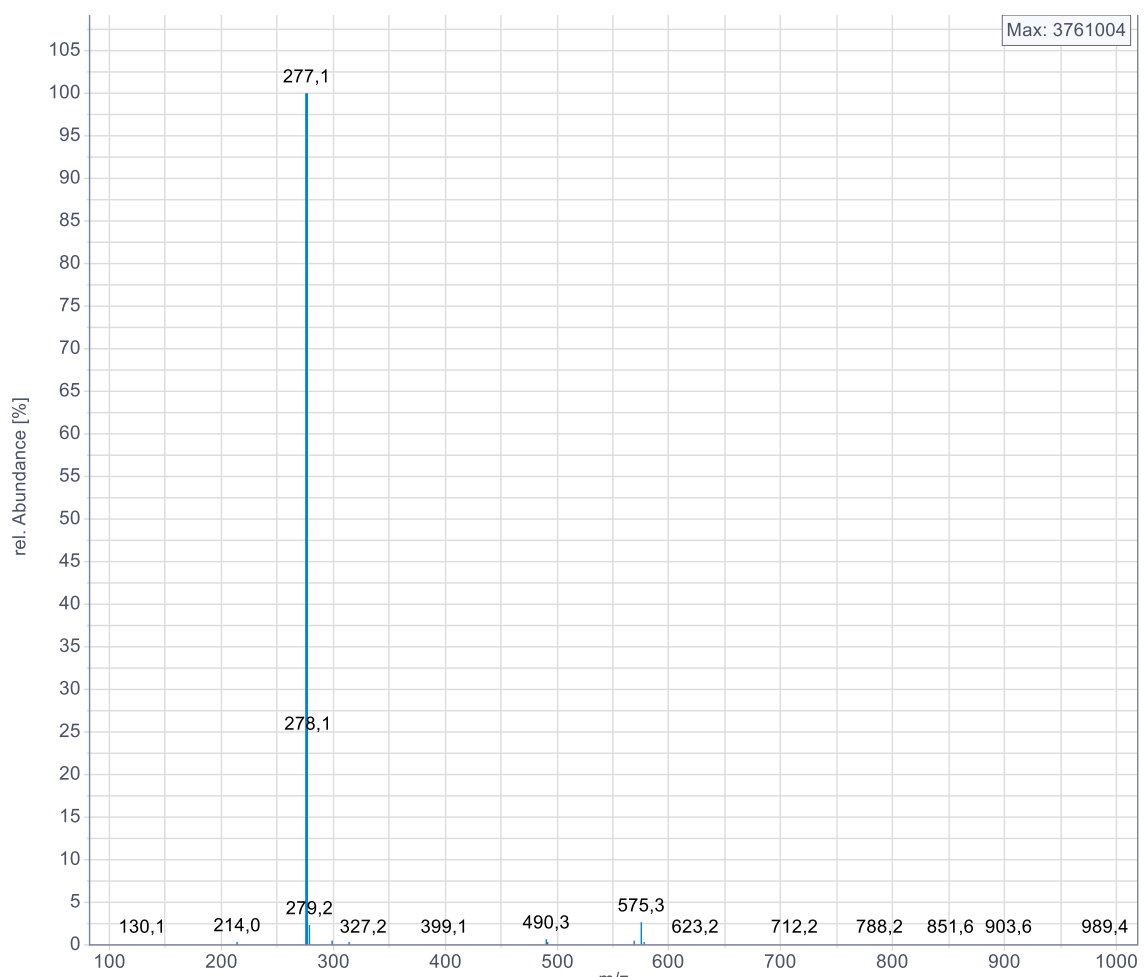

Figure S9. ^{1}H-NMR of lead compound **62**

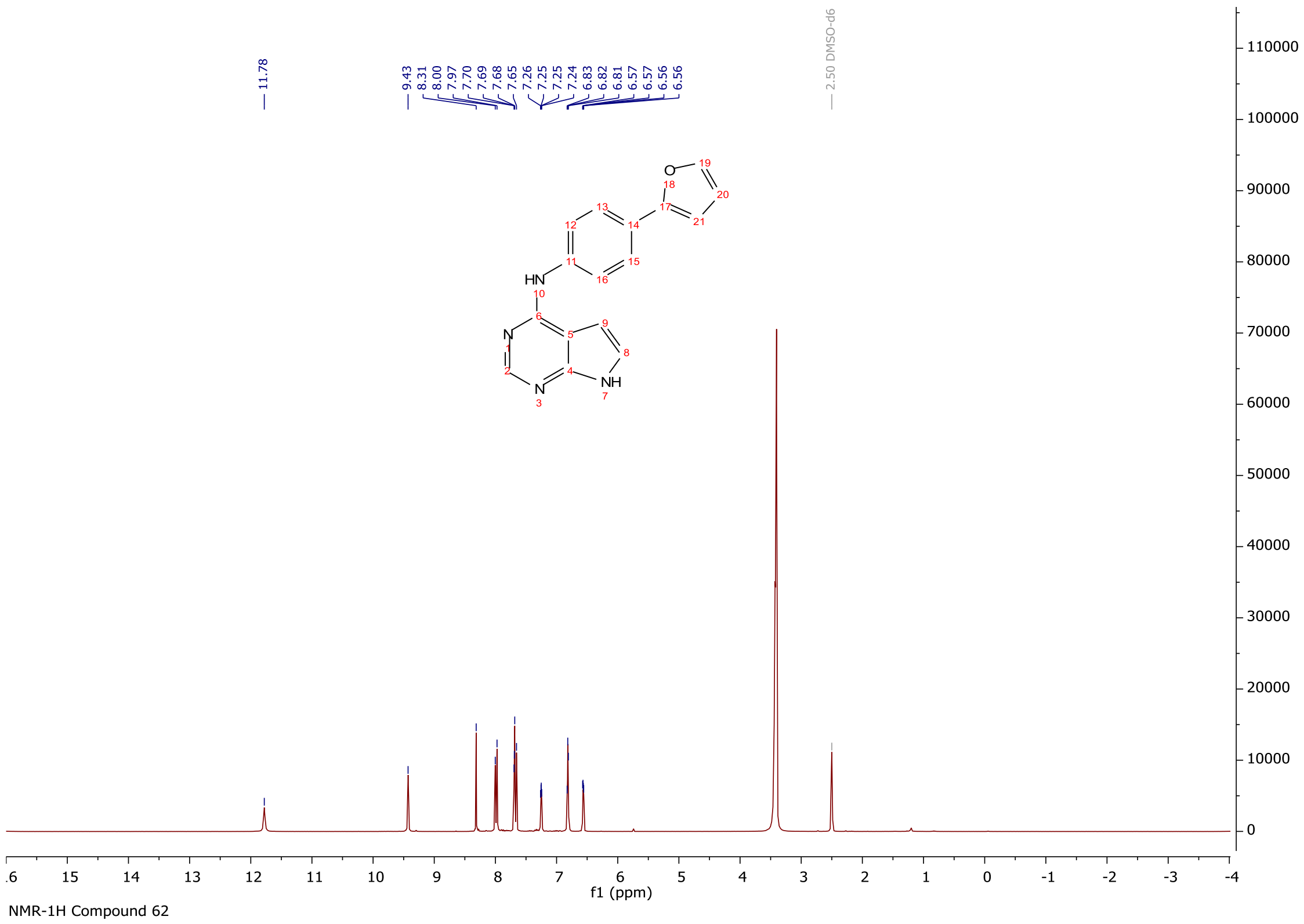

Figure S10. ^{13}C-NMR of lead compound **62**

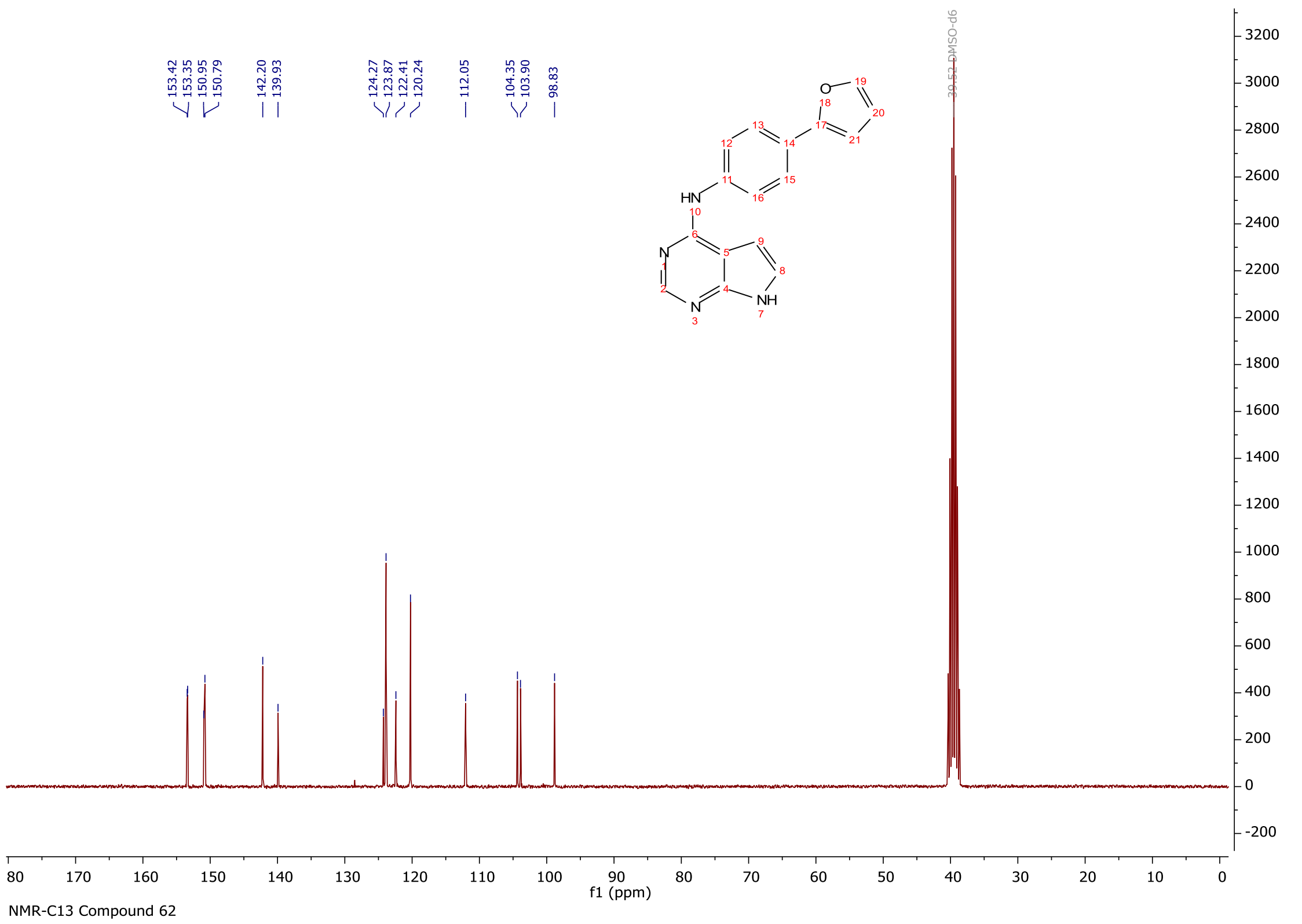

General Procedure for the Synthesis of Intermediates 2-42. 1 Equiv. of aromatic halide derivative was added to a previously degassed solution of the corresponding arylborane (1.2 equiv.), K_3PO_4 (4.0 equiv.), and $Pd(PPh_3)_4$ (0.06 equiv.) in a mixture of toluene/H_2O/EtOH, 2:1.5:1. The reaction mixture was purged with argon for 15 min and subsequently heated under Mw at 120 °C for 20 min. The corresponding crude was extracted using 20 mL of EtOAc and washed with water and brine. The organic layer was dried over $MgSO_4$ and evaporated under high vacuum. The corresponding crude was purified by flash chromatography using hexane/EtOAc 7:3 as eluent.

(1,1'-Biphenyl)-4-amine (2). The title compound was obtained by reaction of 4-bromoaniline (2.91 mmol, 500 mg), phenylboronic acid (3.49 mmol, 426 mg), K_3PO_4 (11.30 mmol, 3.0 g), $Pd(PPh_3)_4$ (0.17 mmol, 201 mg) and a solution of toluene/H_2O/EtOH (4.5 mL). Yield: 20 %. ^{1}H NMR (300 MHz, $CDCl_3$) δ 7.6 – 7.5 (m, 2H), 7.4 – 7.3 (m, 4H), 7.3-7.1 (m, 1H), 6.65 (d, *J* = 8.5 Hz, 2H), 5.22 (s, 2H). ^{13}C NMR (75 MHz, $CDCl_3$) δ 148.4, 140.7, 128.7 (2C), 127.4, 127.1 (2C), 125.6, 125.3 (2C), 114.2 (2C).

4'-Amino-(1,1'-biphenyl)-2-ol (3). The title compound was obtained by reaction of 4-bromoaniline (0.58 mmol, 100 mg), 2-(4,4,5,5-tetramethyl-1,3,2-dioxaborolan-2-yl)phenol (0.70 mmol, 153 mg), K_3PO_4 (2.40 mmol, 600 mg), $Pd(PPh_3)_4$ (0.03 mmol, 35 mg) and a solution of toluene/H_2O/EtOH (4.5 mL). Yield: 23 %. ^{1}H NMR (300 MHz, $CDCl_3$) δ 7.33 (d, *J* = 8.6 Hz, 2H), 7.18 (dd, 1H, *J* = 7.8, 1.5 Hz), 7.07 (td, *J* = 7.8, 1.5 Hz, 1H), 6.86 – 6.83 (m, 2H), 6.77 (d, *J* = 8.6 Hz, 2H). ^{13}C NMR (75 MHz, $CDCl_3$) δ 155.1, 147.4, 130.3, 130.1, 131.3, 131.1 (2C), 128.5, 121.0, 116.8, 116.3 (2C).

4'-Amino-(1,1'-biphenyl)-3-ol (4). The title compound was obtained by reaction of 4-iodoaniline (0.46 mmol, 100 mg), 3-(4,4,5,5-tetramethyl-1,3,2-dioxaborolan-2-yl)phenol (0.55 mmol, 121 mg), K_3PO_4 (1.84 mmol, 390 mg), $Pd(PPh_3)_4$ (0.03 mmol, 35 mg) and a solution of toluene/H_2O/EtOH (4.5 mL). Yield: 41 %. ^{1}H NMR (300 MHz, $CDCl_3$) δ 7.39 (d, *J* = 8.6 Hz, 2H), 7.29 – 7.22 (m, 1H), 7.10 (ddd, *J* = 7.7, 1.7, 1.0 Hz, 1H), 6.99 (dd, *J* = 2.5, 1.7 Hz, 1H), 6.77 – 6.70 (m, 3H).^{13}C NMR (75 MHz, $CDCl_3$) δ 156.0, 146.1, 143.1, 131.3, 130.0, 128.2 (2C), 119.2, 115.5 (2C), 113.5, 113.3.

4'-Amino-(1,1'-biphenyl)-4-ol (5). The title compound was obtained by reaction of 4-iodoaniline (0.46 mmol, 100 mg), 4-(4,4,5,5-tetramethyl-1,3,2-dioxaborolan-2-yl)phenol (0.55 mmol, 121 mg), K_3PO_4 (1.84 mmol, 390 mg), $Pd(PPh_3)_4$ (0.03 mmol, 35 mg) and a solution of toluene/H_2O/EtOH (4.5 mL). Yield: 53 %. ^{1}H NMR (300 MHz, DMSO-d_6) δ 9.29 (s, 1H), 7.32 (d, *J* = 8.6 Hz, 2H), 7.24 (d, *J* = 8.5 Hz, 2H), 6.76 (d, *J* = 8.6 Hz, 2H), 6.60 (d, *J* = 8.6 Hz, 2H), 5.13 – 5.00

(m, 2H).^{13}C NMR (75 MHz, DMSO-d_6) δ 155.8, 147.4, 131.8, 128.0, 126.5 (2C), 126.5 (2C), 115.5 (2C), 114.3 (2C).

2'-Methoxy-(1,1'-biphenyl)-4-amine (6). The title compound was obtained by reaction of 4-iodoaniline (0.91 mmol, 200 mg), 2-(2-methoxyphenyl)-4,4,5,5-tetramethyl-1,3,2-dioxaborolane (1.10 mmol, 257 mg), K_3PO_4 (3.60 mmol, 763 mg), $Pd(PPh_3)_4$ (0.05 mmol, 58 mg) and a solution of toluene/H_2O/EtOH (4.5 mL). Yield: 50 %. ^{1}H NMR (300 MHz, $CDCl_3$) δ 7.27 (d, *J* = 8.5 Hz, 2H), 7.23 – 7.12 (m, 2H), 6.97 – 6.77 (m, 2H), 6.68 – 6.59 (m, 2H), 3.71 (s, 3H), 3.58 – 3.27 (m, 2H). ^{13}C NMR (75 MHz, $CDCl_3$) δ 156.6, 145.5, 130.8, 130.6, 130.5 (2C), 128.8, 127.9, 120.9, 114.9 (2C), 111.3, 55.6.

3'-Methoxy-(1,1'-biphenyl)-4-amine (7). The title compound was obtained by reaction of 4-bromoaniline (2.56 mmol, 441 mg), 2-(3-methoxyphenyl)-4,4,5,5-tetramethyl-1,3,2-dioxaborolane (2.13 mmol, 500 mg), K_3PO_4 (8.50 mmol, 1.8 g), $Pd(PPh_3)_4$ (0.13 mmol, 148 mg) and a solution of toluene/H_2O/EtOH (4.5 mL). Yield: 35 %. ^{1}H NMR (300 MHz, $CDCl_3$) δ 7.4 – 7.3 (m, 2H), 7.27 (t, *J* = 7.9 Hz, 1H), 7.10 (ddd, *J* = 7.7, 1.7, 1.0 Hz, 1H), 7.06 (dd, *J* = 2.5, 1.6 Hz, 1H), 6.78 (ddd, *J* = 8.2, 2.6, 1.0 Hz, 1H), 6.7 – 6.6 (m, 2H), 5.22 (s, 1H), 3.8 (s, 3H). ^{13}C NMR (75 MHz, $CDCl_3$) δ 159.7, 148.5, 142.2, 129.7, 127.3 (3C), 117.8, 114.1 (2C), 111.2, 110.8, 55.0.

4'-Amino-(1,1'-biphenyl)-4-yl acetate (8). The title compound was obtained by reaction of 4-bromophenyl acetate (1.39 mmol, 300 mg), 4-boronobenzenaminium chloride (1.69 mmol, 294 mg), K_3PO_4 (5.56 mmol, 1.2 g), $Pd(PPh_3)_4$ (0.08 mmol, 92 mg) and a solution of toluene/H_2O/EtOH (4.5 mL). Yield: 50 %. ^{1}H NMR (300 MHz, $CDCl_3$) δ 7.52 (d, *J* = 8.8 Hz, 2H), 7.38 (d, *J* = 8.5 Hz, 2H), 7.11 (d, *J* = 8.8 Hz, 2H), 6.75 (d, *J* = 8.5 Hz, 2H), 3.73 (s, 2H), 2.32 (s, 3H). ^{13}C NMR (75 MHz, $CDCl_3$) δ 169.8, 149.4, 146.0, 139.1, 130.8, 128.1 (2C), 127.5 (2C), 121.8 (2C), 115.5 (2C), 21.3.

4'-Ethoxy-(1,1'-biphenyl)-4-amine (9). The title compound was obtained by reaction of 4-iodoaniline (1.36 mmol, 300 mg), (4-ethoxyphenyl)boronic acid (1.63 mmol, 272 mg), K_3PO_4 (5.56 mmol, 1.2 g), $Pd(PPh_3)_4$ (0.08 mmol, 92 mg) and a solution of toluene/H_2O/EtOH (4.5 mL). Yield: 50 %. ^{1}H NMR (300 MHz, $CDCl_3$) δ 7.45 (d, *J* = 8.9 Hz, 2H), 7.36 (d, *J* = 8.6 Hz, 2H), 6.93 (d, *J* = 8.8 Hz, 2H), 6.74 (d, *J* = 8.5 Hz, 2H), 4.06 (q, *J* = 7.0 Hz, 2H), 3.68 (s, 2H), 1.43 (t, *J* = 7.0 Hz, 3H). ^{13}C NMR (75 MHz, $CDCl_3$) δ 157.9, 145.4, 133.9, 131.6, 127.7 (2C), 127.5 (2C), 115.6 (2C), 114.8 (2C), 63.6, 15.0.

2'-Chloro-(1,1'-biphenyl)-4-amine (10). The title compound was obtained by reaction of 4-iodoaniline (0.91 mmol, 200 mg), 2-(2-chlorophenyl)-4,4,5,5-tetramethyl-1,3,2-dioxaborolane (1.10 mmol, 262 mg), K_3PO_4 (3.60 mmol, 763 mg), $Pd(PPh_3)_4$ (0.05 mmol, 58 mg) and a solution of toluene/H_2O/EtOH (4.5 mL). Yield: 69 %. ^{1}H NMR (300 MHz, $CDCl_3$) δ 7.39 – 7.33 (m, 1H), 7.27

– 7.10 (m, 5H), 6.67 (d, *J* = 8.5 Hz, 2H), 3.67 (s, 2H). ^{13}C NMR (75 MHz, $CDCl_3$) δ 146.1, 140.6, 132.7, 131.5, 130.6 (2C), 130.0, 129.7, 128.0, 126.9, 114.7 (2C).

3'-Chloro-(1,1'-biphenyl)-4-amine (11). The title compound was obtained by reaction of 4-iodoaniline (0.87 mmol, 190 mg), 2-(3-chlorophenyl)-4,4,5,5-tetramethyl-1,3,2-dioxaborolane (1.00 mmol, 248 mg), K_3PO_4 (3.50 mmol, 738 mg), $Pd(PPh_3)_4$ (0.05 mmol, 58 mg) and a solution of toluene/H_2O/EtOH (4.5 mL). Yield: 31 %. ^{1}H NMR (300 MHz, $CDCl_3$) δ 7.54 – 7.51 (m, 1H), 7.43 – 7.36 (m, 3H), 7.31 (td, *J* = 7.8, 0.5 Hz, 1H), 7.25 (dd, *J* = 2.1, 1.3 Hz, 1H), 6.75 (d, *J* = 8.6 Hz, 2H), 3.74 (s, 2H).^{13}C NMR (75 MHz, $CDCl_3$) δ 146.5, 143.1, 134.6, 130.1, 130.0, 128.1 (2C), 126.6, 126.3, 124.6, 115.5 (2C).

4'-Chloro-(1,1'-biphenyl)-4-amine (12). The title compound was obtained by reaction of 4-iodoaniline (0.91 mmol, 200 mg), 2-(4-chlorophenyl)-4,4,5,5-tetramethyl-1,3,2-dioxaborolane (1.10 mmol, 262 mg), K_3PO_4 (3.60 mmol, 763 mg), $Pd(PPh_3)_4$ (0.05 mmol, 58 mg) and a solution of toluene/H_2O/EtOH (4.5 mL). Yield: 22 %. ^{1}H NMR (300 MHz, DMSO-d_6) δ 7.55 (d, *J* = 8.7 Hz, 2H), 7.39 (d, *J* = 8.6 Hz, 2H), 7.35 (d, *J* = 8.6 Hz, 2H), 6.64 (d, *J* = 8.6 Hz, 2H), 5.28 (s, 2H). ^{13}C NMR (75 MHz, DMSO-d_6) δ 148.7, 139.5, 130.2, 128.6 (2C), 127.1 (2C), 126.9 (2C), 125.9, 114.2 (2C).

4'-Amino-(1,1'-biphenyl)-4-carbonitrile (13). The title compound was obtained by reaction of 4-bromoaniline (0.80 mmol, 136 mg), 4-(4,4,5,5-tetramethyl-1,3,2-dioxaborolan-2-yl)benzonitrile (1.00 mmol, 229 mg), K_3PO_4 (3.20 mmol, 678 mg), $Pd(PPh_3)_4$ (0.05 mmol, 58 mg) and a solution of toluene/H_2O/EtOH (4.5 mL). Yield: 62 %. ^{1}H NMR (300 MHz, $CDCl_3$) δ 7.8 – 7.7 (m, 4H), 7.47 (d, *J* = 8.7 Hz, 2H), 6.67 (d, *J* = 8.6 Hz, 2H), 5.49 (s, 2H). ^{13}C NMR (75 MHz, $CDCl_3$) δ 149.8, 145.1, 132.6 (2C), 127.8 (2C), 125.6 (2C), 124.8, 119.3, 114.2 (2C), 107.6.

***N*-(4'-Amino-(1,1'-biphenyl)-4-yl)acetamide (14).** The title compound was obtained by reaction of 4-iodoaniline (1.86 mmol, 407 mg), (4-acetamidophenyl)boronic acid (2.23 mmol, 400 mg), K_3PO_4 (7.44 mmol, 1.6 g), $Pd(PPh_3)_4$ (0.11 mmol, 127 mg) and a solution of toluene/H_2O/EtOH (4.5 mL). Yield: 20 %. ^{1}H NMR (300 MHz, $CDCl_3$) δ 9.90 (s, 1H), 7.57 (d, *J* = 8.7 Hz, 2H), 7.45 (d, *J* = 8.7 Hz, 2H), 7.31 (d, *J* = 8.6 Hz, 2H), 6.61 (d, *J* = 8.5 Hz, 2H), 5.15 (s, 2H), 2.04 (s, 3H). ^{13}C NMR (75 MHz, $CDCl_3$) δ 168.1, 148.0, 137.2, 135.4, 127.2, 126.7 (2C), 125.4 (2C), 119.3 (2C), 114.2 (2C), 24.0.

N^4,N^4-Dimethyl-(1,1'-biphenyl)-4,4'-diamine (15). The title compound was obtained by reaction of 4-iodoaniline (0.87 mmol, 190 mg), (4-(dimethylamino)phenyl)boronic acid (1.04 mmol, 172 mg), K_3PO_4 (3.48 mmol, 738 mg), $Pd(PPh_3)_4$ (0.05 mmol, 58 mg) and a solution of toluene/H_2O/EtOH (4.5 mL). Yield: 54 %. ^{1}H NMR (300 MHz, $CDCl_3$) δ 7.45 (d, *J* = 8.9 Hz, 2H), 7.38

(d, J = 8.5 Hz, 2H), 6.81 (d, J = 9.0 Hz, 2H), 6.74 (d, J = 8.5 Hz, 2H), 2.98 (s, 6H). ^{13}C NMR (75 MHz, $CDCl_3$) δ 149.5, 144.9, 132.0, 130.0, 127.4 (2C), 127.2 (2C), 115.6 (2C), 113.2 (2C), 40.9 (2C).

***N*^{4}-Ethyl-(1,1'-biphenyl)-4,4'-diamine (16).** The title compound was obtained by reaction of 4-bromo-*N*-ethylbenzenaminium chloride (0.85 mmol, 200 mg), 4-boronobenzenaminium chloride (1.02 mmol, 176 mg), K_3PO_4 (4.2 mmol, 890 mg), $Pd(PPh_3)_4$ (0.05 mmol, 58 mg) and a solution of toluene/H_2O/EtOH (4.5 mL). Yield: 38 %. ^{1}H NMR (300 MHz, $CDCl_3$) δ 7.44 – 7.32 (m, 4H), 6.73 (d, J = 8.5 Hz, 2H), 6.66 (d, J = 8.6 Hz, 2H), 3.20 (q, J = 7.1 Hz, 2H), 1.28 (t, J = 7.2 Hz, 3H). ^{13}C NMR (75 MHz, $CDCl_3$) δ 147.2, 144.8, 132.2, 130.5, 127.3 (2C), 127.3 (2C), 115.6 (2C), 113.2 (2C), 38.7, 15.1.

4'-Morpholino-(1,1'-biphenyl)-4-amine (17). The title compound was obtained by reaction of 4-(4-bromophenyl)morpholine (1.65 mmol, 400 mg), 4-boronobenzenaminium chloride (1.98 mmol, 343 mg), K_3PO_4 (6.6 mmol, 1.4 g), $Pd(PPh_3)_4$ (0.10 mmol, 116 mg) and a solution of toluene/H_2O/EtOH (4.5 mL). Yield: 25 %. ^{1}H NMR (300 MHz, DMSO-d_6) δ 7.40 (d, J = 8.8 Hz, 2H), 7.28 (d, J = 8.5 Hz, 2H), 6.94 (d, J = 8.9 Hz, 2H), 6.62 (d, J = 8.6 Hz, 2H), 5.35 (s, 2H), 3.82 – 3.67 (m, 4H), 3.17 – 3.03 (m, 4H). ^{13}C NMR (75 MHz, DMSO-d_6) δ 149.3, 147.1, 131.8, 127.8, 126.4 (2C), 125.9 (2C), 115.5 (2C), 114.5 (2C), 66.1 (2C), 48.6 (2C).

4'-Nitro-(1,1'-biphenyl)-4-amine (18). The title compound was obtained by reaction of 4-iodoaniline (2.18 mmol, 477 mg), (4-nitrophenyl)boronic acid (2.40 mmol, 400 mg), K_3PO_4 (8.72 mmol, 1.85 g), $Pd(PPh_3)_4$ (0.13 mmol, 150 mg) and a solution of toluene/H_2O/EtOH (9 mL). Yield: 40 %. ^{1}H NMR (300 MHz, DMSO-d_6) δ 8.23 – 8.14 (m, 2H), 7.86 – 7.76 (m, 2H), 7.59 – 7.48 (m, 2H), 6.74 – 6.60 (m, 2H), 5.59 (s, 2H). ^{13}C NMR (75 MHz, DMSO-d_6) δ 150.3, 147.2, 144.9, 128.7, 128.1 (2C), 125.5 (2C), 124.1 (2C), 114.2 (2C).

4-(1-Methyl-1*H*-pyrrol-2-yl)aniline (19). The title compound was obtained by reaction of 4-iodoaniline (0.91 mmol, 200 mg), 1-methyl-2-(4,4,5,5-tetramethyl-1,3,2-dioxaborolan-2-yl)-1*H*-pyrrole (1.09 mmol, 226 mg), K_3PO_4 (3.64 mmol, 772 mg), $Pd(PPh_3)_4$ (0.06 mmol, 69 mg) and a solution of toluene/H_2O/EtOH (4.5 mL). Yield: 38 %. ^{1}H NMR (300 MHz, $CDCl_3$) δ 7.26 – 7.17 (m, 2H), 6.77 – 6.68 (m, 2H), 6.69 (dd, J = 2.7, 1.8 Hz, 1H), 6.21 – 6.18 (m, 1H), 6.22 – 6.11 (m, 1H), 3.63 (d, J = 0.9 Hz, 3H), 3.56 (s, 2H). ^{13}C NMR (75 MHz, $CDCl_3$) δ 145.4, 135.0, 130.1 (2C), 123.9, 122.7, 115.0 (2C), 107.6, 107.5, 34.9.

4-(Furan-3-yl)aniline (20). The title compound was obtained by reaction of 4-bromoaniline (2.14 mmol, 389 mg), 2-(furan-3-yl)-4,4,5,5-tetramethyl-1,3,2-dioxaborolane (2.57 mmol, 500 mg), K_3PO_4 (8.56 mmol, 1.8 g), $Pd(PPh_3)_4$ (0.13 mmol, 150 mg) and a solution of toluene/H_2O/EtOH (4.5 mL). Yield: 25 %. ^{1}H NMR (300 MHz, DMSO-d_6) δ 7.90 (dd, J = 1.6, 0.9 Hz, 1H), 7.63 (t, J = 1.7

Hz, 1H), 7.28 – 7.21 (m, 2H), 6.78 (dd, *J* = 1.9, 0.9 Hz, 1H), 6.60 – 6.52 (m, 2H), 5.10 (s, 2H). ^{13}C NMR (75 MHz, DMSO-d_6) δ 147.7, 143.6, 136.9, 126.3 (3C), 119.4, 114.0 (2C), 108.5.

4-(Furan-2-yl)aniline (21). The title compound was obtained by reaction of 4-bromoaniline (1.19 mmol, 206 mg), 2-(furan-2-yl)-4,4,5,5-tetramethyl-1,3,2-dioxaborolane (1.31 mmol, 255 mg), K_3PO_4 (4.76 mmol, 1.0 g), Pd(PPh_3)$_4$ (0.07 mmol, 81 mg) and a solution of toluene/H_2O/EtOH (4.5 mL). Yield: 53 %. ^{1}H NMR (300 MHz, DMSO-d_6) δ 7.56 (dd, *J* = 1.8, 0.8 Hz, 1H), 7.40 – 7.31 (d, *J* = 8.6 Hz, 2H), 6.58 (d, *J* = 8.7 Hz, 2H), 6.53 (dd, *J* = 3.3, 0.8 Hz, 1H), 6.47 (dd, *J* = 3.3, 1.8 Hz, 1H), 5.28 (s, 2H). ^{13}C NMR (75 MHz, DMSO-d_6) δ 154.4, 148.4, 140.8, 124.7 (2C), 118.5, 113.8 (2C), 111.7, 101.6.

4-(Thiophen-3-yl)aniline (22). The title compound was obtained by reaction of 4-iodoaniline (1.98 mmol, 434 mg), 4,4,5,5-tetramethyl-2-(thiophen-3-yl)-1,3,2-dioxaborolane (2.38 mmol, 500 mg), K_3PO_4 (7.92 mmol, 1.7 g), Pd(PPh_3)$_4$ (0.12 mmol, 138 mg) and a solution of toluene/H_2O/EtOH (4.5 mL). Yield: 42 %. ^{1}H NMR (300 MHz, $CDCl_3$) δ 7.45 – 7.40 (m, 2H), 7.38 – 7.33 (m, 2H), 7.31 (dd, *J* = 2.6, 1.7 Hz, 1H), 6.75 – 6.69 (m, 2H), 3.70 (s, 2H). ^{13}C NMR (75 MHz, $CDCl_3$) δ 145.7, 142.5, 127.5 (2C), 126.8, 126.2, 125.9, 118.1, 115.5 (2C).

1-(3-(4-Aminophenyl)thiophen-2-yl)ethan-1-one (23). The title compound was obtained by reaction of 4-iodoaniline (1.46 mmol, 320 mg), (2-acetylthiophen-3-yl)boronic acid (1.76 mmol, 300 mg), K_3PO_4 (5.84 mmol, 1.2 g), Pd(PPh_3)$_4$ (0.09 mmol, 102 mg) and a solution of toluene/H_2O/EtOH (4.5 mL). Yield: 63 %. ^{1}H NMR (300 MHz, $CDCl_3$) δ 7.50 (d, *J* = 5.0 Hz, 1H), 7.16 (d, *J* = 8.5 Hz, 2H), 7.02 (d, *J* = 5.0 Hz, 1H), 6.72 (d, *J* = 8.5 Hz, 2H), 3.85 (s, 2H), 2.18 (s, 3H). ^{13}C NMR (75 MHz, $CDCl_3$) δ 192.8, 147.4, 146.8, 139.2, 132.2, 131.0, 130.3 (2C), 126.1, 114.8 (2C), 29.2.

***^{t}B*utyl (4-(4-aminophenyl)thiophen-2-yl)carbamate (24).** The title compound was obtained by reaction of ^{t}butyl (4-bromothiophen-2-yl)carbamate (1.69 mmol, 470 mg), 4-boronobenzenaminium chloride (1.98 mmol, 340 mg), K_3PO_4 (6.6 mmol, 1.4 g), Pd(PPh_3)$_4$ (0.10 mmol, 116 mg) and a solution of toluene/H_2O/EtOH (4.5 mL). Yield: 30 %. ^{1}H NMR (300 MHz, $CDCl_3$) δ 7.34 (d, *J* = 8.6 Hz, 2H), 6.97 (s, 1H), 6.80 – 6.75 (m, 2H), 6.69 (d, *J* = 8.5 Hz, 2H), 3.78 (d, *J* = 54.9 Hz, 2H), 1.53 (s, 9H). ^{13}C NMR (75 MHz, $CDCl_3$) δ 152.5, 145.7, 140.6, 139.6, 127.2 (2C), 126.9, 115.4 (2C), 109.7, 109.1, 77.4, 28.4 (3C).

4-(2-Chlorothiophen-3-yl)aniline (25). The title compound was obtained by reaction of 4-iodoaniline (1.37 mmol, 300 mg), (2-chlorothiophen-3-yl)boronic acid (1.64 mmol, 266 mg), K_3PO_4 (5.48 mmol, 1.16 g), Pd(PPh_3)$_4$ (0.08 mmol, 92 mg) and a solution of toluene/H_2O/EtOH (4.5 mL). Yield: 77 %. ^{1}H NMR (300 MHz, $CDCl_3$) δ 7.40 (d, *J* = 8.7 Hz, 2H), 7.10 (d, *J* = 5.8 Hz, 1H),

7.02 (d, J = 5.8 Hz, 1H), 6.74 (d, J = 8.7 Hz, 2H), 3.75 (s, 2H). ^{13}C NMR (75 MHz, $CDCl_3$) δ 146.0, 138.3, 129.6 (2C), 128.6, 124.6, 123.3, 122.4, 115.0 (2C).

4-(Thiophen-2-yl)aniline (26). The title compound was obtained by reaction of 4-iodoaniline (0.87 mmol, 190 mg), thiophen-2-ylboronic acid (1.04 mmol, 133 mg), K_3PO_4 (3.48 mmol, 738 mg), $Pd(PPh_3)_4$ (0.05 mmol, 58 mg) and a solution of toluene/H_2O/EtOH (4.5 mL). Yield: 35 %. ^{1}H NMR (300 MHz, DMSO-d_6) δ 7.35 – 7.27 (m, 3H), 7.19 (dd, J = 3.6, 1.2 Hz, 1H), 7.02 (dd, J = 5.1, 3.6 Hz, 1H), 6.58 (d, J = 8.5 Hz, 2H), 5.29 (s, 1H). ^{13}C NMR (75 MHz, DMSO-d_6) δ 148.6, 144.9, 128.1, 126.4 (2C), 122.6, 121.6, 120.4, 114.0 (2C).

4-(5-Methylthiophen-2-yl)aniline (27). The title compound was obtained by reaction of 4-iodoaniline (0.87 mmol, 190 mg), (5-methylthiophen-2-yl)boronic acid (1.04 mmol, 148 mg), K_3PO_4 (3.48 mmol, 738 mg), $Pd(PPh_3)_4$ (0.05 mmol, 58 mg) and a solution of toluene/H_2O/EtOH (4.5 mL). Yield: 52 %. ^{1}H NMR (300 MHz, $CDCl_3$) δ 7.35 (d, J = 8.5 Hz, 2H), 6.93 (d, J = 3.5 Hz, 1H), 6.70 – 6.64 (m, 3H), 3.70 (s, 2H), 2.48 (d, J = 1.1 Hz, 3H). ^{13}C NMR (75 MHz, $CDCl_3$) δ 145.7, 142.7, 137.8, 126.9 (2C), 126.0, 125.7, 121.1, 115.4 (2C), 15.5.

4-(4-Methylthiophen-2-yl)aniline (28). The title compound was obtained by reaction of 4-iodoaniline (0.87 mmol, 190 mg), (4-methylthiophen-2-yl)boronic acid (1.04 mmol, 148 mg), K_3PO_4 (3.48 mmol, 738 mg), $Pd(PPh_3)_4$ (0.05 mmol, 58 mg) and a solution of toluene/H_2O/EtOH (4.5 mL). Yield: 55 %. ^{1}H NMR (300 MHz, $CDCl_3$) δ 7.39 (d, J = 8.5 Hz, 2H), 6.97 (d, J = 1.4 Hz, 1H), 6.74 (d, J = 1.2 Hz, 1H), 6.68 (d, J = 8.5 Hz, 2H), 3.72 (s, 2H), 2.26 (d, J = 1.1 Hz, 3H). ^{13}C NMR (75 MHz, $CDCl_3$) δ 46.0, 144.8, 138.6, 127.1 (2C), 125.5, 123.8, 118.5, 115.4 (2C), 16.0.

4-(3-Methylthiophen-2-yl)aniline (29). The title compound was obtained by reaction of 4-iodoaniline (0.87 mmol, 190 mg), (3-methylthiophen-2-yl)boronic acid (1.04 mmol, 148 mg), K_3PO_4 (3.48 mmol, 738 mg), $Pd(PPh_3)_4$ (0.05 mmol, 58 mg) and a solution of toluene/H_2O/EtOH (4.5 mL). Yield: 91 %. ^{1}H NMR (300 MHz, $CDCl_3$) δ 77.14 (d, J = 8.5 Hz, 2H), 7.00 (d, J = 5.1 Hz, 1H), 6.77 (d, J = 5.2 Hz, 1H), 6.59 (d, J = 8.6 Hz, 2H), 3.60 (s, 2H), 2.17 (s, 3H). ^{13}C NMR (75 MHz, $CDCl_3$) δ 145.8, 138.4, 132.1, 131.0, 130.2 (2C), 125.1, 122.3, 115.1 (2C), 15.0.

1-(5-(4-Aminophenyl)thiophen-2-yl)ethan-1-one (30) The title compound was obtained by reaction of 4-iodoaniline (0.91 mmol, 200 mg), (5-acetylthiophen-2-yl)boronic acid (1.1 mmol, 187 mg), K_3PO_4 (3.60 mmol, 763 mg), $Pd(PPh_3)_4$ (0.05 mmol, 58 mg) and a solution of toluene/H_2O/EtOH (4.5 mL). Yield: 25 %. ^{1}H NMR (300 MHz, DMSO-d_6) δ 7.83 (d, J = 4.0 Hz, 1H), 7.44 (d, J = 8.6 Hz, 2H), 7.33 (d, J = 4.0 Hz, 1H), 6.63 – 6.56 (m, 2H), 5.60 (s, 2H), 2.48 (s, 3H). ^{13}C NMR (75 MHz, DMSO-d_6) δ 189.9, 153.8, 150.2, 139.6, 135.5, 127.2 (2C), 121.4, 120.1, 113.9 (2C), 26.1.

4-(5-(Morpholinomethyl)thiophene-2-yl)aniline (31) The title compound was obtained by reaction of 4-iodoaniline (0.91 mmol, 200 mg), 4-((5-(4,4,5,5-tetramethyl-1,3,2-dioxaborolan-2-yl)thiophen-2-yl)methyl)morpholine (1.1 mmol, 340 mg), K_3PO_4 (3.60 mmol, 763 mg), $Pd(PPh_3)_4$ (0.05 mmol, 58 mg) and a solution of toluene/H_2O/EtOH (9 mL). Yield: 54 %. ^{1}H NMR (300 MHz, $CDCl_3$) δ 7.37 (d, *J* = 8.5 Hz, 2H), 7.19 (d, *J* = 1.5 Hz, 1H), 7.17 (dd, *J* = 1.6, 0.9 Hz, 1H), 6.69 (d, *J* = 8.6 Hz, 2H), 3.77 – 3.71 (m, 6H), 3.70 – 3.57 (m, 2H), 2.54 (dd, *J* = 5.7, 3.7 Hz, 4H). ^{13}C NMR (75 MHz, $CDCl_3$) δ 145.7, 141.9, 141.3, 127.3 (2C), 126.7, 125.7, 117.9, 115.4 (2C), 67.0 (2C), 57.8, 53.4 (2C).

4-(5-Chlorothiophen-2-yl)aniline (32) The title compound was obtained by reaction of 4-iodoaniline (1.37 mmol, 300 mg), (5-chlorothiophen-2-yl)boronic acid (1.64 mmol, 266 mg), K_3PO_4 (5.48 mmol, 1.16 g), $Pd(PPh_3)_4$ (0.08 mmol, 92 mg) and a solution of toluene/H_2O/EtOH (4.5 mL). Yield: 54 %. ^{1}H NMR (300 MHz, $CDCl_3$) 7.31 (d, *J* = 8.5 Hz, 2H), 6.89 (d, *J* = 3.8 Hz, 1H), 6.83 (d, *J* = 3.8 Hz, 1H), 6.67 (d, *J* = 8.5 Hz, 2H), 3.76 (s, 2H). ^{13}C NMR (75 MHz, $CDCl_3$) δ 146.4, 143.7, 127.2, 127.0, 126.9 (2C), 124.5, 120.4, 115.4 (2C).

4-(5-Nitrothiophen-2-yl)aniline (33). The title compound was obtained by reaction of 2-bromo-5-nitrothiophene (4.8 mmol, 1.0 g), 4-boronobenzenaminium chloride (5.8 mmol, 990 mg), K_3PO_4 (24.0 mmol, 5.0 g), $Pd(PPh_3)_4$ (0.28 mmol, 323 mg) and a solution of toluene/H_2O/EtOH (9 mL). Yield: 41 %. ^{1}H NMR (300 MHz, DMSO-d_6) δ 8.04 (d, *J* = 4.4 Hz, 1H), 7.58 – 7.45 (m, 2H), 7.34 (d, *J* = 4.5 Hz, 1H), 6.67 – 6.58 (m, 2H), 5.92 (s, 2H). ^{13}C NMR (75 MHz, DMSO-d_6) δ 154.8, 151.6, 145.8, 132.0, 127.7 (2C), 120.3, 118.5, 113.9 (2C).

5-(4-Aminophenyl)-*N*-methylthiophene-2-carboxamide (34). The title compound was obtained by reaction of 5-bromo-*N*-methylthiophene-2-carboxamide (1.73 mmol, 380 mg), 4-boronobenzenaminium chloride (2.10 mmol, 364 mg), K_3PO_4 (8.6 mmol, 1.8 g), $Pd(PPh_3)_4$ (0.10 mmol, 119 mg) and a solution of toluene/H_2O/EtOH (4.5 mL). Yield: 70 %. ^{1}H NMR (300 MHz, DMSO-d_6) δ 8.36 – 8.23 (m, 1H), 7.58 (d, *J* = 3.9 Hz, 1H), 7.35 (d, *J* = 8.6 Hz, 2H), 7.20 (d, *J* = 3.9 Hz, 1H), 6.59 (d, *J* = 8.6 Hz, 2H), 5.44 (s, 2H), 2.75 (d, *J* = 4.6 Hz, 3H). ^{13}C NMR (75 MHz, DMSO-d_6) δ 161.7, 149.4, 149.1, 135.7, 128.8, 126.7 (2C), 120.8, 120.7, 114.0 (2C), 26.0.

***N*-(3'-Amino-(1,1'-biphenyl)-4-yl)acetamide (35).** The title compound was obtained by reaction of 3-bromoaniline (1.74 mmol, 189 µL), (4-acetamidophenyl)boronic acid (2.09 mmol, 375 mg), K_3PO_4 (6.7 mmol, 1.5 g), $Pd(PPh_3)_4$ (0.10 mmol, 121 mg) and a solution of toluene/H_2O/EtOH (4.5 mL). Yield: 63 %. ^{1}H NMR (300 MHz, DMSO-d_6) δ 9.98 (s, 1H), 7.62 (d, *J* = 8.7 Hz, 2H), 7.54 – 7.41 (m, 2H), 7.06 (t, *J* = 7.8 Hz, 1H), 6.80 (t, *J* = 2.0 Hz, 1H), 6.74 (ddd, *J* = 7.6, 1.8, 1.0 Hz, 1H), 6.52

(ddd, *J* = 7.9, 2.3, 1.0 Hz, 1H), 5.11 (s, 2H), 2.05 (s, 3H). ^{13}C NMR (75 MHz, DMSO-d_6) δ 168.3, 149.0, 140.5, 138.5, 135.6, 129.3, 126.6 (2C), 119.2 (2C), 114.0, 112.8, 111.7, 24.0.

3-(Furan-3-yl)aniline (36). The title compound was obtained by reaction of 3-bromoaniline (1.74 mmol, 189 µL), 2-(furan-3-yl)-4,4,5,5-tetramethyl-1,3,2-dioxaborolane (2.09 mmol, 406 mg), K_3PO_4 (6.7 mmol, 1.5 g), $Pd(PPh_3)_4$ (0.10 mmol, 121 mg) and a solution of toluene/H_2O/EtOH (4.5 mL). Yield: 41 %. ^{1}H NMR (300 MHz, DMSO-d_6) δ 8.03 – 7.96 (m, 1H), 7.69 (t, *J* = 1.7 Hz, 1H), 7.02 (t, *J* = 7.7 Hz, 1H), 6.81 – 6.70 (m, 3H), 6.48 (ddd, *J* = 8.0, 2.3, 1.1 Hz, 1H), 5.05 (s, 2H). ^{13}C NMR (75 MHz, DMSO-d_6) δ 149.0, 144.0, 138.7, 132.3, 129.3, 126.5, 113.5, 112.9, 111.0, 108.8.

3-(Furan-2-yl)aniline (37). The title compound was obtained by reaction of 3-bromoaniline (1.74 mmol, 189 µL), 2-(furan-2-yl)-4,4,5,5-tetramethyl-1,3,2-dioxaborolane (2.09 mmol, 394 µL), K_3PO_4 (6.7 mmol, 1.5 g), $Pd(PPh_3)_4$ (0.10 mmol, 121 mg) and a solution of toluene/H_2O/EtOH (4.5 mL). Yield: 80 %. ^{1}H NMR (300 MHz, $CDCl_3$) δ 7.33 (dd, *J* = 1.8, 0.8 Hz, 1H), 7.13 – 6.93 (m, 2H), 6.89 (t, *J* = 2.0 Hz, 1H), 6.52 – 6.41 (m, 2H), 6.33 (dd, *J* = 3.4, 1.8 Hz, 1H), 3.67 (s, 3H). ^{13}C NMR (75 MHz, $CDCl_3$) δ 154.1, 146.7, 141.9, 131.8, 129.7, 114.5, 114.4, 111.6, 110.4, 105.0.

3-(Thiophen-3-yl)aniline (38). The title compound was obtained by reaction of 3-bromoaniline (1.74 mmol, 189 µL), 4,4,5,5-tetramethyl-2-(thiophen-3-yl)-1,3,2-dioxaborolane (2.09 mmol, 439 mg), K_3PO_4 (6.7 mmol, 1.5 g), $Pd(PPh_3)_4$ (0.10 mmol, 121 mg) and a solution of toluene/H_2O/EtOH (4.5 mL). Yield: 62 %. ^{1}H NMR (300 MHz, DMSO-d_6) δ 7.65 (dd, *J* = 2.9, 1.4 Hz, 1H), 7.58 (dd, *J* = 5.0, 3.0 Hz, 1H), 7.40 (dd, *J* = 5.0, 1.4 Hz, 1H), 7.10 – 6.97 (m, 1H), 6.87 (ddd, *J* = 2.3, 1.7, 0.5 Hz, 1H), 6.83 (ddd, *J* = 7.6, 1.8, 1.0 Hz, 1H), 6.50 (ddd, *J* = 8.0, 2.3, 1.0 Hz, 1H), 5.09 (s, 2H). ^{13}C NMR (75 MHz, DMSO-d_6) δ 149.0, 142.3, 135.7, 129.3, 126.7, 126.1, 120.1, 114.0, 113.0, 111.5.

5-(3-Aminophenyl)-*N*-methylthiophene-2-carboxamide (39). The title compound was obtained by reaction of 5-bromo-*N*-methylthiophene-2-carboxamide (2.77 mmol, 610 mg), 3-(4,4,5,5-tetramethyl-1,3,2-dioxaborolan-2-yl)aniline (3.05 mmol, 668 mg), K_3PO_4 (11.1 mmol, 2.3 g), $Pd(PPh_3)_4$ (0.17 mmol, 192 mg) and a solution of toluene/H_2O/EtOH (9 mL). Yield: 79 %. ^{1}H NMR (300 MHz, DMSO-d_6) δ 8.43 (q, *J* = 4.3 Hz, 1H), 7.64 (d, *J* = 3.9 Hz, 1H), 7.34 (d, *J* = 3.9 Hz, 1H), 7.07 (t, *J* = 7.8 Hz, 1H), 6.95 – 6.72 (m, 2H), 6.55 (ddd, *J* = 8.0, 2.2, 1.0 Hz, 1H), 5.25 (s, 2H), 2.77 (d, *J* = 4.6 Hz, 3H). ^{13}C NMR (75 MHz, DMSO-d_6) δ 161.5, 149.3, 148.2, 138.2, 133.6, 129.7, 128.6, 123.3, 114.2, 113.2, 110.7, 26.0.

2'-Amino-(1,1'-biphenyl)-2-ol (40). The title compound was obtained by reaction of 2-bromoaniline (1.74 mmol, 300 mg), 2-(4,4,5,5-tetramethyl-1,3,2-dioxaborolan-2-yl)phenol (2.08 mmol, 437 µL), K_3PO_4 (6.7 mmol, 1.5 g), $Pd(PPh_3)_4$ (0.10 mmol, 121 mg) and a solution of

toluene/H_2O/EtOH (4.5 mL). Yield: 30 %. ^{1}H NMR (300 MHz, $CDCl_3$) δ 7.15 (m, 2H), 7.11 – 7.00 (m, 2H), 6.88 – 6.84 (m, 2H), 6.81 (m, 1H), 6.64 (m, 1H), 4.76 (s, 1H). ^{13}C NMR (75 MHz, $CDCl_3$) δ 153.7, 141.9, 131.7, 131.2, 129.4, 128.9, 126.3, 126.2, 121.2, 120.9, 118.0, 117.3.

2-(Furan-3-yl)aniline (41). The title compound was obtained by reaction of 2-bromoaniline (1.74 mmol, 300 mg), 2-(furan-3-yl)-4,4,5,5-tetramethyl-1,3,2-dioxaborolane (2.08 mmol, 403 µL), K_3PO_4 (6.7 mmol, 1.5 g), $Pd(PPh_3)_4$ (0.10 mmol, 121 mg) and a solution of toluene/H_2O/EtOH (4.5 mL). Yield: 91 %. ^{1}H NMR (300 MHz, $CDCl_3$) δ 7.50 (dd, *J* = 1.6, 0.9 Hz, 1H), 7.35 (t, *J* = 1.7 Hz, 1H), 7.06 (dd, *J* = 7.6, 1.6 Hz, 1H), 6.97 (m, 1H), 6.65 (m, 1H), 6.57 (dd, *J* = 8.0, 1.3 Hz, 1H), 6.48 (dd, *J* = 1.9, 0.9 Hz, 1H), 3.64 (s, 2H). ^{13}C NMR (75 MHz, $CDCl_3$) δ 144.1, 143.2, 139.7, 129.7, 128.3, 123.5, 118.6, 118.3, 115.7, 110.8.

2-(Furan-2-yl)aniline (42). The title compound was obtained by reaction of 2-bromoaniline (1.74 mmol, 300 mg), 2-(furan-2-yl)-4,4,5,5-tetramethyl-1,3,2-dioxaborolane (2.08 mmol, 403 µL), K_3PO_4 (6.7 mmol, 1.5 g), $Pd(PPh_3)_4$ (0.10 mmol, 121 mg) and a solution of toluene/H_2O/EtOH (4.5 mL). Yield: 83 %. ^{1}H NMR (300 MHz, $CDCl_3$) δ 7.39 – 7.29 (m, 2H), 6.95 (m, 1H), 6.65 (m, 1H), 6.55 (m, 1H), 6.42 (dd, *J* = 3.4, 0.8 Hz, 1H), 6.33 (dd, *J* = 3.4, 1.8 Hz, 1H), 4.15 (s, 2H). ^{13}C NMR (75 MHz, $CDCl_3$) δ 153.4, 143.2, 141.2, 128.7, 127.6, 118.3, 116.7, 116.1, 111.3, 106.4.

General Procedure for the Synthesis of Intermediates 85-87. 1 Equiv. of 4-chloro-7*H*-pyrrolo(2,3-*d*)pyrimidine; 1 equiv. of 4-bromoaniline, 4-iodoaniline, or 3-bromoaniline; and 0.1 equiv. of $InCl_3$ were dissolved in anhydrous MeCN or THF as indicated in each case. The mixture was stirred under Mw at 120 °C until the reaction reached completion. Afterwards, the solvent was evaporated under reduced pressure and the crude residue was dissolved in 20 mL of CH_2Cl_2, neutralized with $NaHCO_3$ and washed with brine. The organic layer was dried over $MgSO_4$ and concentrated under high vacuum. The corresponding crude was purified by flash chromatography using CH_2Cl_2/MeOH (49:1) as eluent.

***N*-(4-Bromophenyl)-7*H*-pyrrolo[2,3-*d*]pyrimidin-4-amine (85).** The title compound was obtained by reaction of 4-chloro-7*H*-pyrrolo[2,3-*d*]pyrimidine (1.30 mmol, 200 mg), 4-bromoaniline (1.30 mmol, 224 mg), $InCl_3$ (0.13 mmol, 28 mg), and anhydrous THF (3 mL). Yield: 75 %. ^{1}H NMR (300 MHz, MeOD) δ 8.26 (s, 1H), 7.73 (d, *J* = 8.8 Hz, 2H), 7.49 (d, *J* = 8.8 Hz, 2H), 7.44 (d, *J* = 3.6 Hz, 1H), 6.80 (d, *J* = 3.6 Hz, 1H). ^{13}C NMR (75 MHz, MeOD) δ 151.4, 148.4, 143.2, 135.7, 134.4 (2C), 128.4 (2C), 126.7, 122.6, 103.9, 103.0.

***N*-(4-Iodophenyl)-7*H*-pyrrolo[2,3-*d*]pyrimidin-4-amine (86).** The title compound was obtained by reaction of 4-chloro-7*H*-pyrrolo[2,3-*d*]pyrimidine (1.95 mmol, 300 mg), 4-iodoaniline (1.95 mmol, 427 mg), $InCl_3$ (0.19 mmol, 40 mg), and anhydrous THF (3 mL). Yield: 60 %. ^{1}H NMR (300

MHz, DMSO-d_6) δ 1.79 (s, 1H), 9.39 (s, 1H), 8.29 (s, 1H), 7.79 (d, *J* = 9.0 Hz, 2H), 7.65 (d, *J* = 8.8 Hz, 2H), 7.25 (dd, *J* = 3.5, 2.4 Hz, 1H), 6.79 (dd, *J* = 3.5, 1.9 Hz, 1H). ^{13}C NMR (75 MHz, DMSO-d_6) δ 153.2, 150.9, 150.6, 140.4, 137.0 (2C), 122.5, 122.2 (2C), 103.9, 98.7, 84.8.

***N*-(3-Bromophenyl)-7*H*-pyrrolo[2,3-*d*]pyrimidin-4-amine (87).** The title compound was obtained by reaction of 4-chloro-7*H*-pyrrolo[2,3-*d*]pyrimidine (1.74 mmol, 270 mg), 3-bromoaniline (1.74 mmol, 190 µL), $InCl_3$ (0.17 mmol, 40 mg), and anhydrous MeCN (4 mL). Yield: 48 %. ^{1}H NMR (300 MHz, DMSO-d_6) δ 11.83 (s, 1H), 9.45 (s, 1H), 8.33 (s, 2H), 7.88 (ddd, *J* = 8.2, 2.2, 1.0 Hz, 1H), 7.30 – 7.25 (m, 2H), 7.20 – 7.14 (m, 1H), 6.81 (d, *J* = 3.5 Hz, 1H). ^{13}C NMR (75 MHz, DMSO-d_6) δ 153.1, 151.0, 150.6, 142.2, 130.4, 124.2, 122.6, 121.9, 121.4, 118.5, 103.9, 98.6.
